# High-resolution chromatin mapping reveals that CTCF anchors meiotic loops to the chromosome axis

**DOI:** 10.1038/s41467-026-73644-6

**Published:** 2026-07-28

**Authors:** Gang Cheng, Xin Li, Kevin Brick, Benjamin Alleva, Mini Huang, Kwan-Wood Gabriel Lam, Florencia Pratto, R. Daniel Camerini-Otero

**Affiliations:** 1 Genetics and Biochemistry Branch, NIDDK, National Institutes of Health, Bethesda, MD USA; 2 Present Address: KariusDX, Redwood City, USA; 3 Present Address: Zhongshan School of Medicine, Sun Yat-sen University Shenzhen Campus, Shen Zhen, China; 4 Present Address: Computational Biology and Genomics Core, Laboratory of Genetics and Genomics, NIA, National Institutes of Health, Baltimore, MD USA

**Keywords:** Chromosomes, Functional genomics, Spermatogenesis, Chromatin structure

## Abstract

When precursor germ cells enter meiosis, chromosomes condense into an array of chromatin loops. Using Hi-C and Micro-C, we explored the trajectory of chromatin reorganization from mouse spermatogonia to spermatocytes at the highest temporal and spatial resolution to date. We show that meiotic chromatin reorganization, characterized by a shift from mitotic-to-meiotic cohesins, precedes the traditionally defined meiotic entry point (meiotic G1/S-phase). We find that large-scale A/B compartments are lost during prophase I, while local sub-compartments and interactions between regulatory elements of gene expression remain. For the first time, we detect distinct looping positions in mammalian meiosis I prophase. These loops are anchored by a subset of CTCF sites at the base of chromatin loops but formed without enriched interactions within the intervening regions, deviating from the typical pattern observed in somatic interphase cells. Our findings allow us to reconcile the current discrepancy in the length of meiotic chromatin loops and support a model where meiotic loops are formed by association of existing loops modulated by CTCF rather than by loop extrusion.

## Abstract (web summary)

Here, the authors use high-resolution chromatin contact maps from mouse sperm precursors to reveal an intermediate folding state for chromosomes prior to meiotic entry and identify distinct looping positions associated with CTCF.

## Introduction

The spatial organization of genomes plays pivotal roles in biological functions^1–3^. The organization of the mammalian genome is hierarchical. Chromatin structures are spatially organized into distinct scales ranging from chromosome territories, A/B compartments, topologically associating domains (TADs) to chromatin loops^4^.

Meiosis is central to sexual reproduction. It generates haploid gametes through two cell divisions after a single DNA replication. A hallmark of meiosis is homologous recombination, which promotes genetic diversity and ensures the accurate segregation of homologous chromosomes. Errors in meiosis represent a leading cause of mental disabilities, miscarriages, and infertility^5–7^. During meiosis, chromosomes undergo dramatic structural changes needed for the proper alignment and segregation of homologs^8^. As cells enter meiosis, chromosomes are organised as linear loop arrays that emanate from a proteinaceous axis which include cohesins containing meiotic-specific subunits and axial core elements^9^. Such reorganization leads to linearized chromosomes while retaining active transcription^10^.

Until recently, most understanding of meiotic chromatin structure came from cytological studies^8^, but technological advances have enabled the interrogation of meiotic chromosome organization through Chromosome Conformation Capture (3C)-based methods^11,12^. Recent studies have used Hi-C to detect genome-wide chromatin interactions during mammalian spermatogenesis. Studies both in rhesus monkey and mouse have shown that TADs break down as cells enter meiosis I prophase^13–19^. This attenuation of TADs is accompanied by a progressive increase in loop size, from ~500 kb to a maximum of 1.8 Mb in late meiosis I prophase^15^. In yeast, loops are prominently positioned, and their bases are associated with the chromosome axis and depend on the meiotic specific cohesin, REC8, and its DNA binding preferences^9,20,21^. However, this type of distinct loop has not yet been detected in mammalian spermatocytes. In mammalian meiotic cells, the earliest stage assessed to date has been meiotic S-phase, where loops are already larger than in mitotic cells^15^. Technical difficulties in isolating stages preceding meiotic S-phase have prevented studies investigating the chromatin reorganization that occurs before meiosis I prophase. Moreover, fine-scale chromosomal interactions were not examined. These types of analyses can be conducted using Micro-C, a derivative of Hi-C in which the genome is fragmented by micrococcal nuclease, rather than restriction enzymes, providing a higher-resolution view of chromosomal interactions^22–24^.

In this work, we use a unique nuclei sorting strategy^25^ to isolate germ cells at stages ranging from spermatogonia, through the mitotic-to-meiotic transition stages, and up to the end of meiosis I prophase. Using in-situ Hi-C^26^ and Micro-C^24^, we determine the trajectory of chromatin reorganization at the highest temporal and spatial resolution to date. By focusing on the earliest stage where chromatin becomes “meiotic”, we reveal novel features underlying chromatin organization in meiosis I prophase.

## Results

### Isolation of stage-specific nuclei through spermatogenesis

**Fig. 1:**
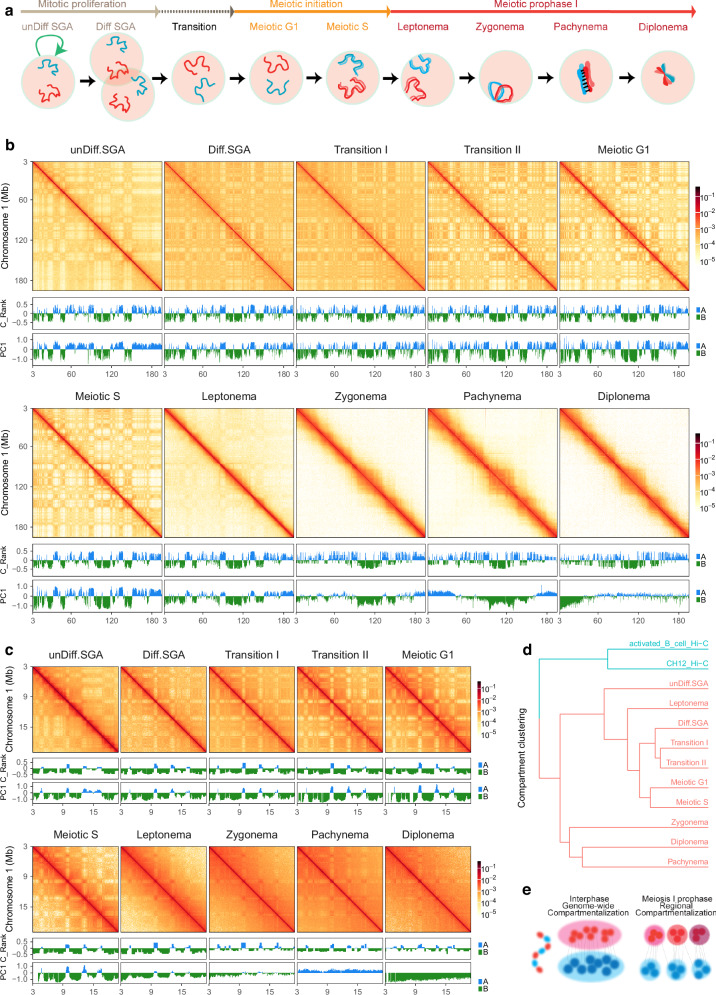
Genome-wide chromatin reorganization throughout spermatogenesis. **a** A schematic of cell populations examined in this study. **b** Genome-wide chromatin reorganization of chromosome 1 derived from Hi-C data. Top: Matrices of chromosome 1 at a resolution of 100 Kb. Middle: Track shows the compartment ranks generated by Calder^34^. To compare with the eigenvector values, we subtracted 0.5 from each rank. Bottom: Track shows the eigenvector values for the first principal component of the Hi-C matrix calculated at a resolution of 100 Kb. Blue: active regions; Green: inactive regions. Calder and PC1 generated comparable profiles, except for the zygonema, pachynema, and diplonema stages. **c** A snapshot of Hi-C matrices at chromosome 1: 3–20 Mb. Top: Matrices were plotted at a resolution of 50 Kb. Middle: Track shows the compartment ranks generated by Calder^34^. To compare with the eigenvector values, we subtracted 0.5 from each rank. Bottom: Track shows the eigenvector values for the first principal component of the Hi-C matrix calculated at a resolution of 100 Kb. Blue: active regions; Green: inactive regions. Both Calder and PC1 were able to differentiate compartment intervals up to the leptotene stage. In the zygonema, pachynema and diplonema stages, where only a local “checkerboard”-like pattern was observed, Calder maintains the ability to identify compartment intervals whereas PC1 was unable to distinguish the genomic regions with visible differences in contact preference. **d** Clustering of all stages based on compartmental annotation derived from Calder^34^. **e** A schematic of the local compartmentalization during meiosis I prophase. The schematic was adapted from^92^. Left: Blue and red represent alternating A (active) and B (inactive) compartment intervals along a chromatin fiber. Middle: Genome-wide Compartmentalization into two big domains. Right: Compartmentalization is restrained to local interactions, forming small domains.

To identify chromatin structure changes during spermatogenesis, we isolated pure populations of germline nuclei using fluorescence-activated nucleus sorting^25^ (Fig. 1a and Supplementary Fig. 1a). Nuclei from the germ cells that preceded meiotic entry were identified using a sorting paradigm that relies on DNA content and a combination of intranuclear proteins associated with each stage (Supplementary Fig. 1a). Undifferentiated spermatogonia (unDiff.SGA) were identified as 2C nuclei expressing the PLZF (promyelocytic leukemia zinc finger) protein^27,28^ (Supplementary Fig. 1a–d). Other pre-meiotic populations up to meiotic G1 were identified by scoring for DMRT1, which suppresses meiotic entry and is expressed throughout spermatogonial development^29^ and for STRA8, which triggers meiotic entry but is expressed both in spermatogonia^30,31^ and in cells that enter meiosis I prophase^31^ (Supplementary Fig. 1a–d). Meiotic S-phase, leptotene, zygotene, pachytene, and diplotene nuclei were isolated as described^25,32^. This approach generates populations with at least 85% purity and up to 95%^25^.

We performed in-situ Hi-C on all stages of isolated nuclei. All replicates were pooled after confirming reproducibility (Supplementary Fig. 2). To examine the interactions at high resolution, we employed Micro-C^22–24^, on a subset of nuclei isolated from meiotic S-phase through diplonema and generated contact maps containing up to 3 billion interactions (Supplementary table 1), sufficient to identify most detectable loops^33^.

### Local compartmentalization of meiosis I prophase chromosomes is preserved despite the loss of higher-order structures

During interphase, the inherent affinity within active (A) and inactive (B) genomic regions leads to the spatial partitioning of chromosomes into two distinct compartments that manifest as a genome-wide “checkerboard”-like pattern in Hi-C maps^11^. Most studies (except for Vara et al.) have reported that the typical A and B compartments are still present during meiosis^9,10,13–19^ (Supplementary table 2). Given that meiotic chromosomes are reorganised into axis-loop arrays and are individualised, these observations were unexpected. We revisited this question by leveraging the high temporal and spatial resolution of our data.

We found that the whole-chromosome “checkerboard”-like pattern was present in germline interphase cells and persisted up to when cells entered the meiotic S-phase (Fig. 1b). The pattern of frequent far-cis interactions diminished as cells entered leptonema (Fig. 1b and Supplementary Figs. 4a and 5a) and vanished entirely at pachynema (Fig. 1b and Supplementary Figs. 4a and 5a). Using principal component analysis (PCA), we could assign A/B compartment intervals in genomes from undifferentiated spermatogonia to leptonema (Fig. 1b, c). However, A/B compartment differentiation became less distinct in zygonema and was completely lost in pachynema and diplonema (Fig. 1b, c). These observations were consistent across all replicates in both our Hi-C and Micro-C data (Supplementary Figs. 4a, b and 6). To explore why A/B compartments were detected in previous studies (Supplementary table 2), we examined the effect of cell population purity on compartment detection by adding meiotic S-phase interaction data into pachytene interaction data. Even a 5% of in-silico “contamination” resulted in the appearance of typical A/B compartments by PCA (Supplementary Fig. 7). Furthermore, most previous studies performed cell sorting without fixation. Prolonged in vitro manipulation of live spermatocytes might have altered the intrinsic properties of cells. In contrast, our data and that of Vara et al., were derived from cells that were fixed immediately after dissection, likely ensuring better preservation of chromatin structure.

When cells enter meiosis, telomeres attach to the nuclear envelope. And at the leptotene-zygotene transition, they move to one region to form what is called the “bouquet” structure. We detected significantly higher inter-chromosomal interactions between sub telomeric regions at leptotene as reported by Vara et al. A visual inspection of the inter-chromosomal interactions revealed an X-shaped pattern that was previously thought to reflect the bouquet structure. This pattern is visible from leptotene until diplotene where the bouquet is not present. The quantification of the inter-chromosomal interactions at the ends of the chromosomes and the persistence of the X-shaped pattern beyond leptotene and zygotene indicate that this reflects interaction between individualised chromosomes constrained by the attachment of telomeres at the nuclear envelope, but not necessarily a bouquet structure (Supplementary Fig. 3)

Despite the loss of whole-chromosome A/B compartments, we observed a local “checkerboard”-like pattern^18,19^. We used Calder^34^, an algorithm designed to use short-range intra-chromosomal interactions to classify domains (Fig. 1b, c and Supplementary Fig. 4a, b), and identified local compartments across all stages, from Hi-C and/or Micro-C data. The strength of interactions varies, and different associations emerge when clustering by compartments (Fig. 1c, d and Supplementary Fig. 4b). Importantly, and distinct from previous reports^18^, we find that these fine-scale compartments are not exclusive to the meiosis I prophase. Instead, compartment intervals maintain their shorter-distance contact preferences in all the examined sub-stages. Altogether, these data suggests that constraints on long-distance interactions, potentially due to the axis-loop array organization, prevents whole chromosomes from segregating into large compartments, but domains of smaller size are preserved (Fig. 1e and Fig. 7).

### Meiotic chromatin features appear before the traditional meiotic entry point, associated with coordinated changes in mitotic and meiotic cohesins

**Fig. 2:**
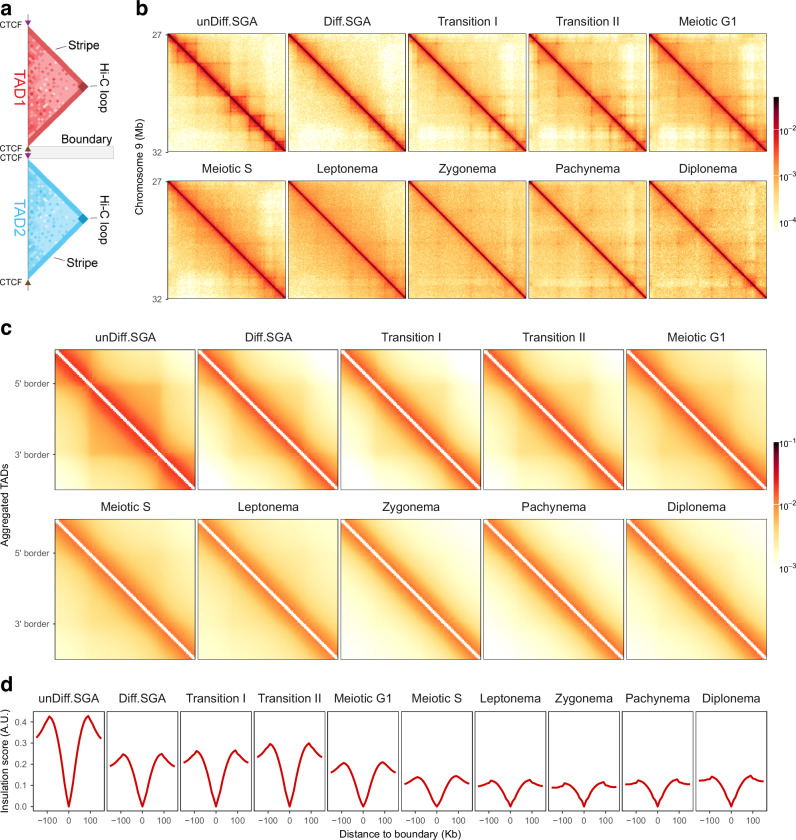
Chromatin folding changes during the mitotic-to-meiotic transition. **a** A schematic of typical TAD structure in Hi-C matrices. Interactions are enriched within TAD1 and TAD2; and isolated between TAD1 and TAD2. CTCF binding sites with forward and backward oriented motifs are indicated by arrows. **b** Hi-C matrices of chromosome 9 at a resolution of 10 Kb (region: 27 Mb to 32 Mb). **c** Aggregated interactions of conserved TADs. TAD boundaries were identified from both unDiff.SGA and mouse embryonic stem (mES) cells^88^ using Cooltools at a resolution of 10 Kb with a window size of 100 Kb. In order to pinpoint conserved boundaries, we initially extended 10 Kb on both sides of the boundaries identified from unDiff.SGA. Boundaries identified in unDiff.SGA that overlapped with boundaries identified in mES cells were classified as conserved boundaries. TAD domains in unDiff.SGA were defined as regions bordered by the two nearest boundaries. Only the TADs in unDiff.SGA defined by the conserved boundaries were used for this analysis. **d** Average insulation scores flanking 150 Kb of conserved TAD boundaries. The conserved TAD boundaries were defined by overlapping the boundaries identified in unDiff.SGA and mES cells. The insulation scores were called at a resolution of 10 Kb with a window size of 100 Kb; the minima were normalised to zero.

**Fig. 3:**
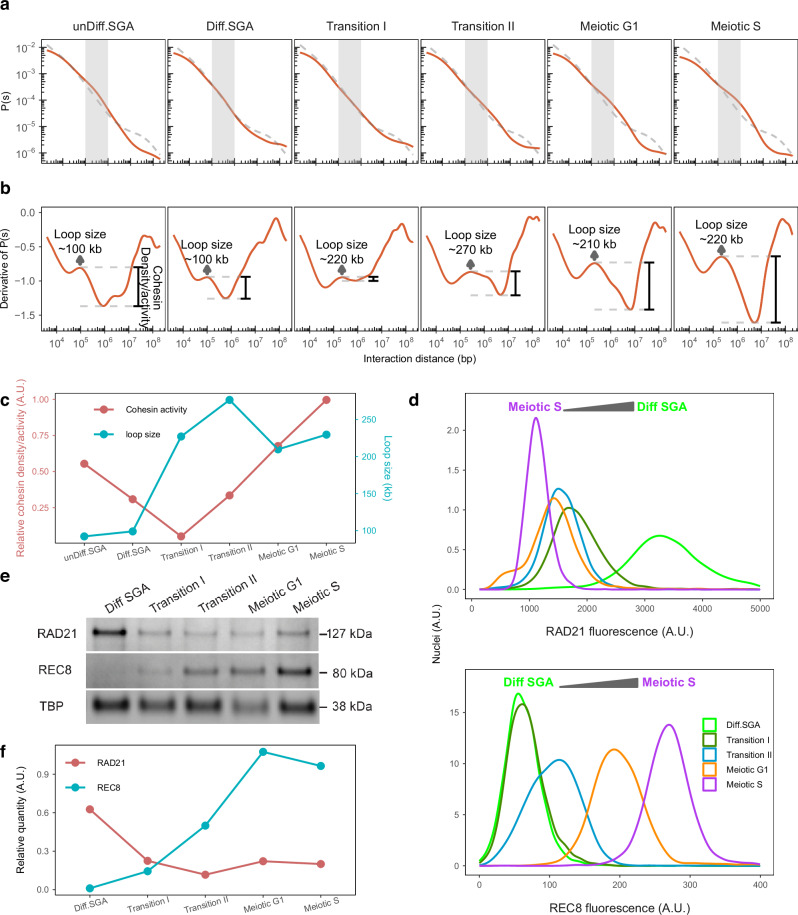
Chromatin reorganization is associated with changes in cohesin during the mitotic-to-meiotic transition. **a** The contact probabilities are plotted as a function of interaction distance *P(s)*. The gray regions indicate the scale of TADs. A *P(s)* curve derived from cells defective in cohesin loading and/or lacking loop extrusion activities is shown as a reference (gray line). **b** The derivatives of their corresponding *P(s)* curves. The first local maximums are indicated as arrows, designating the estimated average loop size. The depth of the valley is indicated by the black bar (cohesin density/activity). **c** Quantitative summary of Fig. 3b. The relative cohesin density/activity was determined by subtracting the derivative value of the first “bump” and the minimum value. The mean value of biological replicates was calculated (n = 2 to 3); loop size is the contact distance of the first “bump”. The mean loop size was calculated (n = 2 to 3). **d** The mitotic and meiotic cohesin abundance at “pre-meiotic” stages. Top: The profile of RAD21 fluorescence detected by flow cytometry from Diff.SGA to Meiotic S-phase. Bottom: The profile of REC8 fluorescence detected by flow cytometry from Diff.SGA to Meiotic S-phase. **e** Western blot analysis of mitotic kleisin RAD21 and meiotic kleisin REC8 in different stages preceding meiosis I prophase. TBP (TATA box binding protein) was used as the loading control. **f** Quantification of the Western blot. Protein abundance at each stage was normalised to the levels of TBP. The mean value of two replicates was calculated. Note: The relative quantity of REC8 and RAD21 proteins is not directly comparable due to the use of different antibodies. Only changes across stages for the same protein should be interpreted. Source data are provided as a Source Data file.

An outstanding question regarding chromosome reorganization during meiosis is determining at which stage these changes are initiated. Previous work has described the loss of TADs as cells enter meiosis^9,10,13–19^. These are typical interphase structures, visible as squares in the matrices where interactions occur within domains rarely extending to regions beyond their boundaries^35^ (Fig. 2a). While the undifferentiated spermatogonia (unDiff.SGA) interaction map displayed typical TADs, an attenuation of these structures was already evident in differentiating spermatogonia (Diff.SGA) (Fig. 2b, c). TADs were further weakened upon initiation of meiosis (meiotic G1/S), becoming barely visible by leptonema (Fig. 2b, c). Insulation between TADs was quantified as the difference between local maxima and minima of the insulation score^36^. Quantification using insulation profiles confirmed our observations (Fig. 2d). We sought to identify the mechanism that drives the changes in chromatin conformation during mitotic-to-meiotic transition. Properties of chromosome folding can be assessed by calculating *P(s)*, the contact probability as a function of genomic distance^37^. When looping activity is impaired due to NIPBL deficiency, the *P(s)* curve decays steeply at short genomic distances (Fig. 3a, gray dashed line). The appearance of a “bump” above this baseline indicates the presence of cohesin-mediated looping^38^. We observed a gradual change in the curves at the scale of TAD as cells progressed from undifferentiated spermatogonia into meiosis, indicating a shift in dynamics of loop formation (Fig. 3a). The shoulder then moves towards longer distances as cells continue into meiosis I prophase.

The derivative of *P(s)* can reveal the average loop lengths and the density of loops on chromosomes^37^. As this estimation is based on a polymer simulation, the derived loop size is referred to as simulated loop size. Indicated by the first local maxima of the derivative, unDiff.SGA had loops with a simulated size of 100 Kb (Fig. 3b, c), similar to that found in most somatic interphase cells^37,39,40^. The simulated loop size remained largely constant as cells transitioned to Diff.SGA (Fig. 3b, c). However, the “valley” following the first “bump” became shallower in Diff.SGA (Fig. 3b); and the derivative curve was nearly flat upon entering into the Transition I stage (Fig. 3b). According to polymer simulations^37^, this indicates a decrease in cohesin loading and/or loop extrusion activity. In addition, the remaining loops were more than two times larger than those of the previous stage, averaging around 220 Kb (Fig. 3b, c). As cells progressed into meiosis, the simulated loop size persisted above 200 Kb, with cohesin density/activity increasing until the meiotic S-phase stage (Fig. 3b, c). Upon entering meiosis I prophase, loop size kept increasing, starting at 360 Kb in leptonema, rising to 900 Kb in zygonema, reaching the maximum at pachynema with a stimulated loop size of 1.3 Mb (Supplementary Fig. 5b, c). Contrary to previous work^15,18^, our data indicates a decrease in stimulated loop size in diplonema, averaging approximately 700 kb (Supplementary Fig. 5b, c). As pointed out previously^9^, chromatin loop sizes derived from Hi-C do not align with those calculated from cytological studies, but the relative changes can be informative of the underlying changes in structure throughout stages.

Interestingly, transition stage I derivative curves mirrored the pattern seen during telophase when somatic cells exit mitosis^40^. During telophase, there is a condensin-to-cohesin transition, where condensins are evicted from the chromosomes before cohesin binding, leading to an intermediate state of chromatin folding. Similarly, during entry to meiosis, cohesin’s kleisin transitions from RAD21 to meiotic specific kleisins^41^. Altogether, this suggests that cohesin dynamics are disrupted in the transition stages.

To test this hypothesis, we tracked the changes in the protein levels of mitotic and meiotic cohesin subunits across different stages. RAD21, the α-kleisin of the mitotic cohesin complex, declined immediately as cells entered Transition I, and remained at a low level in subsequent stages (Fig. 3d and Supplementary Fig. 8a). Conversely, REC8, a meiotic-specific α-kleisin, began to increase at Transition II, peaking at meiotic S-phase (Fig. 3d and Supplementary Fig. 8b). The shift in protein levels of RAD21 and REC8 was further confirmed by Western blot (Fig. 3e, f). A rise in RAD21L, another meiotic-specific α-kleisin, was not observed until meiotic S-phase (Supplementary Fig. 8c), implicating REC8 as the only meiotic-specific α-kleisin involved with the chromatin structural changes observed during the mitotic-to-meiotic transition. At the same time, no significant changes in CTCF levels were observed (Supplementary Fig. 8d).

### CTCF shapes meiotic chromatin organization

In interphase cells, the interplay between CTCF and cohesins shape architectural features such as TADs, stripes and dots detected by Hi-C (Fig. 5e (top right)). Live cell imaging studies showed that the loops within TADs are short-lived; and they exhibited greater stability when anchored by convergent CTCFs^42,43^. Although cohesins can block each other^44,45^, closely positioned loops are rare due to their transient nature^46,47^. This strongly contrasts the meiotic axis-loop array organization, where consecutive cohesin-mediated loops are densely anchored to the proteinaceous axis^8,9^.

**Fig. 4:**
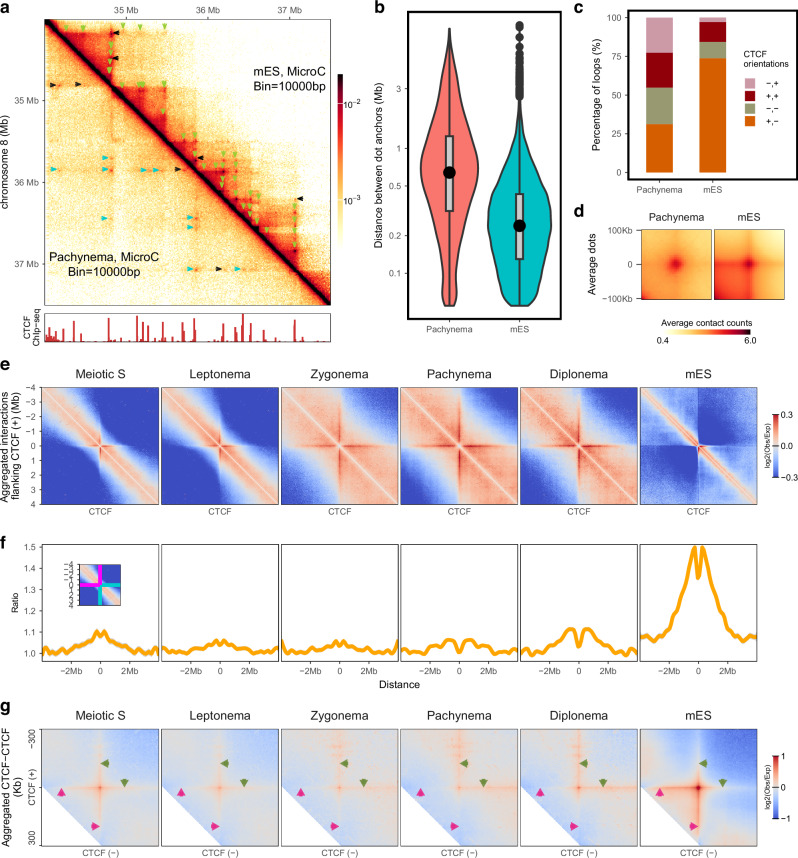
Meiotic specific interaction patterns shaped by CTCF and cohesin during meiosis I prophase. **a** Micro-C matrix of chromosome 8 at a resolution of 10 Kb (region: 34–37.5 Mb). Upper half: mES, lower half: Pachynema. Both mES and pachytene Micro-C data were sequenced to a depth of ~3 billion. Arrows indicate the dots located by Chromosight^89^. Cyan horizontal arrows mark the locations of dots detected exclusively in pachynema, yellow-green vertical arrows mark dots detected exclusively in mES cells, and black horizontal arrows mark dots detected in both pachynema and mES cells. CTCF ChIP coverage is plotted at the bottom. **b** Median distance between anchors of identified dots in pachynema (n = 8403 dots) and mES cells (n = 19,460 dots). Dot calling was performed at both 5 Kb and 10 Kb resolution by chromosight^89^. Boundaries of the embedded box: Represent the lower (25th) and upper (75th) quartiles. Black dot in the embedded box: Indicates the median value. **c** Percentage of dot anchors with different orientated CTCFs. Only both anchors containing one CTCF motif are considered for this analysis. **d** Aggregation of identified dots. The aggregation was performed at a resolution of 10 Kb, with a 100 Kb flanking region. **e** Aggregation of interactions surrounding CTCF binding sites with unified motif orientation. The analysis was performed at a resolution of 10 Kb, with a 4 Mb flanking region. Only the interactions at loci with forward-oriented CTCF was shown. **f** Quantification of the orientation bias. The observed/expected values of each bin at the center of the stripes were extracted. The matrix is symmetric. Both cyan lines indicate the interactions between CTCF binding sites with the downstream loci; Both magenta lines indicate the interactions between CTCF binding sites with the upstream loci. The bias was calculated as the ratio between each value on the green line and the corresponding value on the red line. The profile was plotted symmetrically representing the symmetric stripes in the matrix. **g** Aggregation of convergent CTCF interactions at a range of 100–300 Kb. Pink arrow: endpoint of stripes representing interactions between CTCF sites and intervening sequences during meiotic S phase. Green arrow: endpoint of stripes representing interactions between CTCF sites and sequences flanking the loop during meiotic S-phase. Note: The appearance of dots above the green arrows is an artifact from overrepresented problematic regions.

Despite clear observation of loop structures by electron microscopy, Hi-C from mammalian prophase I cells did not detect TADs or loops located at any defined loci^9,10,13–19^ (Supplementary table 2 and Supplementary Fig. 9a). Leveraging our deeply sequenced Micro-C data and finely defined stages across meiosis I prophase, we re-examined this question. As shown above and consistent with previous studies, we observed a progressive reduction in signals of interaction within TADs throughout meiosis I prophase^13–19^ (Fig. 2c and Supplementary table 2). In addition, no triangular signals indicative of interactions within TADs were discernible, even in the pachytene Micro-C interaction matrix built from 3 billion contacts (Fig. 4a (bottom left)). However, unlike in previous studies, a punctate grid-like pattern was observed throughout meiosis I prophase (Fig. 4a, and Supplementary Fig. 9a–c). These intense point-to-point interactions, which reflect higher frequencies of interaction between two specific genomic loci, have been termed “Hi-C loops” to distinguish them from random looping^37^.

In interphase somatic cells, “Hi-C loops” are typically detected as “corner dots” on top of the triangular signal indicative of TADs^26,48,49^. The decoupling of these two signals in meiosis, specifically the presence of “corner dots” without a triangular TAD signal, suggests that meiotic “Hi-C loops” may have properties that differ from those seen in somatic cells. To test this suggestion, we first compared Micro-C data from pachynema with publicly available Micro-C data from mouse embryonic stem (mES) cells with a similar sequencing depth (3.3 billion contacts)^24^. We identified ~10,000 “Hi-C loops” in pachytene cells (see “Methods”), roughly half of the number identified in mES cells (Fig. 4a and Supplementary Fig. 10b). Approximately 80% of the “Hi-C loops” featured at least one CTCF-defined anchor, similar to “Hi-C loops” detected in mES cells (91%) (Supplementary Fig. 10a, c). We found three significant differences between the “Hi-C loops” detected in pachynema and those observed in mES cells. First, the median distance between the two loop anchors in pachynema was nearly three times larger than that in mES cells (620 kb vs 235 kb) (Fig. 4b). Second, the “Hi-C loop” anchors in pachynema contained CTCF sites in any orientation, whereas nearly 74% of “Hi-C” loops in mES were anchored by convergent CTCF sites (Fig. 4c). Third, the aggregated “Hi-C loops” from pachytene cells were “fuzzier” than those from mES cells (Fig. 4d), suggesting that the increased interactions extend further from CTCF sites in pachytene cells than in mES cells.

A conserved trend of high proportion of “Hi-C loops” anchored by non-convergent CTCF sites is observed across all meiotic stages. However, this comparison is subject to the caveat that loop calling relies heavily on sequencing depth and the complexity of the library (Supplementary Fig. 10b–e). The progressive increase in inter-anchor distance observed closely matched the trend of increasing loop size in meiosis I prophase (Supplementary Figs. 5a, b, and 10d). We then aggregated the interactions at all pair-wise CTCF sites in different orientations (Supplementary Figs. 11a and 12), which allows for genome-wide quantification independently of loop calling^50,51^. Orientation bias of CTCF binding sites in “Hi-C loops” begins to diminish at the meiotic S-stage and becomes increasingly reduced as cells progress to pachynema.

**Fig. 5:**
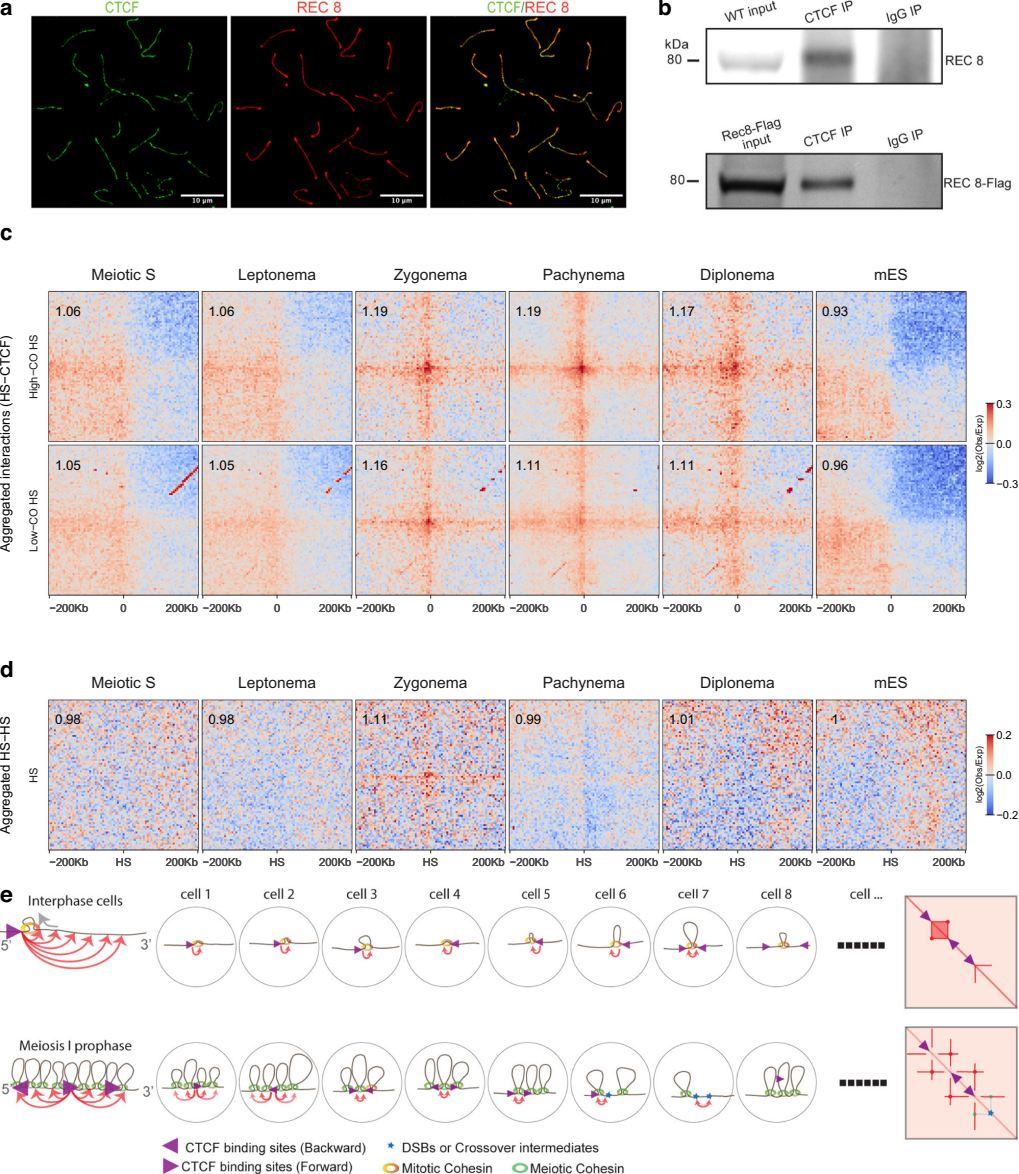
CTCF plays a role in anchoring cohesin complexes and meiotic loops. **a** A representative immunostaining image from two separate experiments demonstrates that CTCF and REC8 are localized along the axial elements during the pachytene stage. **b** Co-IP. CTCF was immunoprecipitated from wildtype (WT) mice or FLAG-tagged REC8 mice. Immunoblotting was performed with REC8 antibody for WT mice and FLAG antibody for REC8-FLAG mice. Normal rabbit IgG was used as a negative control. Each experiment was repeated three times. **c** Aggregated interactions between DSB hotspots and CTCF bound loci derived from Micro-C contact maps. DSB hotspots were derived from^93^. Hotspots with TSS or CTCF binding sites within ±5 Kb were excluded from the analysis. The strongest 1000 hotspots from CASTXB6 mice were selected and centered on PRDM9 motifs; A CO resolution probability was assigned to each hotspot based on COs identified by^94^. High-CO HS: 250 hotspots that are more frequently resulting in a CO. Low-CO HS: 250 hotspots that do not have a CO, but with higher DMC1 signal^93^. CTCF binding sites that coincided with the anchors of dots identified from pachytene Micro-C data were used for the aggregation analysis. Enrichment was calculated as the mean of the central 3 × 3 pixels and shown in the top-left corner. **d** Aggregated interactions between DSB hotspots derived from Micro-C contact maps. Hotspots were derived from^93^. The strongest 1000 hotspots from CASTXB6 mice were selected and centered on PRDM9 motifs; hotspots with TSS or CTCF binding sites within ±5 Kb were excluded from the analysis. Enrichment was calculated as the mean of the central 3 × 3 pixels and shown in the top-left corner. **e** A schematic illustrates how multiple closely aligned loops and CTCF shape the meiotic specific interaction patterns. The top panel shows representative interaction scenarios in individual interphase cells.

This bias in CTCF binding site orientation, towards convergent sites, is a “Hi-C loop” feature indicative of loop extrusion^48^. The large reduction in orientation bias observed in meiotic cells suggests that a different mechanism underlies CTCF-mediated interactions during meiosis. To explore this further, we aggregated contacts surrounding CTCF sites in the same orientation. A stripe pattern was observed in all cell types (Fig. 4e); as expected, contacts in the “wrong” direction increased as cells progressed toward pachynema, consistent with a reduction in orientation bias (Fig. 4e, f). Furthermore, enriched interactions could extend to several mega-bases, significantly greater than the distance in mES cells (Fig. 4e). These long-distance, orientation-independent interactions are consistent with a recently proposed “multi-loop” model, where a similar “stripe” pattern results from closely-positioned loops created by high cohesin density^52^; in this configuration CTCF-bound anchor sites should be able to contact nearby loops in both directions (Fig. 5e and Supplementary Fig. 11b). Closely positioned loops are rare in interphase cells, but it is the stereotypical chromatin organization during meiosis (Fig. 5e).

This direction-independence of anchor interactions is further illustrated by aggregating interaction matrices at pair-wise CTCF sites that are less than 300 Kb apart (Fig. 4G). In mES cells, “stripes” that reflect CTCF-loop interactions are consistent with increased interactions between CTCF-bound sites and sequences between the two CTCF sites (Fig. 4g and Supplementary Fig. 12; See Supplementary Fig. 13 for a schematic illustrating how patterns vary based on the distance between CTCF sites)^53–56^. This pattern changes as cells progress into meiosis I prophase; the interactions between the CTCF sites and sequences between them is reduced, while interactions between CTCF sites and sequences flanking the loop increase. This has been proposed to indicate a lack of de-novo loop formation and more extension of pre-established loops in situations where cohesin is more stable^46,51^. Consistently, a reduced enrichment at short distances was observed when aggregating interaction surrounding CTCF sites (Supplementary Fig. 14). A lack of short-distance loop formation is likely a key factor contributing to the disappearance of TADs during meiosis.

Our data show that during meiosis I prophase, CTCF contributes to defining the loop positions without constraining interactions within TADs. Moreover, CTCF bound loci form long-range contacts bidirectionally. These findings support a model where the stable meiotic loop array forms by loop association and further extension and that CTCF plays a role in organising meiotic chromatin.

### Stable CTCF/cohesin complexes are at the base of the meiotic loop array and interact with DSB hotspots at a high frequency

We found reproducible positioning of loops in our meiotic prophase data, suggesting that the interaction between CTCF and cohesins^57^ could establish an anchor point for the meiotic loop formation (Fig. 5e). REC8 and RAD21L localize to the meiotic chromosome axis in meiosis I prophase^41^. Colocalization of CTCF with the cohesin on the axes would support the notion that loci bound by CTCF are located at the proteinaceous base of the loop arrays at a high probability. A report analysing a CTCF conditional deletion in meiosis showed a weak CTCF signal on the axes on pachytene spreads that disappeared in the KO cells^58^. We revisited this question by performing instant Structured Illumination Microscopy (iSIM). We immunostained for CTCF using different antibodies and confirmed that CTFC is prominently bound to the axes as the synaptonemal complex assembles, implicating CTCF in the organization of the meiotic loop array (Fig. 5a). Furthermore, we determined that REC8 and CTCF physically interact using co-immunoprecipitation (co-IP) experiments. CTCF IP successfully pulled down REC8 in both wild-type and REC8-flag mice (Fig. 5b). However, RAD21L was not detected among the pulled-down proteins. RAD21L expression is limited to a short window during prophase I^41,59^, while REC8 is highly expressed throughout. We cannot rule out that CTCF interacts with RAD21L but it was not detected in our Western blots.

One of the prominent features of meiosis I prophase is the formation of programmed double strand breaks (DSBs) to initiate meiotic recombination. These occur at preferred regions of the genome, known as hotspots. Meiotic DSB repair occurs in the context of the synaptonemal complex and axial proteins have been shown to be present at DSBs^60^. CTCF has been shown to interact with PRDM9 and to be enriched at DSB hotspots, leading to the proposal that recombination hotspots interact with CTCF binding sites^60,61^. Accordingly, we found pair-wise contacts between frequently used DSB hotspots and CTCF sites found at loop anchors (Fig. 5c and Supplementary Fig. 15a). The signal was absent in a similar analysis using DSB hotspots preferentially used in a different mouse strain (Supplementary Fig. 15c). Furthermore, hotspots classified by their crossover (CO) resolution probability showed distinct interaction profiles. While both categories showed comparable interaction frequency in zygonema, the interactions in pachynema and diplonema were only detected at hotspots with high CO-resolution potential (Fig. 5c and Supplementary Fig. 15a). This indicates that these interactions are strongly associated with the tethering of recombination hotspots to the loop axis. Interestingly, we observed a weak but higher than expected interaction signal between hotspots only at zygonema (Fig. 5d and Supplementary Fig. 15b). Intriguingly, recombination hotspots have been shown to harbor insertions from distant sites^62^, and the proximity of DSBs would favor the chances of these events as well as translocations.

Our data partially answers a long-standing puzzle: Are there preferred sequences at the base of meiotic loop arrays in mammals? While in yeast the positioning of loops depends on the presence of REC8 and its DNA binding preferences^20^, our data suggest that in mice, CTCF plays a role in stabilizing some of these cohesion complexes.

### The interactions between regulatory elements are well preserved during meiosis I prophase

Meiotic chromatin faces the challenge of maintaining a balance between gene expression and a highly structured genome. We used Micro-C to examine how the reorganization of meiotic chromatin influences interactions between regulatory elements

**Fig. 6:**
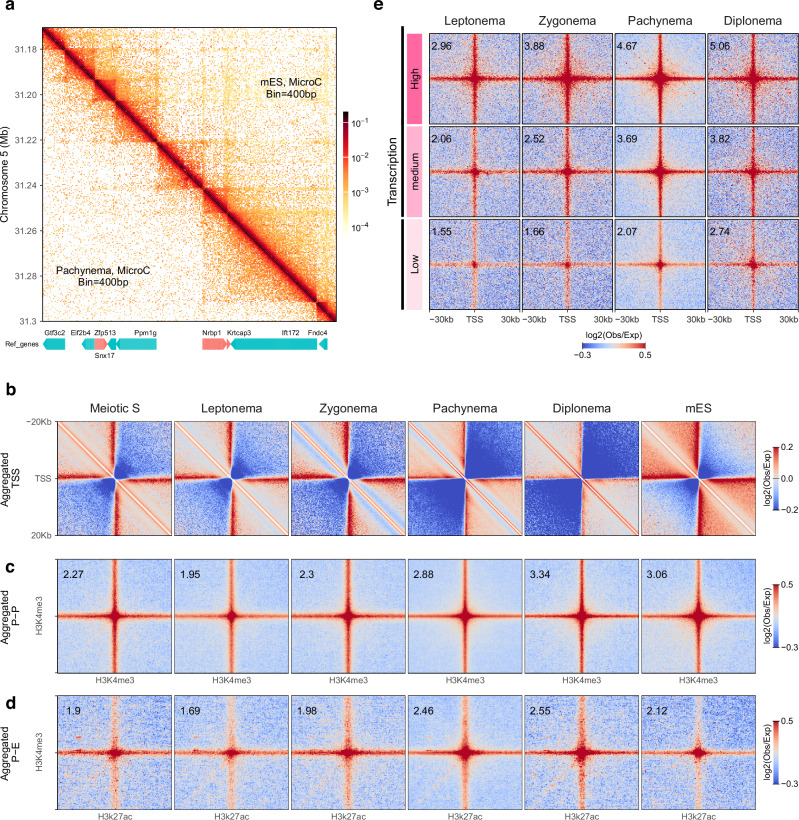
Micro-C revealed the preservation of fine-scale chromatin structure during meiosis I prophase. **a** A snapshot of a Micro-C matrix of chromosome 5 at a resolution of 400 bp (region: 31–31.4 Mb). Upper half: mES, lower half: Pachynema. Genes in the same region were annotated at the bottom. The arrows on the bars indicate the orientation of the genes. **b** Aggregated interactions centered around TSSs. TSSs were derived from USCS annotated genes. The aggregated interactions were calculated at a resolution of 200 bp with a flanking region of 20 Kb. **c** Aggregated promoter-promoter interactions. The promoters were derived from H3K4me3 Chip-seq data^25^. The H3K4me3 modification sites that overlapped with hotspots were excluded from calculations. Resolution was 400 bp with a flanking region of 30 Kb. Enrichment was calculated as the mean of the central 3 × 3 pixels and shown in the top-left corner. **d** Aggregated promoter-enhancer interactions. The enhancers were derived from H3K27ac Chip-seq data^25,85^. Common peaks from both studies were selected and only the sites that did not overlap with H3K4me3 peaks and hotspots were used for the calculation. Resolution was 400 bp with a flanking region of 30 Kb. Enrichment was calculated as the mean of the central 3 × 3 pixels and shown in the top-left corner. **e** The correlation between gene expression and P-P interactions. The genomic loci with H3K4me3 modification were divided into three groups based on their expression deduced from RNA seq data^64^. High: Represents the top third in terms of expression levels across all genes; Mid: Corresponds to the middle third in expression levels among all genes; Low: Encompasses the remaining genes, covering the lowest third in expression levels. The aggregated interactions were calculated at a resolution of 400 bp with a flanking region of 30 Kb. Enrichment was calculated as the mean of the central 3 × 3 pixels and shown in the top-left corner.

We first built Micro-C matrices at 400 bp resolution, which enabled observation of interaction domains at the scale of single gene units (Fig. 6a and Supplementary Fig. 16). We found distinct interaction domains involving one or a few genes and intergenic regions, as were previously observed in mES cells^24^ (Fig. 6a (top right) and Supplementary Fig. 16). Visual inspection of our Micro-C maps hinted at high frequencies of interactions between transcription start sites (TSSs) and their corresponding gene bodies, shown as stripes, as well as enriched interactions between TSSs throughout meiosis I prophase (Fig. 6a and Supplementary Fig. 16). However, these signals were faint, preventing definitive conclusions at the level of individual loci (Fig. 6a (bottom left) and Supplementary Fig. 16). To quantify this, we use aggregation analysis of the interactions flanking TSSs at a resolution of 200 bp. Stripe signals around TSSs persisted throughout all examined stages, with intensities comparable to that of mES cells (Fig. 6b). Interestingly, we found that the insulation between genes was increased at pachynema and diplonema (Fig. 6b, and Supplementary Fig. 17a, b).

To further examine interactions between regulatory elements, we aggregated pair-wise promoter-promoter (P-P; H3K4me3-H3K4me3) and promoter-enhancer (P-E; H3K4me3-H3K27ac) interactions. We observed clear P-P and P-E interactions at all stages, with a comparable intensity to that has been found in mES cells (Fig. 6c, d and Supplementary Fig. 18). These interactions were not detected in Hi-C data (Supplementary Fig. 19a, b). Furthermore, in line with observations from interphase cells^63^, we found that the P-P and P-E interactions could occur between two loci that are extremely distant from one another (Supplementary Fig. 20). Unlike in interphase cells, there was a marked decrease in interactions beyond 30 Mb from zygonema to diplonema (Supplementary Fig. 20a, b), and interactions between regulatory elements on different chromosomes were barely visible after zygonema (Supplementary Fig. 21). To further understand the regulatory role of these interactions, we aggregated promoter interactions based on expression levels throughout prophase I^64^. The strongest P-P interactions were associated with the highest mRNA levels, indicating that these interactions may play a regulatory role.

**Fig. 7:**
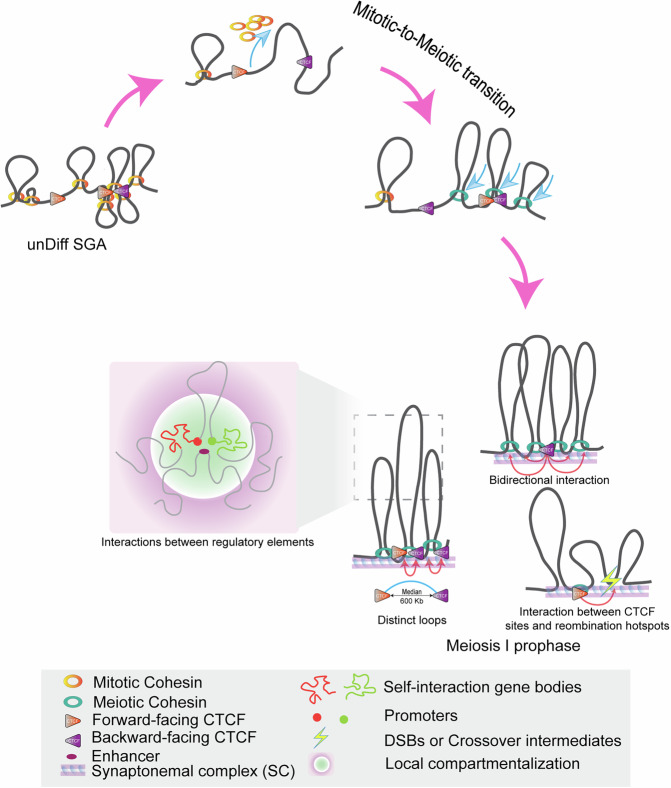
A schematic of the proposed model for chromatin folding changes from undifferentiated spermatogonia to meiosis I prophase. The diagram illustrates changes in chromatin folding and the transition from mitotic to meiotic cohesin prior to meiotic entry, as well as interactions between CTCF-anchored loop bases and surrounding axis-associated elements, and interactions among transcriptional regulatory elements within the local A compartment.

In conclusion, our data indicated that, despite the meiotic chromatin being highly structured by the formation of loop arrays, fundamental interactions between regulatory elements are well preserved (Fig. 7).

## Discussion

For proper chromosomal separation, meiotic chromosomes undergo compression through loop array formation^8,65,66^. While cytological studies have defined this loop-axis array configuration, only the recent advances of Conformation Capture (3C)-based methods allow us to resolve the molecular features genome-wide. Here, by examining the genomic interactions at the highest temporal and spatial resolution to date, we gained insights into the principles of chromosomal organization during mammalian meiosis.

We show that long-range whole-genome compartmentalization is lost consistent with the formation of an axis-loop array. Nonetheless, local compartments are preserved. Previous studies identified refined local compartments, but they were not observed in the corresponding regions of interphase cells^13,16,18,19^, leading to the conclusion that they were unique features of meiotic chromosomes. By examining deep-sequenced Hi-C matrices from stages preceding meiosis, we found that the local “checker-board”-like pattern was also present in germline interphase cells, although with varying interaction strengths. Importantly, a recent study revealed that the median size of compartmental intervals in somatic interphase cells is much smaller than it was assumed, with a median size of 12.5 Kb^33^. We propose that the local compartments are more visible in meiosis due to the reduction in “noise” originating from high dynamics of loop formation and global compartmentalization. Overall, the similarity of compartment intervals between germline interphase and prophase cells point to a common underlying principle governing chromosome organization in these two cell types.

Our data revealed a real-world example in which transcription activity is modulated in the absence of TADs and genome-wide compartmentalization. During meiosis I prophase, transcription is transiently silenced at early stages and reactivated at pachynema^67,68^. Nevertheless, the interactions between regulatory elements, key factors in regulating gene transcription, are well-preserved (Fig. 7). This is in line with the observation that P-P/P-E interactions are resilient to acute loss of architectural factors, such as CTCF, cohesin, WAPL, YY1 and RNA polymerase II^69,70^. Two models were proposed regarding the establishment and maintenance of P-P/P-E interactions. In the time-buffering model, chromatin architectural factors, such as cohesins and CTCFs, are required for establishing these interactions, rather than maintaining them^69^. In the second model, these contacts between regulatory elements were proposed to be driven by micro-phase separation, resembling A/B compartmentalization^70^. The two models are not mutually exclusive. The P-P/P-E interactions during meiosis I prophase potentially follow the same rule. It is intriguing that P–P interactions remain associated with mRNA abundance even when active transcription is absent. According to the first model, this association could be established preceding meiotic entry. The “micro-phase separation” model emphasises the importance of epigenetic marks on regulatory elements in shaping their interactions. Indeed, H3k4me3, a mark for active promoter, persists throughout meiosis I prophase together with other histone marks and its enrichment positively correlates with mRNA levels^25^ and P-P interactions (Supplementary Fig. 22). How transcriptional regulator interactions are established/maintained and how they shape transcriptional programs during meiosis remain to be answered.

Our high temporal resolution allowed us to pinpoint the specific stage at which interphase chromatin structure begins to transition into meiotic chromatin. We isolated a rare cell population that left the spermatogonial mitotic cycles but had not yet entered meiosis as traditionally defined, e.g. the preleptotene stage where STRA8 and MEIOSIN are believed to direct the cell switch from mitosis to meiosis^71^. The chromosomes at these transition stages are mostly devoid of loops (Fig. 7). Interestingly, this folding intermediate was only previously observed when somatic cells exit mitosis^40^. During mitosis exit condensins are replaced by cohesin. Here, the chromatin reorganization coincides with a decrease in protein levels of the mitotic cohesin and a concomitant increase in the meiotic cohesin complexes. As cells progress into meiosis, an increased number of extended loops are formed, which coincide with the increase in REC8 (Fig. 7). Interestingly, Rec8 expression is activated by retinoic acid (RA), the extrinsic signal for meiotic initiation, independently of STRA8^72^. This led to the proposal that there are two pathways activated by RA to initiate meiosis. Our data positions REC8 as the first responder, preceding the STRA8-dependent transcriptional boost of meiotic genes. Meiotic chromatin establishment appears to be a two-stage process. First, TADs weaken as cells differentiate into spermatogonia. Second, at the transition stage, mitotic cohesins are removed from chromosomes prior to the loading of meiotic cohesins. The cohesin exchange accompanied by the establishment of a folding intermediate resembles the observation in somatic cells exit mitosis^40^. Whether this intermediate folding state is conserved when switching between SMC complexes (condensin to cohesin or mitotic to meiotic cohesin) needs to be further examined.

The programmed DSBs are formed and repaired in the context of loop-axis arrays. Defining genomic sequences anchoring to the axis is of great interest in understanding the loop-array structure. Repeat elements, including LINE, SINE, and LTR sequences, are highly enriched along the meiotic axis^73^. Recently, imaging together with ChIP-seq also suggest that CTCF is axis-associated^17,58,60^. Nonetheless, chromatin loops were undetectable in Hi-C data^9^. We combined Micro-C experiments with high sequencing depth to examine this question. We found a clear pattern in meiosis I prophase consistent with well positioned loops. These “dots” are reminiscent of the punctate grid-like interactions described in yeast that are mediated by the meiotic cohesin subunit, REC8^20,21^. We found here that they coincide with CTCF sites. Importantly, and distinct from somatic interphase cells, they do not show increased interactions in the intervening regions, suggesting that they are not the typical TAD corner signal. Furthermore, convergent CTCF motifs are not predominantly found at these sites. Similar to somatic interphase cells^42,43^, CTCF-defined loops are rare, with an estimated probability of 1.7% for the strongest detected loop in pachynema (The estimation was performed with a caveat that the parameters were derived from interphase mES cells^74^). We show that CTCF colocalizes with REC8 at the chromosome axes supporting the notion that the dots we identified are at the base of the bonafide meiotic loops. Furthermore, CTCF interacts with REC8 in the testes, but not with RAD21L. This distinction would suggest that REC8 is the major meiotic-specific kleisin involved in the formation and maintenance of the meiotic loop array. This finding is in line with the defining role that REC8 plays in the modified sister chromatid cohesion that serves as the foundation for proper meiotic chromosome segregation.

We also cannot distinguish whether all loops have CTCF at their bases, but there is a clear preference for this configuration. We found that up to 20% of “dots” in pachymena do not have a CTCF binding site at their anchors. The densely loaded cohesins may act as anchors independently of CTCF in some cases. Furthermore, we observed that unlike in interphase cells, dots look “fuzzier” meaning that there are interactions beyond the precise CTCF binding site, maybe reflecting the more dynamic and/or flexible nature of these interactions that don’t require convergent CTCF sites.

In interphase cells, the association and dissociation of cohesins is transient, and emerging live-cell microscopy data has found that loops are both rare and short-lived^42,43^. During meiosis I prophase, the loop array remains in place for days but how they form and “grow” is unclear. Coalescence of pre-formed loops was first proposed as a model for establishing axial loop arrays^75^. In recent years, loop extrusion appeared as an alternative mechanism, which has been suggested as a key player for organising chromosomes during meiosis in Saccharomyces cerevisiae^20^. In Caenorhabditis elegans, meiotic specific cohesins COH-3/4 dynamically associate with chromosomes and likely regulate higher-order chromosome structure through loop extrusion^76^. Our data cannot formally measure the dynamics of loop formation. We favor the hypothesis that meiotic loops form via association and further loop fusion (as in^75^) rather than “extrusively”. The reasons are as follows: First, the high dynamics of cohesin association and dissociation, together with loop extrusion, lead to enriched interactions within TADs when CTCF is present on chromosomes. The detection of “dots” in the absence of TADs argues against active loop extrusion. Second, actively extruding loops are stalled by CTCF on one side, resulting in orientation bias. Such bias is substantially reduced throughout meiosis I prophase. As discussed previously, Hi-C/Micro-C data are not suitable for assessing dynamics. It remains possible that minimal loop extrusion activity is sustained throughout meiosis I prophase. Accurate assessment of loop-formation dynamics and cohesin turnover will likely require live-cell imaging in future studies. The cohesin regulators, WAPL and NIPBL are expressed through mammalian meiosis, and experiments perturbing this regulation would inform how the stability of cohesins affects the meiotic structure and function.

Determining loop size is a key issue in understanding the loop-axis array configuration. Loop sizes inferred from our Hi-C data increase progressively throughout meiotic prophase I, in general agreement with previous studies^15,18^, with the notable exception of diplonema. Our data indicates a decrease in diplotene loop size. It is noteworthy that the curve of the *P(s)* derivative does not exhibit a typical “bump” across the genomic distance range associated with the expected loop size (Supplementary Fig. 5b, d). One of the possibilities is that diplonema is reorganising their chromatin structure, leading to a heterogeneity in average loop size. A disagreement between loop size derived from Hi-C and cytological analysis has been previously highlighted^9^. The stability of meiotic cohesin complexes may contribute to discrepancies between loop sizes inferred from Hi-C data and those observed in cytological studies. Estimates of average loop size from Hi-C data are typically based on simulations of loop extrusion dynamics in somatic cells^37,77^. These simulations assume rapid turnover of loop extrusion factors (LEFs) and continuous loop extrusion. These assumptions might not accurately reflect the properties of meiotic cohesins. Simulation tailored to meiotic Hi-C data might be necessary to better understand the nature of meiotic chromatin folding, such as loop size. Indeed, if we calculate loop sizes as the median distance between anchor points systematically identified from the Micro-C maps, the loop size in pachynema would be ~600 kb, in line with cytological observations^9,78^. Furthermore, based on the measured synaptonemal complex length in pachynema (215 micrometers)^13^, this loop size would translate to ~ 20 loops per micron, a number that has been deemed to be conserved between organisms^78^ (genome size = 2.7 Gb / (600 kb/loop) = 4500 loops; 4500 loops/215 micrometers = 21 loops per micron).

All the Hi-C and MIcro-C maps were derived from hybrid mice because of the potential to detect inter-homolog interactions. We found that the proportion of inter-homolog interactions is very low, ranging from 0.3% to 0.6%, even for the Micro-C dataset. The datasets and techniques used here are limited for making inferences about coalignment particularly at short separations. Future experiments with mice lacking interhomolog pairing could provide a true estimate of background signal to make accurate observations regarding interhomolog paring.

The linear loop array is observed in both mitotic and meiotic prophase with a conserved morphology. However, the molecular features of the loop array in mitotic prophase are less well explored, potentially due to its transient nature. Our observations from the prolonged meiosis I prophase may therefore provide insights into features shared with mitosis. Given the comparable chromatin length in mitotic and meiotic prophase^79^, we speculate that the loop sizes observed in this study may reflect those present during mitotic prophase, which have been shown to vary substantially across species^80^.

## Methods

### Mouse strains

All experiments were conducted in compliance with NIH Animal Care and Use regulations. C57BL/6J (Stock no. 000664) and CAST/EiJ (Stock no. 000928) were purchased from The Jackson Laboratory. The F1 hybrids were in-house bred with male CAST/EiJ with female C57BL/6J. Mice were maintained in a controlled environment with a 12-h light/dark cycle, temperature of 22 ± 2 °C, and 50%–60% humidity. The animal protocol is approved by NIDDK ACUC.

To generate Flag-tagged Rec8 mice, a single guide RNA (sgRNA) targeting the C-terminal end of the Rec8 gene, a single-stranded DNA donor template, and purified Cas9 protein were microinjected into C57BL/6J zygotes. The resulting embryos were transferred into pseudo-pregnant female mice. Founder mice were identified through PCR genotyping and confirmed by Sanger sequencing.

### Antibodies used

The following antibodies were used for immunofluorescence microscopy or flow cytometry: anti-PLZF Santa Cruz (sc-28319), anti-DMRT1, Santa Cruz (sc-377167), anti-SYCP3, Santa Cruz (sc-74569), anti-STRA8, Abcam (ab49602), anti-H1T (a custom-made antibody), anti-SCP1 Novus Biologicals (NB300-229B), anti-Rec8 (a custom-made antibody), anti-Rec8 (ab192241), anti-Rad21L (a gift from Miguel Brieño-Enriquez), anti-Rad21, Abcam (ab217678), anti-CTCF, Abcam (ab128873). The following antibodies were used for Western blot and co-immunoprecipitation (Co-IP) assays: anti-Rec8, Abcam (ab192241), anti-Rad21, Abcam (ab217678). The following antibodies were used for ChIP: anti-CTCF, Abcam (ab70303). Normal rabbit IgG. Upstate (12-370). Anti-TATA binding protein (TBP) (ab63766). Anti-FLAG: Millipore-Sigma (F1804).

### Nuclei preparation and isolation by FACS

Nuclei were prepared and sorted as described before^25^. Briefly, mice were euthanized, and testes were retrieved. For Hi-C, the testes were fixed with 1% formaldehyde at room temperature for 10 min. For Micro-C, the testes were fixed with 1% formaldehyde at room temperature for 10 min followed by 3 mM disuccinimidyl glutarate (DSG) for an additional 45 min at room temperature. Fixation was quenched with glycine (final concentration 125 mM). The fixed testes were homogenized with at least 10 strokes in a Dounce homogenizer and filtered by passing through a 70 μm cell strainer. The cell suspension was washed once with 1× PBS (phosphate buffered saline) and then resuspended in nucleus extraction buffer (15 mM Tris-HCl pH 7.4, 0.34 M sucrose, 15 mM NaCl, 60 mM KCl, 0.2 mM EDTA (Ethylenediaminetetraacetic acid), 0.2 mM EGTA (ethylene glycol tetraacetic acid). Nuclei were extracted using a Dounce homogenizer with 30 strokes of tight pestle, repeated once. Nuclei were filtered through a 40 μm cell strainer and resuspended in a chilled PBTB buffer (1× PBS with 0.1% Triton X-100, 5% bovine serum albumin, and protease inhibitor). The nuclei were incubated with primary antibodies at room temperature for 1 h or 4 °C overnight. They were then washed with PBTB and labeled with secondary antibodies at room temperature for 30 min. After washing twice with PBTB, nuclei were filtered with a 40 μm cell strainer and stained with DAPI (4′,6-diamidino-2-phenylindole) for sorting. Singlets were gated by FSC and FAC. 2C, 2C-4C, and 4C populations were first separated by DAPI signal. Then, targeted populations were isolated by the combination of different markers. The sorted nuclei were collected in PBTB. The collected nuclei were either immediately processed for downstream experiments or stored at −80 °C.

### Micro-C

Micro-C was performed as described^24^ with minor modifications. Briefly, 0.5 million sorted nuclei were resuspended with 100 μL MBuffer#1 (50 mM NaCl, 10 mM Tris-HCl pH 7.5, 5 mM MgCl2, 1 mM CaCl2, 0.2% NP-40, 1× protease inhibitor cocktail (Roche, 04693132001) and incubated on ice for 20 min. Nuclei were collected by centrifugation (900 g, 5 min) and resuspended with 100 μL MBuffer#1, and digested with 2.5 U MNase (Worthington Biochem #LS004798) at 37 °C for 10 min. The reaction was stopped by adding 2 μL 200 mM EGTA and incubating at 65 °C for 10 min. Nuclei were washed three times with 1xNEBbuffer2.1 (NEB, #B7202S) and resuspended with 45 μL 1xNEBbuffer2.1. Dephosphorylation of DNA ends was performed by adding 5 μl rSAP (NEB, #M0203) and incubating at 37 °C for 45 min. rSAP was inactivated by incubating at 65 °C for 5 min. The addition of 5′-phosphate was performed by adding end-repairing buffer (5 μl 10xNEBbuffer2.1, 3 μl 100 mM DTT (ThermoFisher, A39255), 2 μl 100 mM ATP (ThermoFisher, R0441), 2 μl T4 PNK (NEB, #M0201L), and 30 μl H2O) and incubating at 37 °C for 5 min. Then 8 μl 5 U/μL Klenow Fragment (NEB, M0210S) was added and 3′ overhangs were removed by incubating at 37 °C for 15 min. End repair and biotin labeling was performed by adding a mix of dNTP (25 μl 0.4 mM Biotin-dATP, 25 μl 0.4 mM Biotin-dCTP, 1 μl 10 mM dTTP, 1 μl 10 mM dGTP, 10 μl 10XT4 DNA Ligase Buffer and 1 μl 10 mg/mL BSA) and incubating at 25 °C for 45 min. The reaction was stopped by adding 12 μl 0.5 M EDTA and incubating at 65 °C for 20 min. Nuclei were washed with 1xNEB ligation buffer with 0.1% Triton X-100 and resuspended with ligation-mix (100 μl 10× T4 DNA Ligase Buffer, 5 μl 20 mg/mL BSA, 1 μl 20,000 U/mL T4 DNA Ligase, 100 μl 0.1% Triton X-100, 884 μl H2O). Proximity ligation was performed by incubating at room temperature for 3 h. Unligated biotin was removed by treating the nuclei with 200 U Exonuclease III (NEB, M0206S) at 37 °C for 5 min. Reverse crosslinking was performed by adding 25 μl proteinase K and 25 μl of 10% SDS, then incubating at 65 °C overnight. The DNA was purified by phenol/chloroform and fragments above 200 bp were selected by AMPure XP (BECKMAN COULTR, A63881). End repair and adaptor ligation was performed by KAPA HyperPrep Kit (Roche, 07962347001). Adapter ligated DNA was purified by AMPure XP and eluted in 100 μl EB buffer (Qiagen, 19086). Biotin-labeled DNA was captured by adding 10 μL Dynabeads™ M-280 Streptavidin (ThermoFisher, 11205D), pre-washed with 2× B&W Buffer (10 mM Tris-HCl pH 7.5, 1 mM EDTA, 2 M NaCl), and incubated for 15 minutes.Beads were then washed three times with 1xTB&W (5 mM Tris-HCl (pH 7.5), 0.5 mM EDTA, 1 M NaCl, 0.05% tween 20) and once with 10 mM Tris-Cl pH 8.0. Beads were resuspended with 20 μl of EB buffer. Final library amplification was performed using the KAPA HyperPrep Kit, and DNA sequencing was carried out on the Illumina HiSeq 2500 or HiSeq X.

### Hi-C

Hi-C experiments were performed as described by^81^ with some modifications. Sorted nuclei were resuspended in digestion buffer (44 μl of 10× NEB DpnII buffer, 38 μl of 1% SDS, and 300 μl H2O) and incubated at 65 °C for 10 min. SDS was quenched by adding 44 μl 10% Triton X-100 and incubating at 37 °C for 1 h. Chromatin digestion was performed with 400 U of DpnII at 37 °C overnight (12–16 h). 10 μl of the mixture was used for quality control of the digestion. Digested nuclei were washed once with 1× NEB buffer2 and resuspended with 400 µl premixed biotin fill-in buffer (1.5 μl 10 mM dCTP, 1.5 μl 10 mM dGTP, 1.5 μl 10 mM dTTP, 37.5 μl 0.4 mM biotin-14-dATP, 40 μl NEB buffer2, 10 μl 5U/μl DNA polymerase I Klenow, 308 µl H2O). Biotin fill-in was carried out at 37 °C for 1 h. Biotin labeled nuclei were washed once with 1× NEB ligation buffer and resuspended in 1 ml ligation mix (100 μl 10× NEB ligation buffer, 100 μl 10% Triton X-100, 10 μl 10 mg/ml BSA, 2 μl 2000 U/μl T4 DNA ligase, 788 μl H2O). Ligation was performed at room temperature for 4 h. Ligated nuclei were pelleted and resuspended in a reverse cross-link buffer (150 μl water, 5 μl 5 M NaCl, 5 μl 10 mg/ml Rnase). Crosslinks were reversed by adding 20 μl 10% SDS, 20 μl 20 mg/ml proteinase K and incubating at 65 °C overnight. DNA was purified by phenol:chloroform and resuspended with 50 μl of EB buffer (Qiagen). To remove the unligated biotin, a reaction mix was prepared for every 5 μg of DNA (5 ug DNA, 5 μl 10X NEBuffer 2.1, 0.125 µl 10 mM dATP, 0.125 µl 10 mM dGTP, 5 µl 3000 U/ml T4 DNA polymerase (NEB), bring to 50 μl with H2O). The reaction was performed by incubating at 12 °C for 2 h. DNA was purified by phenol:chloroform and resuspended with 150 μl of EB buffer (Qiagen). The DNA was sheared to a size range of 300–500 bp by sonication. DNA fragments with size ranging between 300 bp and 500 bp were selected by AMPure XP beads. Biotin-labeled DNA was captured by adding 10 μL Dynabeads™ M-280 Streptavidin (ThermoFisher, 11205D), pre-washed with 2× B&W Buffer (10 mM Tris-HCl pH 7.5, 1 mM EDTA, 2 M NaCl) and incubated for 15 min. Beads were then washed three times with 1xTB&W (5 mM Tris-HCl (pH 7.5), 0.5 mM EDTA, 1 M NaCl, 0.05% tween 20) and once with10 mM Tris-Cl pH 8.0. Beads were resuspended with 20 μl of EB buffer. Final library amplification was performed using the KAPA HyperPrep Kit, and DNA sequencing was carried out on the Illumina HiSeq 2500 or HiSeq X.

### ChIP-seq

Mice were euthanized and testes were retrieved. The testes were fixed with 1% formaldehyde at room temperature for 10 min and quenched with 125 mM glycine for 5 min. The fixed testes were homogenized with Dounce homogenizer and filtered by passing through a 70 μm cell strainer. The cell suspension was washed once with 1X PBS (phosphate buffered saline) and then resuspended with Lysis buffer 1 (0.25% Triton X-100, 10 mM EDTA, 0.5 mM EGTA, 10 mM Tris-HCl pH 8). Cells were collected by centrifugation at 900 g for 5 min and resuspended with Lysis buffer 2 (200 mM NaCl, 1 mM EDTA, 0.5 mM EGTA, 10 mM Tris-HCl pH 8). Cells were then collected by centrifugation at 900 g for 5 min and resuspended with RIPA buffer (10 mM Tris-HCl pH 8, 1 mM EDTA, 0.5 mM EGTA, 1% Triton X-100, 0.1% sodium deoxycholate, 0.1% SDS (sodium dodecyl sulfate), 140 mM NaCl plus protease inhibitor). Chromatin was sheared into fragments of approximately 50–300 bp using Bioruptor (Diagenode).

The immunoprecipitation was performed by adding 1–2 μg antibodies and incubating at 4 °C overnight. To pull down the immuno-complexes, 50 μl pre-washed Dynabeads Protein G (30 mg/ml, Novex) were added and incubated at 4 °C for 2 h. The beads were washed once with low salt buffer (0.1% SDS, 1% Triton-X-100, 2 mM EDTA, 20 mM Tris-HCl pH 8, 150 mM NaCl), once with high salt buffer (0.1% SDS, 1% Triton-X-100, 2 mM EDTA, 20 mM Tris-HCl pH 8, 500 mM NaCl), twice with LiCl buffer (0.25 M LiCl, 1% IGEPAL-CA630, 1% sodium deoxycholate, 1 mM EDTA, 10 mM Tris-HCl pH 8) and twice with TE buffer. The ChIPed DNA was purified following the protocol of IPure kit v2 (Diagenode). Libraries were prepared by KAPA HyperPrep Kit (Roche, 07962347001). DNA sequencing was performed on the Illumina HiSeq 2500 or HiSeq X.

### Hi-C/Micro-C data processing

Both Hi-C and Micro-C data were processed following the 4DN Hi-C data processing pipeline. Briefly, the pair-end sequenced reads were mapped to mm10 using BWA (version 0.7). The pairs were then filtered by Pairtools (version v0.3.0) to generate valid pairs. Matrices at different resolutions were built and balanced by the Cooler package^82^ (version 0.8.11). The reproducibility of Hi-C and Micro-C data were examined by Hicrep (version 0.2.6).

### ChIP-seq analysis

Sequenced reads were mapped to the mouse mm10 reference genome with BWA-MEM^83^ (version 0.7). Peaks for CTCF and H3K27ac ChIP-seq were called using MACS2 (version 2.2.7.1)^84^ with default parameters. Peaks for H3K4me3 in meiosis prophase I sub-stages were previously called^25^ and merged in this study. Two datasets of H3K27ac ChIP-seq were utilized. The first dataset, generated in our prior study, was performed on SCP3-positive, H1t-negative sorted nuclei^25^. The second dataset was obtained from Dr. Satoshi H. Namekawa’s group^85^ and performed on pachytene spermatocytes. Common peaks identified in both studies, excluding those overlapping with H3K4me3 peaks, were used as enhancers. CTCF motifs were called using FIMO from MEME suite (version 5.4.1)^86^ with JASPAR PWM MA0139. All parameters were set as default.

### Contact probability decaying curve and derivatives

The contact probability as a function of genomic distance *P(s)* curves were computed using cooltools.expected_cis from Cooltools (version 0.5.4^87^ on 1 Kb binned data. The curves were smoothed at log-scale on both y-axis and x-axis. Given the special configuration of chrX and chrY during meiosis I prophase, only autosomal chromosomes were considered in this calculation. The derivatives were calculated at log-log scale from the smoothed *P(s)* curves.

### Insulation and TAD analysis

We calculated insulation score^36^ at 10 Kb resolution with sliding windows of 100 Kb by Cooltools^87^ (version 0.5.4). To call the boundaries, a threshold was set by threshold = “Li”, and regions with stronger insulation than the threshold were identified as boundaries. TAD domains were called as regions between two adjacent boundaries.

To measure the average insulation between TADs, we first identified conserved TAD boundaries by overlapping the TAD boundaries (±10 Kb expansion) from unDiff.SGA and mES (Hi-C data from^88^. The conserved TADs are provided as a Source Data file. The average insulation score of the conserved TAD boundaries and the flanking regions (±150 Kb from TAD boundaries) was calculated. To enable a standardized comparison of insulation scores across the analyzed stages, the values were normalised by assigning “zero” to the TAD boundary. The insulation scores and conserved TAD boundaries are provided as a Source Data file.

To reveal the dynamics of TADs, we identified the conserved TAD domains by selecting the domains called from unDiff.SGA and defined by conserved boundaries. The aggregated computation was performed by using Coolpuppy (version 1.0.0^50^ and plotted in R.

### Hi-C loop calling and loop size calculation

The Hi-C loops are called by Chromosight^89^ (version 1.6.3) at resolutions of 5 Kb and 10 Kb with the following parameters: --min-dist 50000 --max-dist 10000000 --min-separation 10000 --pearson=0.4. Loop called from both resolutions were merged with a minimum distance of 5 kb. For loop size calculation, we excluded loops with anchor distances below 50 kb and if neither anchor overlap with CTCF peaks. Loops identified by Chromosight are provided as a Source Data file.

### Compartment analysis

PCA based method and Calder^34^ were both used to identify compartment domains. Eigenvector decomposition was performed at a resolution of 100 Kb using eign-cis from Cooltools (version 0.5.4^87^. GC content was used to orient the first eigenvector.

Compartments calculated by Calder were performed at 50 Kb resolution with default parameters. The compartment calculations were carried out by the Calder algorithm at a 50 Kb resolution using default settings. The compartmental ranking called from Calder and Eignvector 1 are provided as a Source Data file.

### Aggregated analysis

All aggregated interactions in this study were calculated using Coolpuppy (version 1.0.0^50^ and plotted in R. To aggregate the interactions between CTCFs, we selected the CTCF binding sites that are both at the top quartile of peak strength and the quartile with the lowest p-values in motif calling. The selected CTCF binding sites are provided as a Source Data file. The motifs with different orientations were aggregated separately. If not specified otherwise, pair-wise CTCF binding sites positioned within a range of 100 Kb to 2 Mb were used for calculation. To measure the enrichment at different stages, we calculated the average of the three central pixels. The aggregation of P-P and P-E interactions were conducted at sites positioned within a range of 5 Kb to 5 Mb, if not specified. The TSS, promoter (H3K4me3 peaks) and enhancer (H3K27ac peaks) used for this anaysis are provided as a Source Data file. The pair-wise CTCF binding sites and DSB hotspot sites are provided as a Source Data file.

### Western blot and co-immunoprecipitation (Co-IP) assays

For Western blot analysis, sorted nuclei were lysed by incubating with RIPA buffer (Pierce, 89900) and 1X Protease Inhibitor Cocktail (Roche 04693132001) on ice for 10 min. Lysates were mixed with NuPAGE LDS sample buffers and incubated first at 65 °C overnight, then at 95 °C for 15 min to reverse crosslinking and denature the proteins. Proteins from 50,000 to 100,000 nuclei were loaded onto 4%–12% Bis-Tris SDS-PAGE gel and transferred to PVDF (Invitrogen LC2002) or nitrocellulose (Invitrogen LC2001) membranes. The transferred membranes were blocked with Intercept Blocking Buffer (LICORbio) for 1 h at room temperature and blotted overnight at 4 °C with primary antibodies (diluted at 1: 1000 ~ 2500) in Intercept Blocking Buffer with 0.1% tween-20. After washing, secondary antibodies conjugated with Alexa Fluor 700 were diluted at 1:5000 in Intercept Blocking Buffer with 0.1% tween-20 and 0.1% SDS and incubated at room temperature for 1 h. The membrane was scanned with Amersham Typhoon (software version 3.0.0.2). The uncropped scans of all replicates are provided as a Source Data file.

For Co-IP assays, nuclei extraction was adapted from^90^. Briefly, testes were dissected and tunicae were removed. The testes were then moved to tissue homogenizer (Ace Glass, 8325-18) and homogenized in nuclei extraction buffer (10 mM Tris pH 7.4, 5 mM MgCl2, 10 mM NaCl, 1X Protease Inhibitor Cocktail) until no tubules were visible. Cell suspension was filtered through a 100 um cell strainer and incubated on ice for 10 min. After adding 0.2% Triton X-100, nuclei were inverted for six to ten times and then centrifuged at 1200 g for 5 min. Nuclei were resuspended in Pierce IP buffer with 1X Protease Inhibitor Cocktail, 1.5 mM MgCl2 and 100 U/ml Benzonase, and incubated at 4 °C for 2–4 h with rotation. Lysates were centrifuged at 17,000 g for 10 min. Supernatants were collected in a fresh tube and pre-cleared with protein-G beads (Invitrogen, 10003D) for 1 h. Immunoprecipitation was performed by incubating lysates with primary antibodies at 4 °C for 2 h or overnight. After four times washing, proteins were eluted by boiling in NuPAGE LDS sample buffers at 95 °C for 15 min. Western blot was done as described above. The uncropped scans of all replicates are provided as a Source Data file.

### Instant Structured Illumination Microscopy (ISIM)

Meiotic chromosome spreads were prepared following the protocol described by^91^. Spreads were incubated with REC8 antibody (1:200 dilution in PBST: 1× PBS with 0.1% Triton X-100) for at least 1 h at room temperature. The spreads were washed three times with PBST and incubated with a goat anti-rabbit secondary antibody (1:500 dilution) for 1 h. The spreads were incubated with an AlexaFluor-488-conjugated anti-CTCF antibody (ab203704) for at least 1 h at room temperature. Imaging was performed using a custom-built microscope equipped with a VT-iSIM module (VisiTech International).

### Reporting summary

Further information on research design is available in the Nature Portfolio Reporting Summary linked to this article.

## Supplementary information

Supplementary Information

Transparent Peer Review file

Reporting Summary

## Source data

Source Data

## Data Availability

All data generated in this study has been deposited in the GEO: GSE288838. https://www.ncbi.nlm.nih.gov/geo/query/acc.cgi?acc=GSE288838. (Hi-C data for different stages). GEO: GSE288839. https://www.ncbi.nlm.nih.gov/geo/query/acc.cgi. (Micro-C data for different stages). GEO: GSE288837. https://www.ncbi.nlm.nih.gov/geo/query/acc.cgi. (CTCF Chip-seq). The published datasets used in this study are listed as follows: GEO: GSE121760. https://www.ncbi.nlm.nih.gov/geo/query/acc.cgi. (H3K4me3 and H3K27ac Chip-seq data). GEO: GSE107398. https://www.ncbi.nlm.nih.gov/geo/query/acc.cgi. (H3K27ac Chip-seq data). GEO: GSE75419. https://www.ncbi.nlm.nih.gov/geo/query/acc.cgi?acc=GSE75419. (Dataset for DSB hotspots used in B6XCAST mice). GEO: GSE74055. https://www.ncbi.nlm.nih.gov/geo/query/acc.cgi?acc=GSE74055. (Hi-C data for ES-E14). GEO: GSE98119. https://www.ncbi.nlm.nih.gov/geo/query/acc.cgi?acc=GSE98119. (Hi-C datasets for CH12_cells and activated_B_cells). GEO: GSE93431. https://www.ncbi.nlm.nih.gov/geo/query/acc.cgi. (Hi-C dataset for NIPBL KO cells). GEO: GSE122622. https://www.ncbi.nlm.nih.gov/geo/query/acc.cgi. (Hi-C dataset for mouse pachynema). GEO: GSE119805. https://www.ncbi.nlm.nih.gov/geo/query/acc.cgi?acc=GSE119805. (Hi-C dataset for mouse pachynema). GEO: GSE132054. https://www.ncbi.nlm.nih.gov/geo/query/acc.cgi?acc=GSE132054. (Hi-C dataset for mouse pachynema/diplonema). GEO: GSE147536. https://www.ncbi.nlm.nih.gov/geo/query/acc.cgi. (Hi-C dataset for mouse pachynema). Source data are provided with this paper.
