# Supplementary material for: High-resolution chromatin mapping reveals that CTCF anchors meiotic loops to the chromosome axis
Source: Nat Commun. 2026 Jul 28;17:7550. doi: 10.1038/s41467-026-73644-6 (PMC13416154; doi:10.1038/s41467-026-73644-6)
Supplement: Supplementary file 1 — Supplementary Information [file 41467_2026_73644_MOESM1_ESM.pdf]

Supplementary Figures

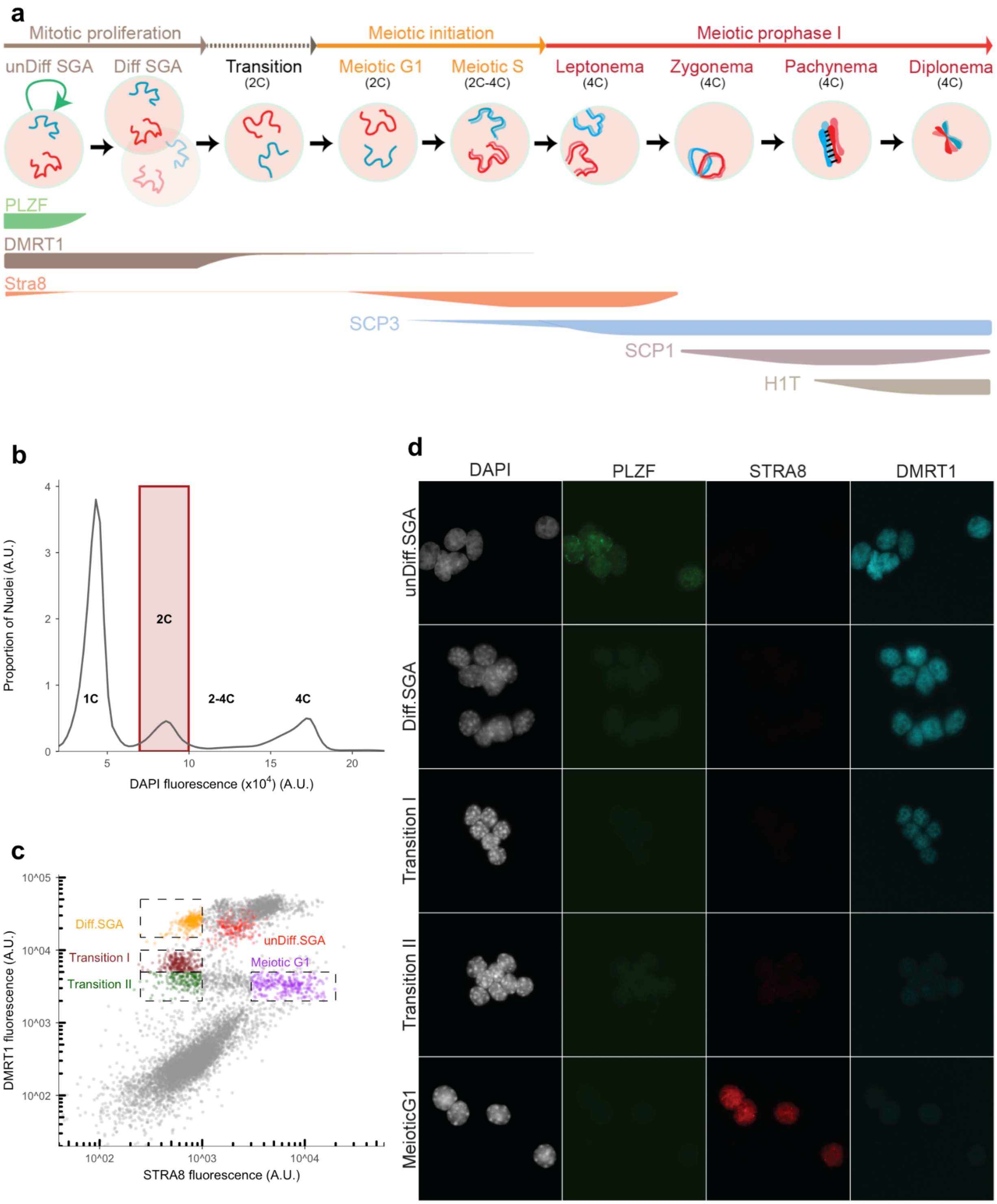

Supplementary Fig. 1: Isolation of stage specific nuclei through spermatogenesis.

(a) A schematic of the strategy for isolating nuclei from each stage. Intra-nuclear markers used for sorting were labeled with different colors. The relative expression level of each protein is represented as the thickness of their corresponding lines. Nuclei from unDiff.SGA to the Meiotic G1 stage were isolated with a combination of DAPI, PLZF, DMRT1 and STRA8. Nuclei from the meiotic S-phase stage to leptonema were isolated with a combination of DAPI, SCP3, DMRT1 and STRA8. Nuclei from the leptonema stage to diplonema were isolated with a combination of DAPI, SCP3, H1t and SCP1.

(b) Nuclei from unDiff.SGA to Meiotic G1 stage were isolated from the 2C population.

(c) A snapshot from flow cytometry illustrating the gating for each population. We identified three populations of nuclei along the continuum of expression of DMRT1 and STRA8 to represent a putative commitment trajectory of mitotic germ cells as they entered meiosis. The differentiating spermatogonia (Diff.SGA) were collected as nuclei that expressed high levels of DMRT1 and no STRA8. The mitotic-to-meiotic transition nuclei were identified as a population with reduced DMRT1 and slightly increased STRA8. To further examine the transition between mitotic and meiotic stages, the transition population of nuclei was separated into Transition I and Transition II stages based on the DMRT1 signal. Finally, the nuclei with increased STRA8 and reduced expression of DMRT1 were collected from the 2C population as Meiotic G1.

(d) Representative images of isolated nuclei from unDiff.SGA to the Meiotic G1 stage.

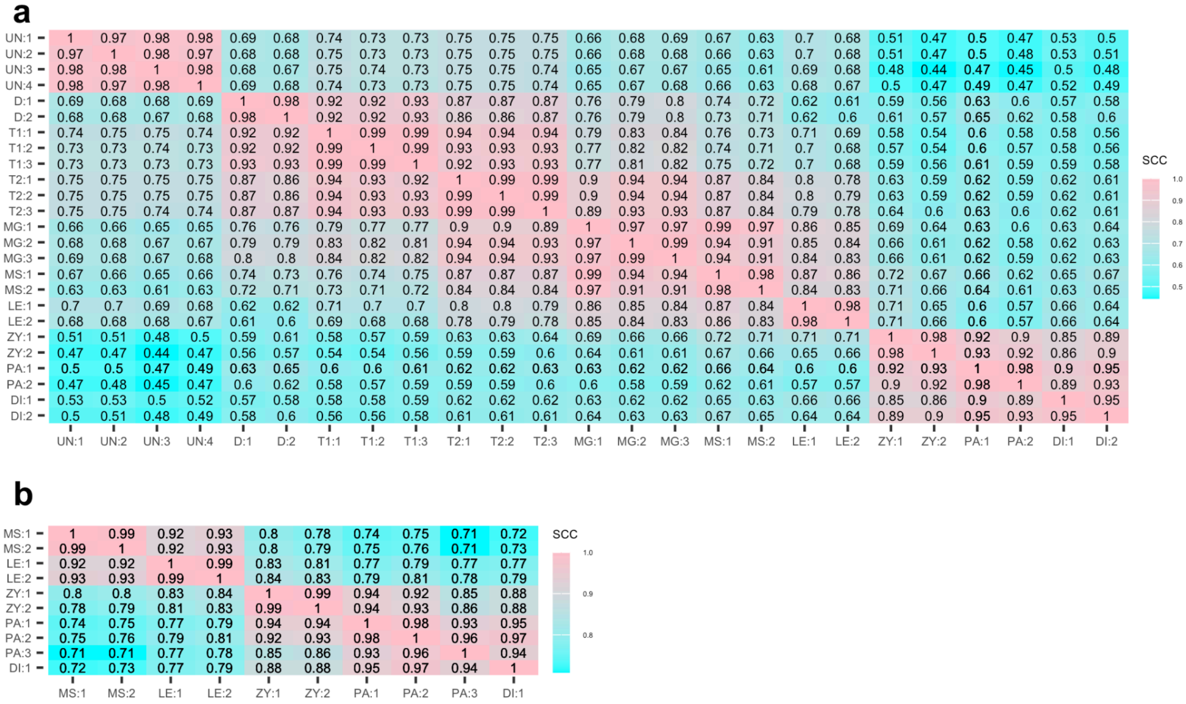

Supplementary Fig. 2: Reproducibility of the biological replicates.

(a) Reproducibility of Hi-C data.

(b) Reproducibility of Micro-C data.

The reproducibility was assessed by HiCRep[1,2]. The stratum-adjusted correlation coefficient (SCC) was calculated with a smooth parameter h=11, bin size=25,000 and maximal genomic distance to include in the calculation dBPMax = 500000.

UN=unDiff.SGA; D=Diff.SGA; T1=Transition I; T2=Transition II; MG=Meiotic G1; MS=Meiotic S; LE=Leptonema; ZY=Zygonema; PA=Pachynema; DI=Diplonema.

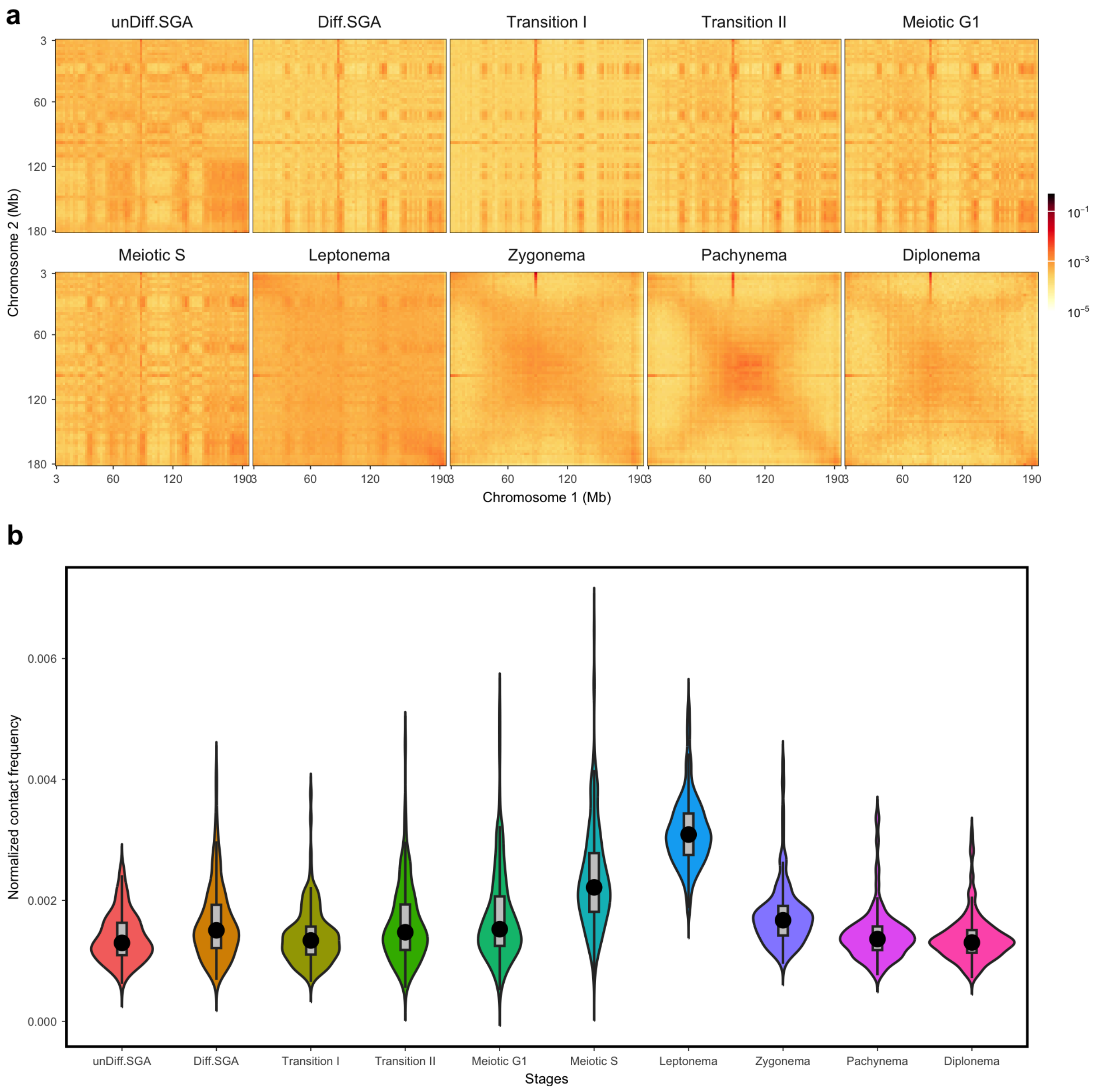

Supplementary Fig. 3: Inter-chromosomal interactions

(a) Matrices of contacts between chromosome 1 and chromosome 2 at a resolution of 2.5 Mb.

(b) Contact frequencies between chromosome ends were normalized as follows (n = 171 regions at 100 kb resolution for each stage). Because no detectable contacts were observed in the first 3 Mb of the p arm in the contact maps, this region was excluded from the analysis. Chromosome ends were defined as the first 5 Mb of the p arm and the last 5 Mb of the q arm. Mean interaction values between p-arm ends and between q-arm ends were calculated separately and then normalized by the mean cis-interaction frequencies within the

corresponding end regions. Finally, the normalized values for p- and q-arm ends were averaged and plotted as the normalized contact frequency. The inner box shows the interquartile range with the median indicated by the black dots. Boundaries of the embedded box: Represent the lower (25th) and upper (75th) quartiles. Black dot in the embedded box: Indicates the median value.

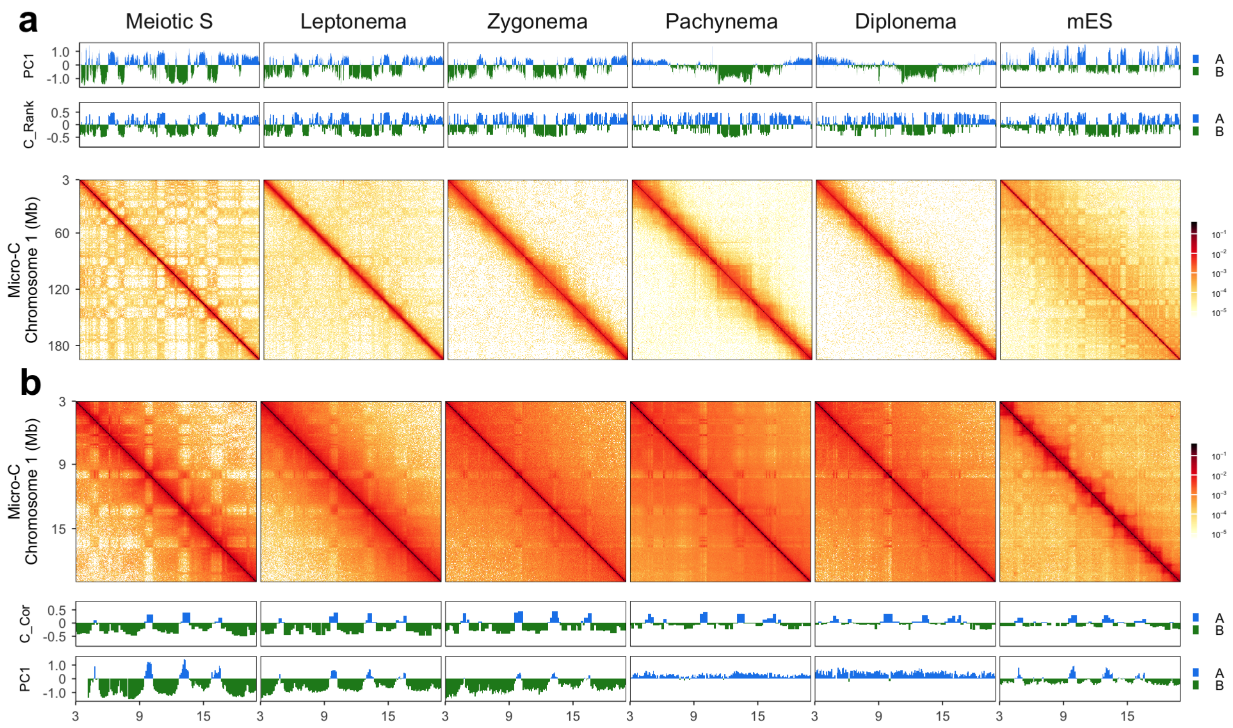

Supplementary Fig. 4: Genome-wide chromatin reorganization derived from Micro-C

(a) Top: The profiles of PC1 were calculated at a resolution of 100 Kb. Middle: The profiles of correlation scores calculated by Calder[3] at a resolution of 50 Kb. Bottom: Matrices of full chromosome 1 at a resolution of 100 Kb, plotted from Micro-C data.

(b) Zoom in of Micro-C matrices showing chromosome 1: 3-20 Mb. Top: Matrices were plotted at a resolution of 50 Kb from Micro-C data. Middle: The profiles of correlation scores were generated by Calder at a resolution of 50 Kb, aligned with the regions depicted in the Micro-C matrices. Bottom: The profiles of PC1 were calculated at a resolution of 100 Kb and aligned with the regions depicted in the Micro-C matrices.

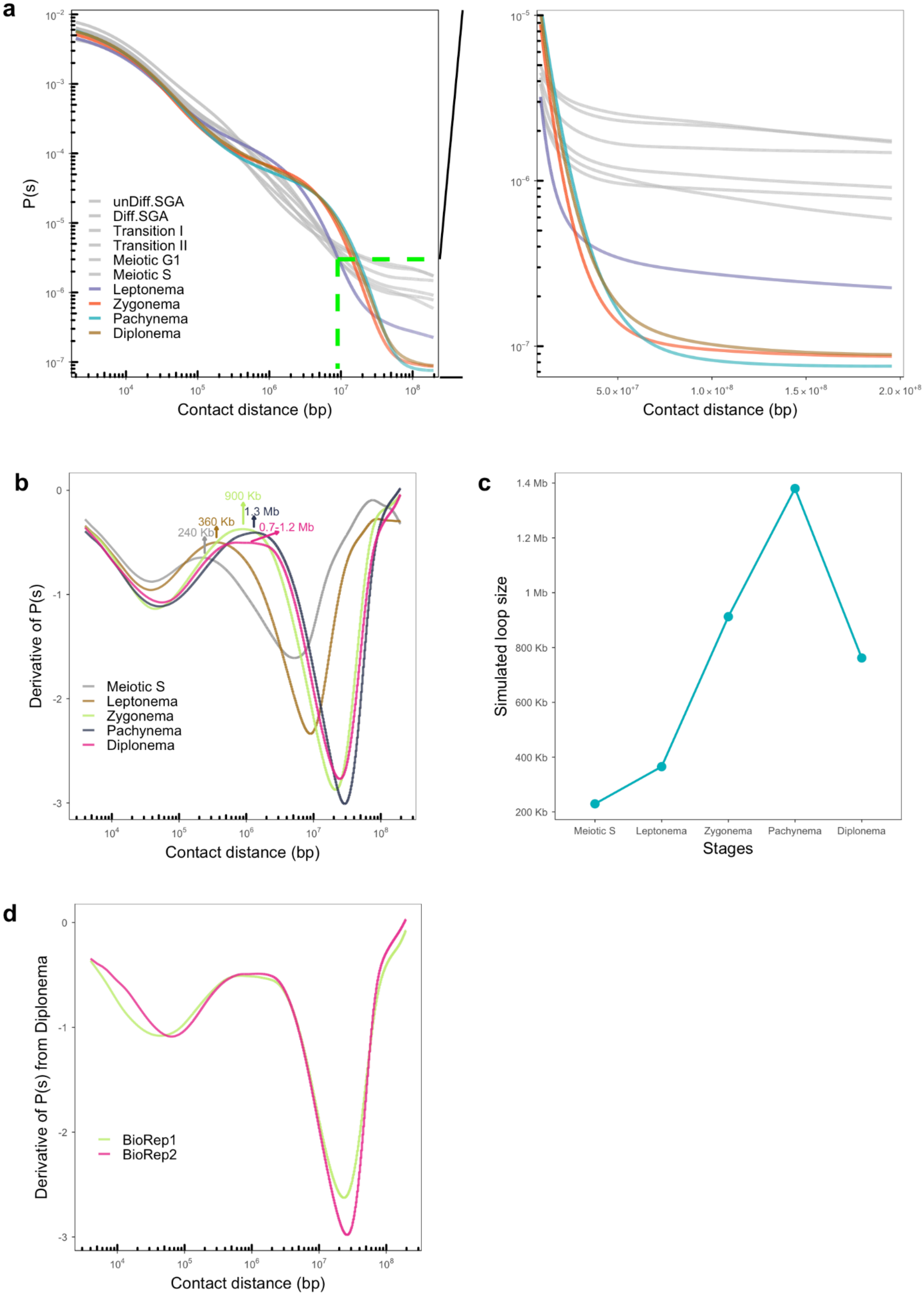
a
P(s)
unDiff.SGA
Diff.SGA
Transition I
Transition II
Meiotic G1
Meiotic S
Leptonema
Zygonema
Pachynema
Diplonema
Contact distance (bp)
Contact distance (bp)
b
Derivative of P(s)
900 Kb
1.3 Mb
360 Kb
0.7-1.2 Mb
240 Kb
Meiotic S
Leptonema
Zygonema
Pachynema
Diplonema
Contact distance (bp)
c
Simulated loop size
1.4 Mb
1.2 Mb
1 Mb
800 Kb
600 Kb
400 Kb
200 Kb
Meiotic S
Leptonema
Zygonema
Pachynema
Diplonema
Stages
d
Derivative of P(s) from Diplonema
BioRep1
BioRep2
Contact distance (bp)

Supplementary Fig. 5: Contact probability plotted as a function of contact distances and their corresponding derivatives

(a) Left: The contact probability was calculated at a resolution of 1 Kb. Stages from unDiff.SGA to Meiotic S are indicated by gray color. Stages during MPI are indicated by different colors. The x- and y-axis were plotted at log10 scale. Green dashed lines frame the region where far-cis contacts began to diminish. Right: The framed region was plotted separately. In this plot, the x-axis was plotted as a linear scale, the y-axis was plotted as a log10 scale.

(b) The derivatives of P(s) from meiotic S to diplotene stages. The local maxima are indicated as arrow bars, with the estimated loop sizes labeled above the arrows.

(c) Quantitative summary of Supplementary Fig. 5B. Simulated loop size is the contact distance of the first “bump”. The mean value of biological replicates was calculated.

(d) The derivatives of P(s) from two biological replicates of diplonema separately.

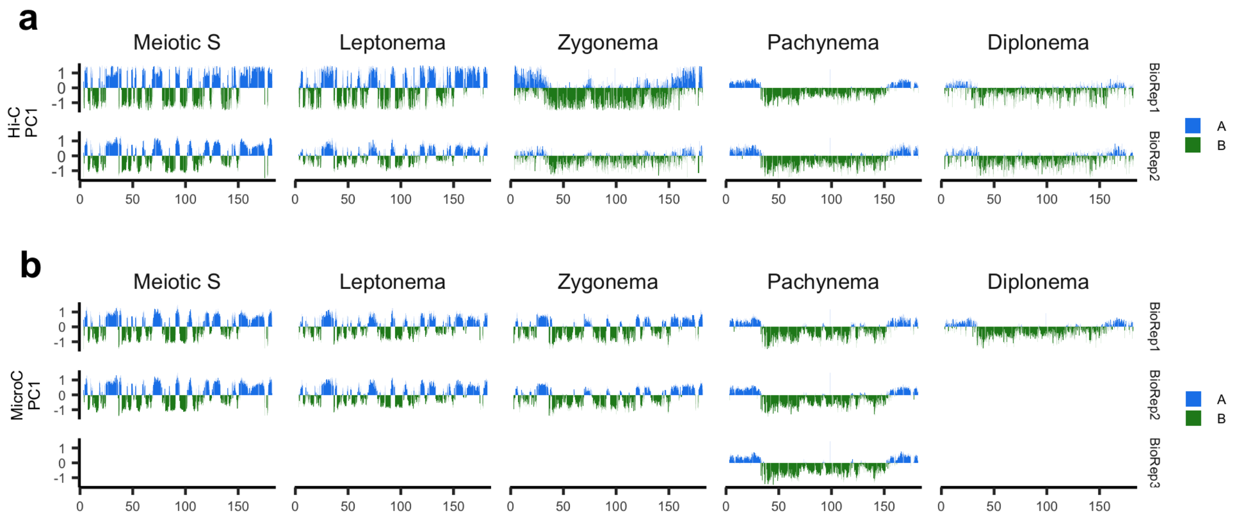

Supplementary Fig. 6: PC1 profiles derived from biological replicates of both Micro-C and Hi-C data

(a) PC1 along chromosome 2 for each replicate of Hi-C data. Principal component analysis was performed at a resolution of 100 Kb.

(b) PC1 along chromosome 2 for each replicate of Micro-C data. Principal component analysis was performed at a resolution of 100 Kb.

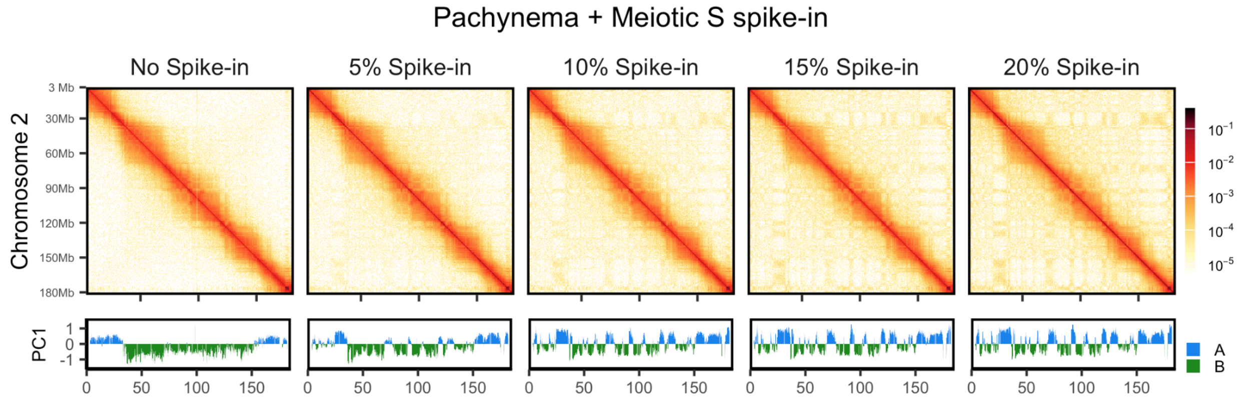

Supplementary Fig. 7. Identification of compartmentalization by Spike-in.

Compartmentalization was identified by spiking in interactions from the meiotic S stage. Various percentages of read pairs from the meiotic S stage were integrated with read pairs from the pachytene stage. Subsequently, matrices with a resolution of 100 Kb were constructed and balanced. The matrices for chromosome 2, which include different percentages of spike-in, were then plotted. Additionally, the first principal component (PC1) was calculated from the balanced matrices.

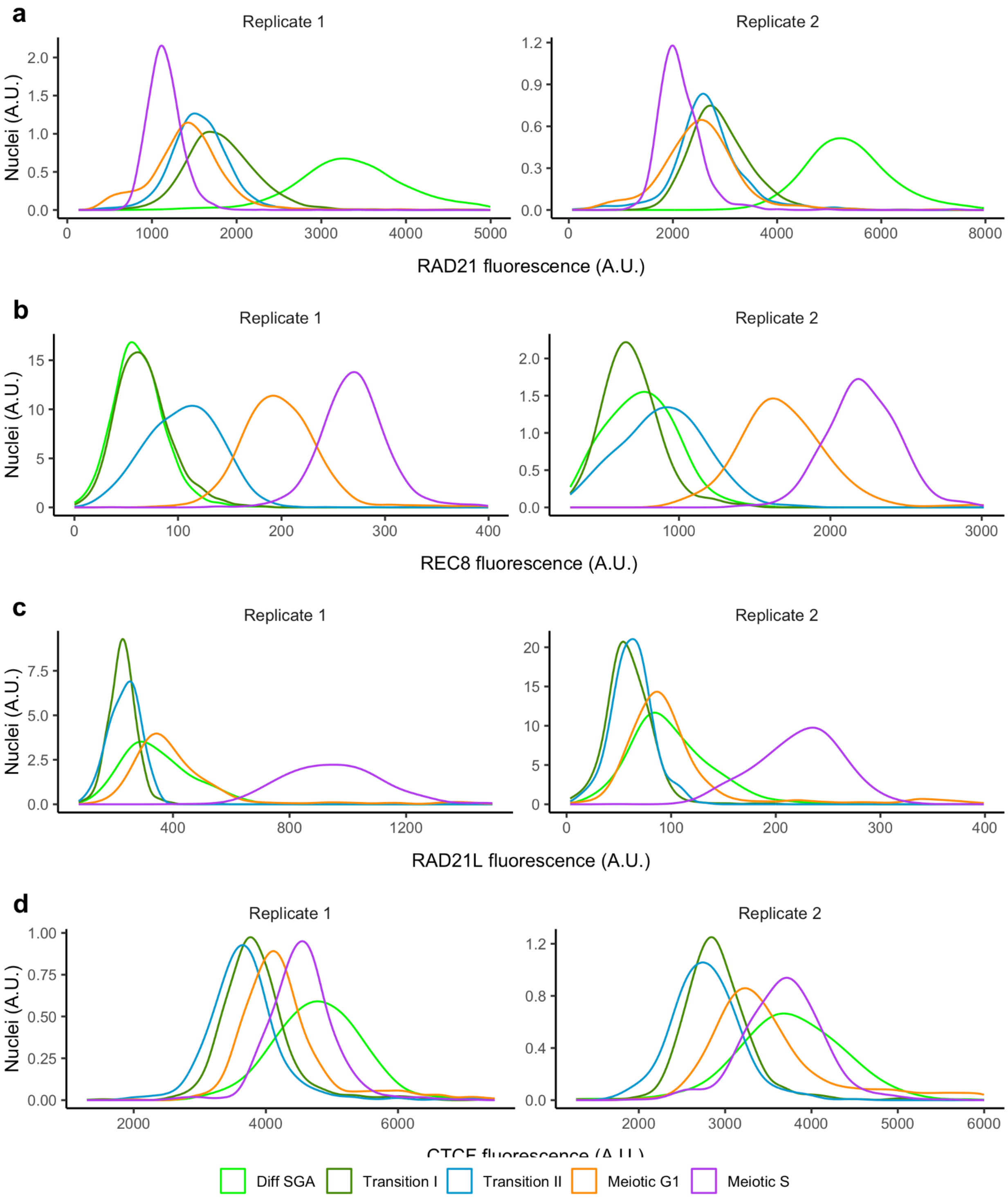

Supplementary Fig. 8: Changes in factors that regulate chromatin folding at the scale of TADs

(a) Changes in RAD21. The populations ranging from Diff.SGA to meiotic G1 were gated as shown in Supplementary Fig1. The meiotic S population was gated based on a 2-4C DNA content, very low DMRT1 expression, and very strong STRA8 expression. The profiles of RAD21 immunostaining signals from the different gated stages were plotted for two biological replicates.

(b) Changes in REC8. The populations were gated in the same manner as shown in (A). The profiles of REC8 immunostaining signals from the different gated stages were plotted for two biological replicates.

(c) Changes in RAD21L. The populations were gated in the same manner as shown in (A). The profiles of RAD21L immunostaining signals from the different gated stages were plotted for two biological replicates.

(d) Changes in CTCF. The populations were gated in the same manner as shown in (A). The profiles of CTCF immunostaining signals from the different gated stages were plotted for two biological replicates.

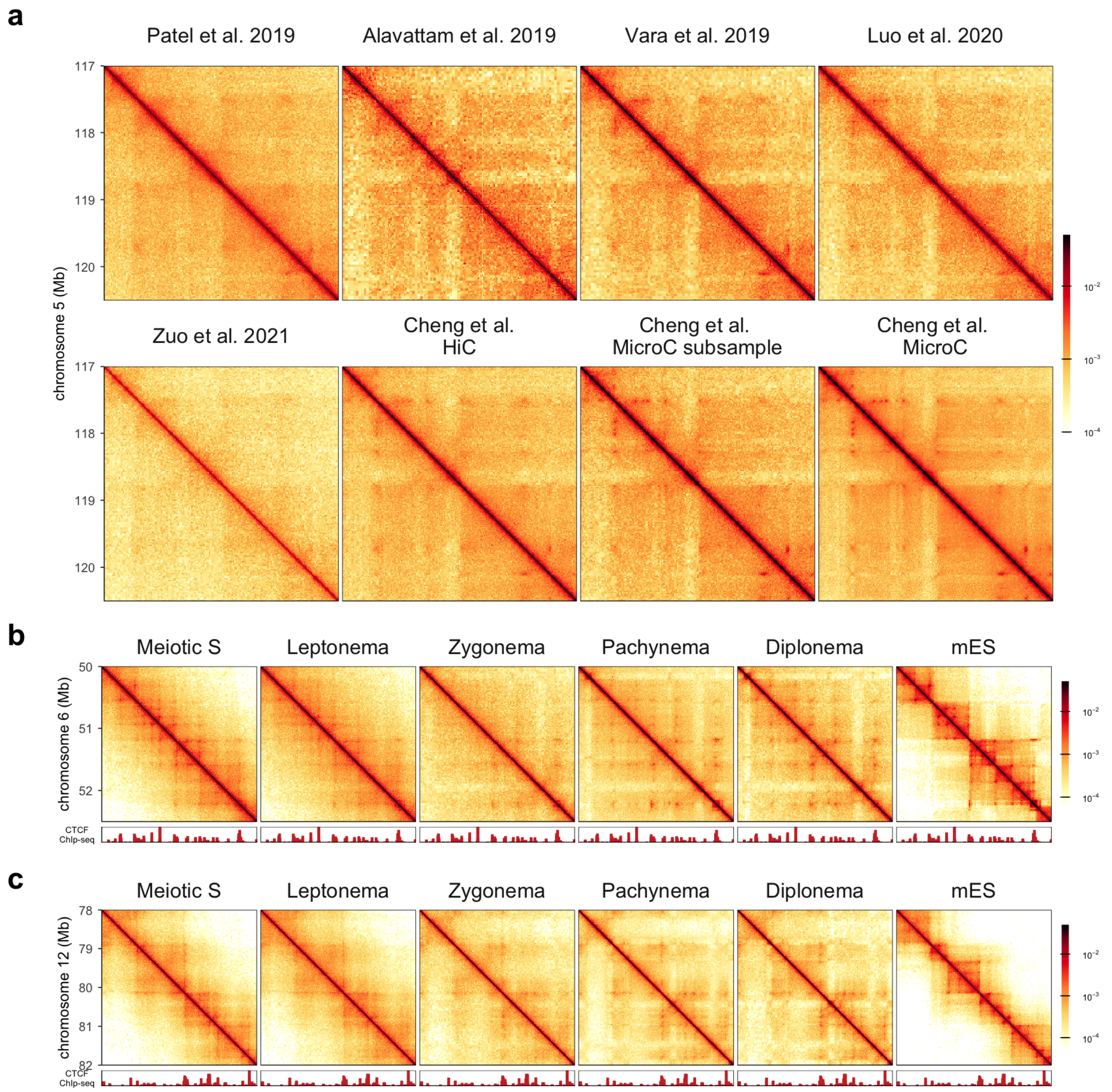

Supplementary Fig. 9: Dots visualization in Hi-C/Micro-C matrices generated from ours and published data.

(a) Pachytene matrices were plotted from previously published studies as well as our Hi-C/Micro-C data. The matrices show a region spanning from 117 Mb to 120 Mb on chromosome 5 at a resolution of 5 Kb. Patel et al. 2019: Data from Francesca Cole and Kevin D. Corbett's groups[4]. Total read pairs: 478 million; Alavattam et al. 2019: Data from Satoshi H Namekawa's group.[5] Total read pairs: 284 million; Vara et al. 2019: Data from Aurora Ruiz-Herrera's group[6]. Total read pairs: 411 million; Luo et al. 2020: Data from Xiaoyuan Song's group[7]. Total read pairs: 205 million; Zuo et al. 2021: Data from Ming Lei and Qian Bian's groups[8]. Total read pairs: 336 million; Cheng et al. HiC: Data from this study, R. Daniel Camerini-Otero group. Total read pairs: 634 million; Cheng et al. Micro-C subsample: Data from

this study, R. Daniel Camerini-Otero group. The total valid read pairs were subsampled to 690 million; Cheng et al. Micro-C: Data from this study, R. Daniel Camerini-Otero group. Total read pairs: 2.92 billions.

(b) A snapshot of the Micro-C matrix of chromosome 6 at a resolution of 5 Kb (region: 50 Mb to 52.5 Mb). CTCF ChIP coverage is plotted at the bottom.

(c) A snapshot of the Micro-C matrix of chromosome 12 at a resolution of 5 Kb (region: 78 Mb to 82 Mb). CTCF ChIP coverage is plotted at the bottom.

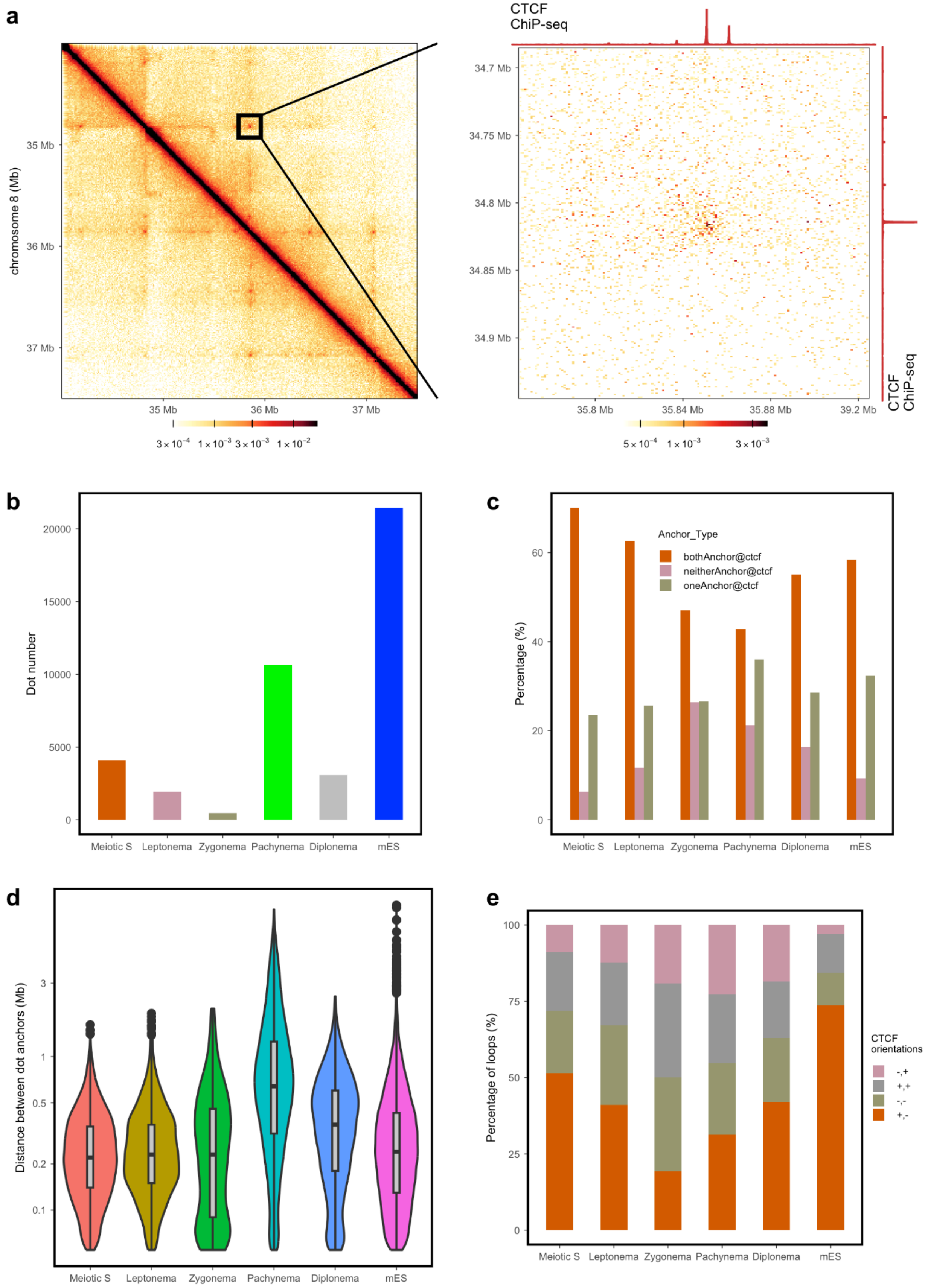
a
CTCF
ChiP-seq
chromosome 8 (Mb)
35 Mb
36 Mb
37 Mb
34.7 Mb
34.75 Mb
34.8 Mb
34.85 Mb
34.9 Mb
35.8 Mb
35.84 Mb
35.88 Mb
39.2 Mb
CTCF
ChiP-seq
b
Dot number
Meiotic S
Leptonema
Zygonema
Pachynema
Diplonema
mES
c
Percentage (%)
Anchor_Type
bothAnchor@ctcf
neitherAnchor@ctcf
oneAnchor@ctcf
d
Distance between dot anchors (Mb)
e
Percentage of loops (%)
CTCF
orientations

Supplementary Fig. 10: A summary of all dots identified from each stage

(a) Left: Micro-C matrix of pachynema on chromosome 8 at a resolution of 10 Kb (region: 34 Mb to 37.5 Mb). Right: the interaction matrix between region 35765000 to 35925000 and region 34945000 to 34685000 (indicated by black frame on the left). CTCF ChIP coverage is plotted at the top and right.

(b) The number of dots identified from each cell type. The anchors that are less than 50 Kb were removed because the calling at this range was not accurate enough.

(c) The percentage of dots with both anchors, one anchor, and no anchor overlapping with CTCF binding sites. CTCF motifs were identified from CTCF chip-seq peaks by FIMO. The motifs were extended +/- 5 kb when overlapping the anchors.

(d) Distances between anchors of identified dots (n = 3,809, 1,706, 330, 8,403, 2,569, and 19,460 for Meiotic S, leptonema, zygonema, pachynema, diplonema and mES respectively). Boundaries of the embedded box: Represent the lower (25th) and upper (75th) quartiles. Central horizontal line in the embedded box: Indicates the median value.

(e) Percentage of dots with anchors of different orientations. Anchors that overlap with a single CTCF motif were selected for this analysis.

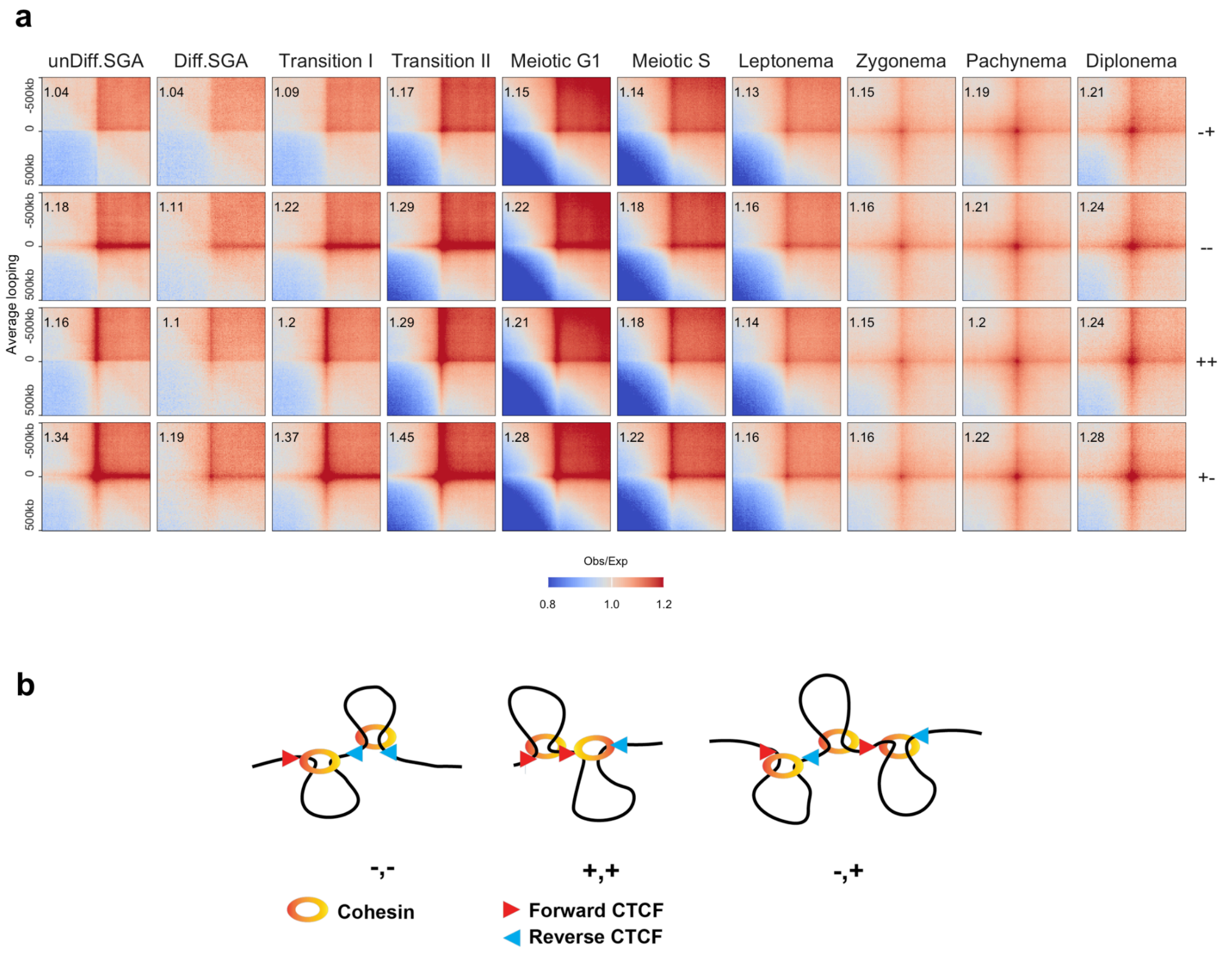

Supplementary Fig. 11: Aggregated interactions of pairwise CTCF from different orientations.

(a) Aggregated interactions between CTCF of different orientations. Each map is plotted at a resolution of 10 Kb, with a flanking region of 500 Kb. Enrichment was calculated as the mean of the central 3×3 pixels and shown in the top-left corner.

(b) A schematic to show how the Hi-C loops are formed between non-convergent CTCFs. This schematic is adapted from[9]. Left: A cohesin-mediated loop anchored by reverse-facing CTCF may encounter a loop anchored by convergent CTCFs, potentially resulting in the detection of loops defined by two reverse-facing CTCFs. Middle: A cohesin-mediated loop anchored by forward-facing CTCF may encounter a loop anchored by convergent CTCFs, potentially resulting in the detection of loops defined by two forward-facing CTCFs. Right: A cohesin-mediated loop may encounter two loops anchored by convergent CTCFs, potentially resulting in the detection of loops defined by reverse-facing CTCFs and forward-facing CTCFs.

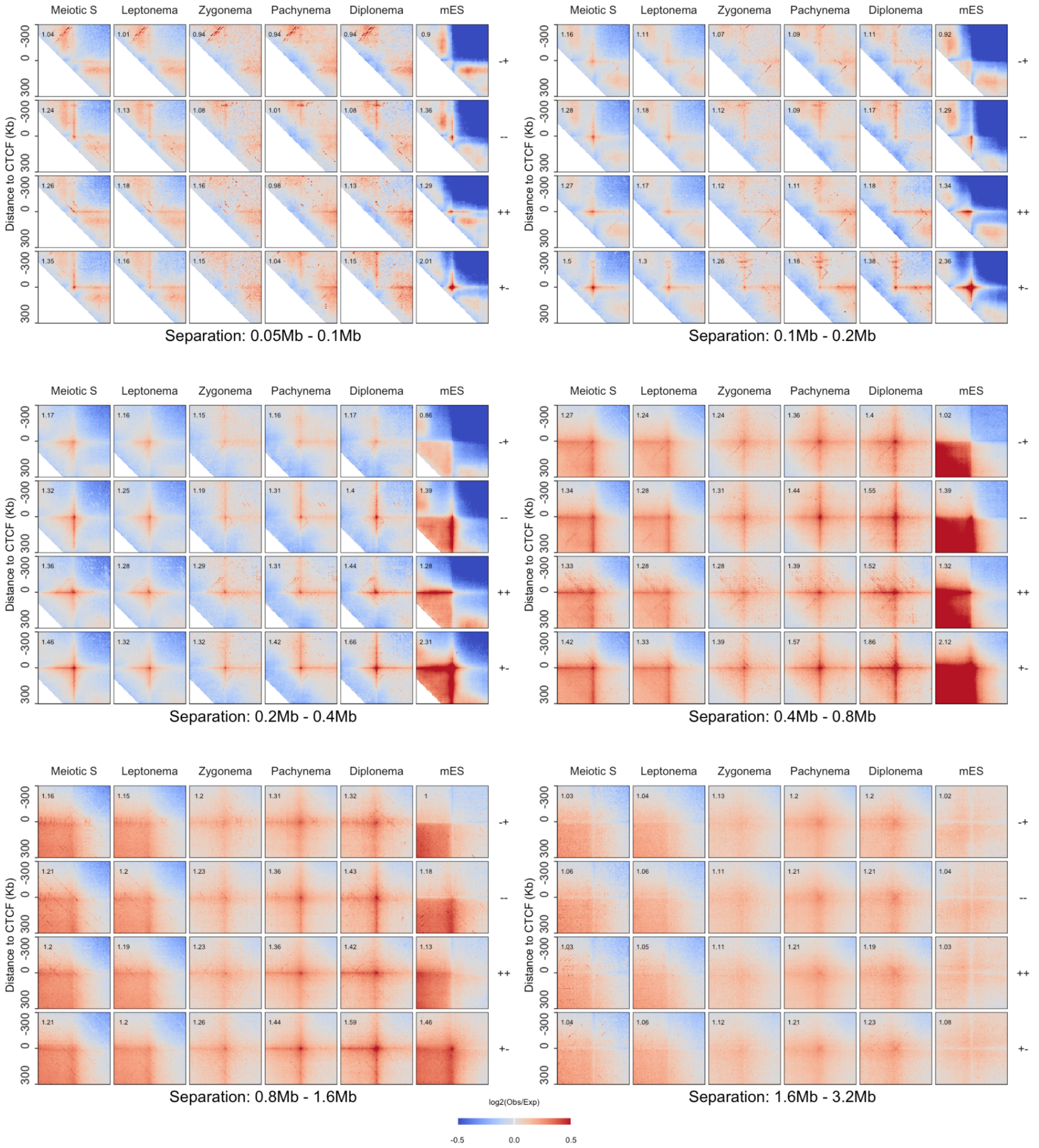

Supplementary Fig. 12: Aggregation of pairwise CTCF binding sites categorized by orientation and distance.

Aggregated interactions between CTCF binding sites were plotted at a resolution of 10 Kb, with a flanking region of 300 Kb. The orientation was labeled at the right of each panel. +: CTCF

binding motifs with forward orientation; -: CTCF binding motifs with reverse orientation. The separation between two CTCF binding motifs was labeled at the bottom of each panel. Enrichment was calculated as the mean of the central 3×3 pixels and shown in the top-left corner.

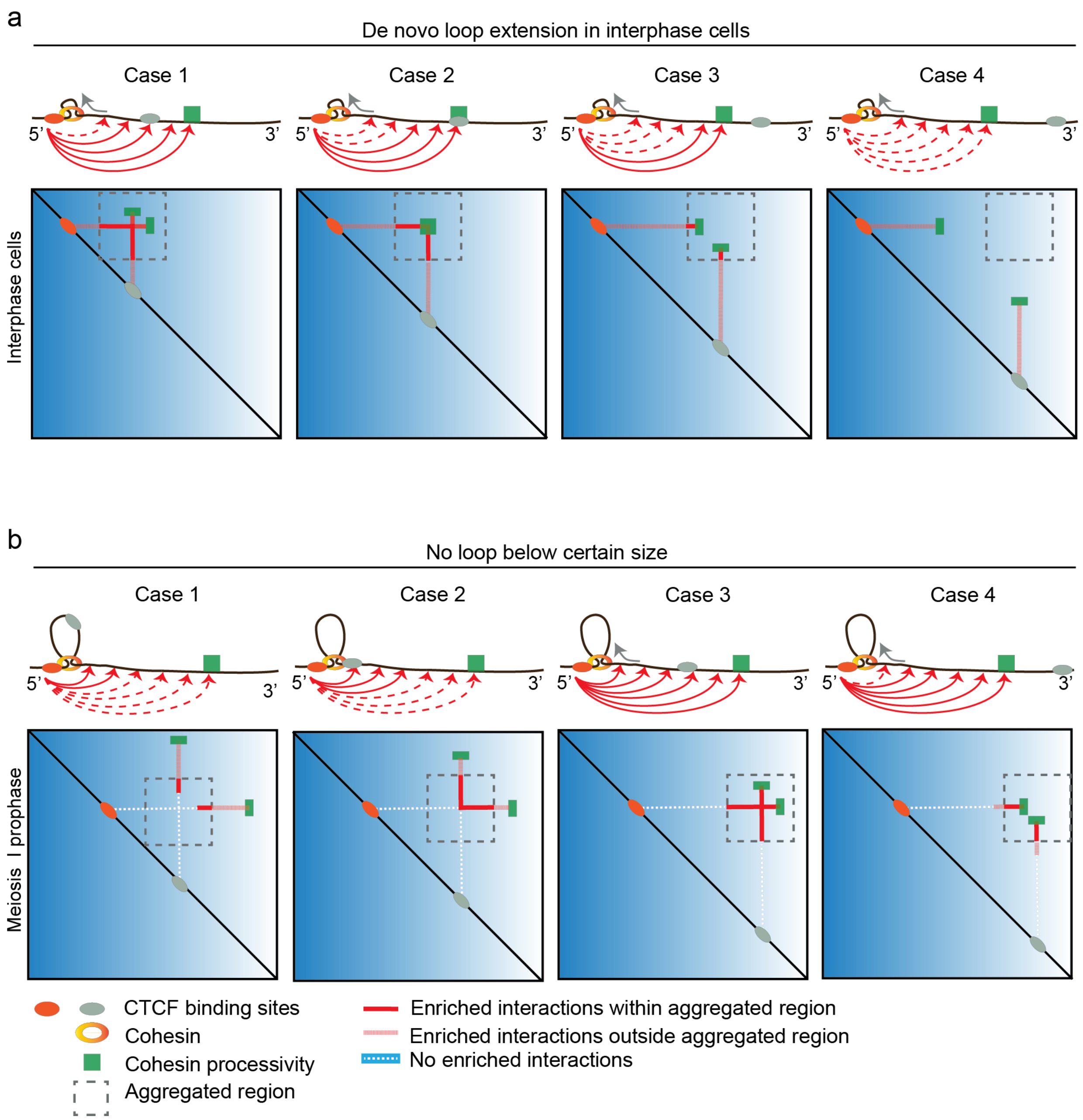

Supplementary Fig. 13: The schematic illustrates the enriched interaction pattern at various distances between CTCF binding sites and cohesin processivity.

(A) In somatic interphase cells, cohesin complexes can translocate along chromatin and extrude loops. The N-terminal of CTCF interacts with cohesin, stabilizing its binding to DNA. High interaction frequencies are observed between forward-facing CTCF-bound loci (indicated by red ellipse) and their downstream sequences (indicated by multiple red curves with an arrow), up to the maximum processivity of cohesin (indicated by green bar). The backward-facing CTCF bound loci (indicated by grey ellipse) interact with their upstream sequence at high frequency (not indicated in the schematic). Aggregation analysis of pairwise interactions defines a flanking

region centered on the joint point to examine average interaction density (indicated by grey dashed-line box). The interactions captured within the defined region vary depending on the distance between CTCF-bound loci, and are described as follows: In Case I, the distance between CTCF-bound loci is smaller than the maximum processivity of cohesin. CTCF-bound loci (indicated by red/grey ellipses) interact with downstream/upstream sequences at high frequency, up to the maximum processivity of cohesin (indicated by the green bar). The enriched interaction will pass the CTCF joined sites (indicated by red line). The interactions captured by a defined grey dashed-line box is indicated as a solid red line. The interactions that are not captured by dash lines. In Case 2, the distance between CTCF-bound loci is the same as the maximum processivity of cohesin. CTCF-bound loci (indicated by red/grey ellipses) interact with downstream/upstream sequences at high frequency, up to the maximum processivity of cohesin (indicated by the green bar). The enriched interactions converge at the CTCF-joined sites (indicated by the red line). Interactions captured within the defined grey dashed-line box are represented by a solid red line, while interactions outside the dashed region are indicated as a dash red line. In Case 3, the distance between CTCF-bound loci is bigger than the maximum processivity of cohesin. Despite this, enriched interactions can still be captured within the defined region. CTCF-bound loci (indicated by red/grey ellipses) interact with downstream/upstream sequences at high frequency, up to the maximum processivity of cohesin (indicated by the green bar). However, the enriched interactions cannot converge at the CTCF-joined sites (indicated by the red line). Interactions captured within the defined grey dashed-line box are represented by a solid red line, while interactions outside the dashed region are indicated as a dash red line. In Case 4, the distance between CTCF-bound loci is bigger than the maximum processivity of cohesin. Meanwhile, enriched interactions cannot be captured within the defined region. CTCF-bound loci (indicated by red/grey ellipses) interact with downstream/upstream sequences at high frequency, up to the maximum processivity of cohesin (indicated by the green bar). No enriched interactions are captured within the defined grey dashed-line box.

(B) During meiosis I prophase, interactions between CTCF-bound loci and regions at short distances are disrupted. The interaction patterns at varying distances between CTCF sites can be described as follows: In Case I, the distance between CTCF-bound loci is smaller than the size of the preformed loop. No enriched interactions are detected within the range of performed loop size (indicated by white dash line). CTCF-bound loci can contact regions beyond the range, up to the maximum contact distance (indicated by green bar). Interactions captured within the defined grey dashed-line box are represented by a solid red line, while interactions outside the dashed region are indicated as a dash red line. The enriched interactions cannot converge at the CTCF-joined sites (indicated by the red line). In Case 2, the distance between CTCF-bound loci is equal to the size of the preformed loop. No enriched interactions are detected within the range of performed loo size (distance between CTCF size) (indicated by white dash line).

CTCF-bound loci can contact regions beyond the range, up to the maximum contact distance (indicated by green bar). Interactions captured within the defined grey dashed-line box are represented by a solid red line, while interactions outside the dashed region are indicated as a dash red line. The enriched interactions converge at the CTCF-joined sites (indicated by the red line). In Case 3, the distance between CTCF-bound loci is bigger than the size of the preformed loop, and smaller than the maximum contact distance that cohesin can reach. No enriched interactions are detected within this range (indicated by white dash line). CTCF-bound loci can contact regions beyond the range, up to the maximum contact distance (indicated by green bar). Interactions captured within the defined grey dashed-line box are represented by a solid red line, while interactions outside the dashed region are indicated as a dash red line. The enriched interactions can be detected within two CTCF sites. The interactions converge and pass the CTCF-joined sites (indicated by the red line). In Case 4, the distance between CTCF-bound loci is bigger than the size of the preformed loop, and bigger than the maximum contact distance that cohesin can reach. No enriched interactions are detected within this range (indicated by white dash line). CTCF-bound sizes can contact regions beyond the range, up to the maximum contact distance (indicated by green bar). Interactions captured within the defined grey dashed-line box are represented by a solid red line, while interactions outside the dashed region are indicated as a dash red line. The enriched interactions can be detected within two CTCF sites. However, the interactions cannot converge at the CTCF-joined sites (indicated by the red line).

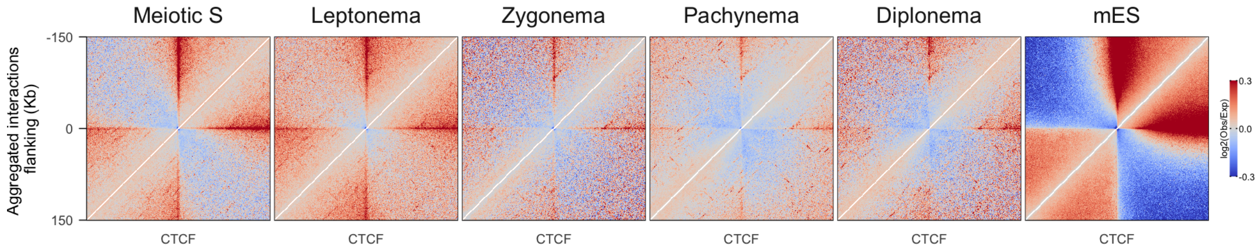

Supplementary Fig. 14: A zoomed-in view of Fig. 4e illustrating the depletion of short-range interaction enrichment.

Aggregation of interactions surrounding CTCF binding sites with unified motif orientation. The analysis was performed at a resolution of 1 Kb, with a 150 Kb flanking region. Only the interactions at loci with forward-oriented CTCF was shown.

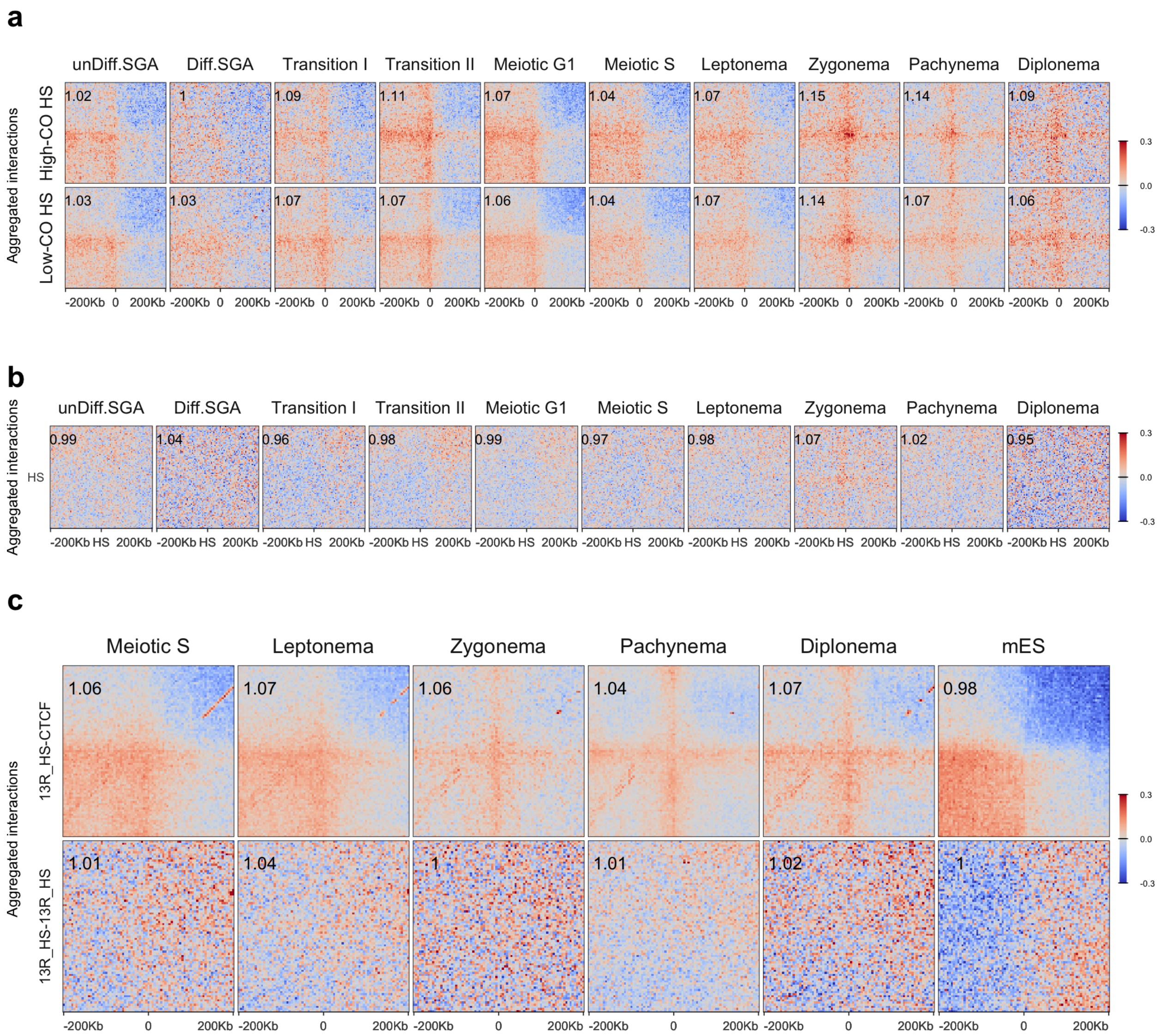

Supplementary Fig. 15: Aggregated hotspot HS-CTCF and HS-HS interactions.

(a) Aggregated interactions between DSB hotspots and CTCF bound loci derived from Hi-C contact maps. The same dataset from Fig. 5c was used in this plot. Enrichment was calculated as the mean of the central 3×3 pixels and shown in the top-left corner.

(b) Aggregated interactions between hotspots derived from Hi-C contact maps. Hotspots were derived from85. The same dataset from Fig. 5d was used in this plot. Enrichment was calculated as the mean of the central 3×3 pixels and shown in the top-left corner.

(c) Aggregated interactions between 13R HS-CTCF and13R HS- 13RHS derived from Micro-C contact maps. Enrichment was calculated as the mean of the central 3×3 pixels and shown in the top-left corner.

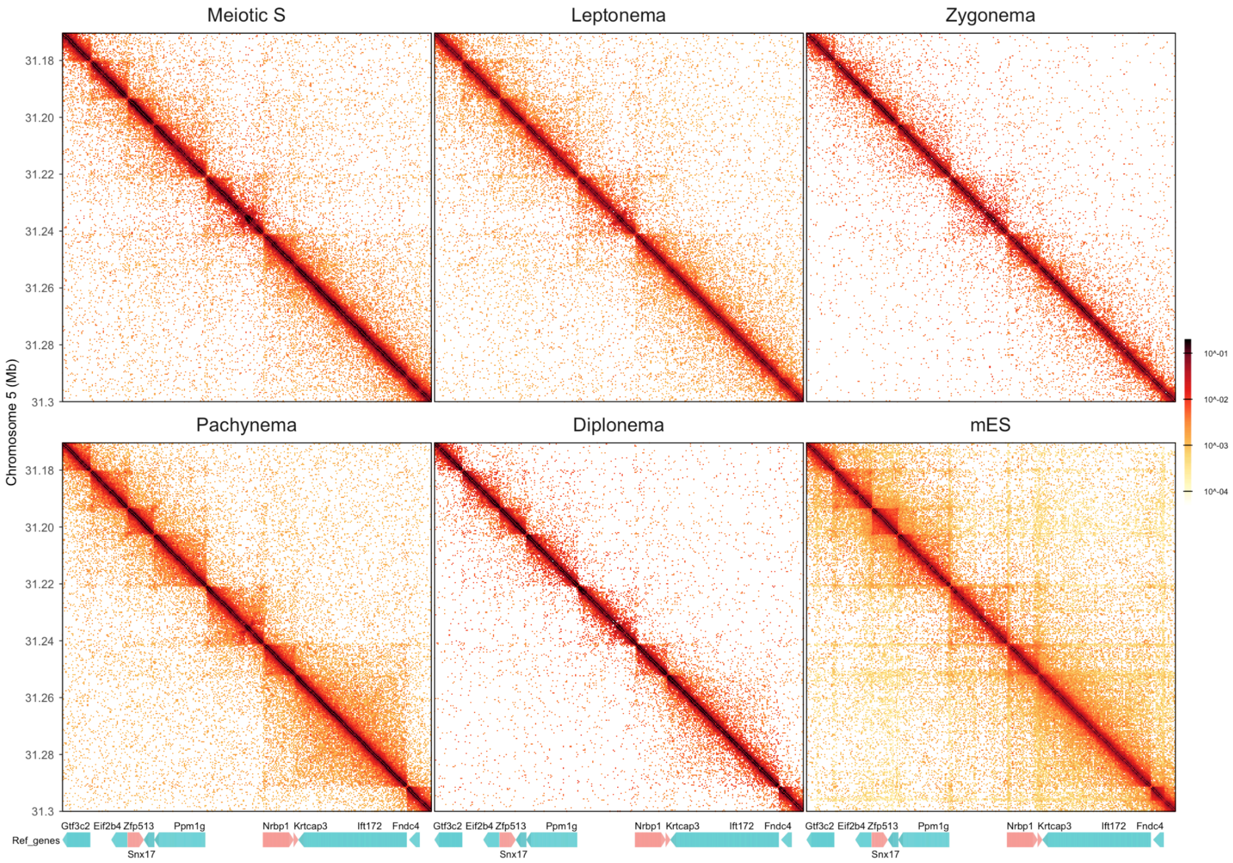

Supplementary Fig. 16: Snapshots of Micro-C matrices at a resolution of 400 bp

Micro-C matrices from meiotic S-phase through diplonema were plotted for the region spanning 31 Mb to 31.4 Mb on chromosome 5 at a resolution of 400 bp. The Micro-C matrix of the same region from mES[10] was plotted for comparison. Genes at this locus were annotated at the bottom. Bars with arrows indicate the orientation of the genes. The difference of visibility mainly results from sequencing depth.

Meiotic S-phase: 1371 million read pairs

Leptonema: 1348 million read pairs

Zygonema: 752 million read pairs

Pachynema: 3263 million read pairs

Diplonema: 996 million read pairs

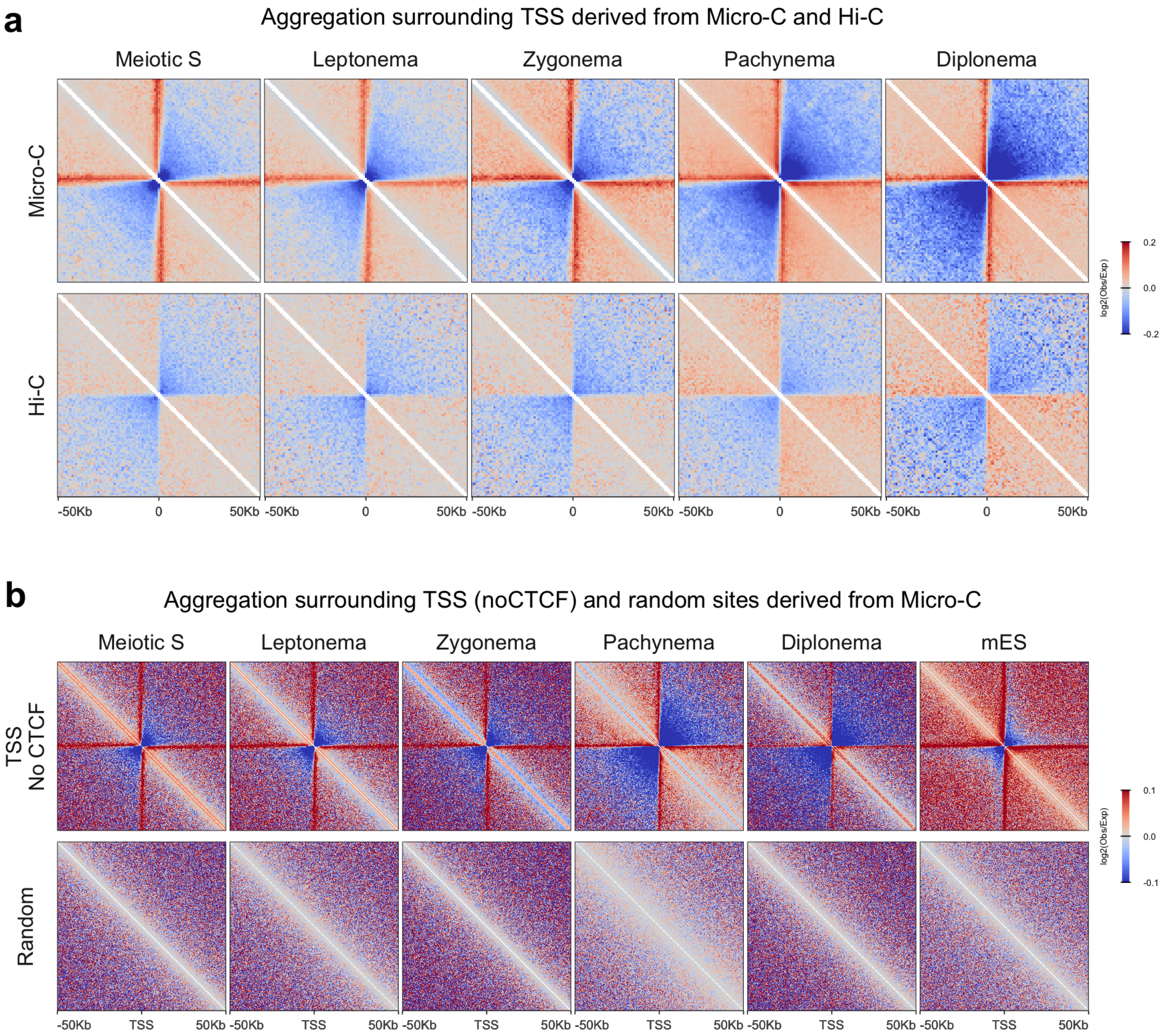

Supplementary Fig. 17: Aggregated interactions surrounding TSSs.

(A) Aggregated interactions centered around TSSs were calculated for both Micro-C and Hi-C data. TSSs were derived from USCS annotated genes. The aggregated interactions were calculated at a resolution of 200 bp with a flanking region of 50 Kb.

(B) Aggregated interactions centered around TSSs and random sites were calculated for Micro-C data. TSSs were derived from USCS annotated genes. Any TSS with CTCF binding sites within ± 5 Kb were and avoided in this analysis. The aggregated interactions were calculated at a resolution of 200 bp with a flanking region of 50 Kb.

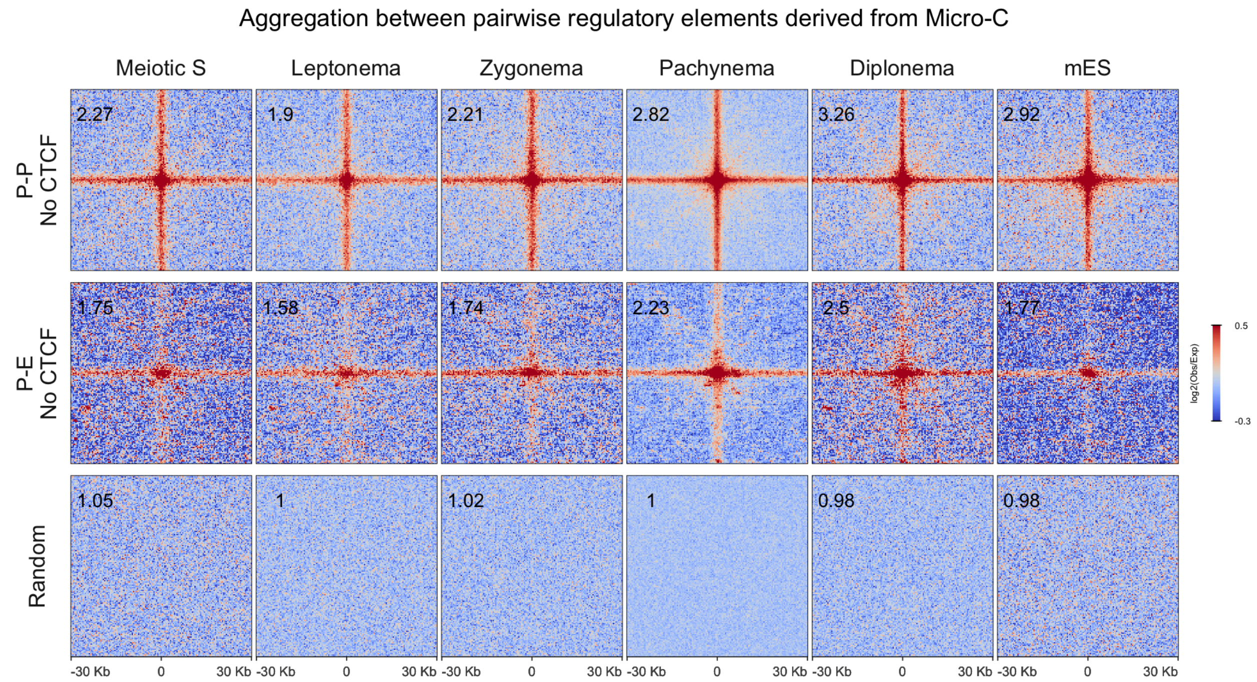

Supplementary Fig. 18: Aggregation of P-P/P-E interactions that do not overlap with CTCF.

Similar to Fig. 6, promoters were identified based on H3K4me3 ChIP-seq data[11]. Enhancers were defined as the common peaks found in two separate H3K27ac ChIP-seq datasets[11,12]. Peaks from these datasets that did not overlap with H3K4me3 peaks were considered enhancers. Furthermore, both promoters and enhancers that coincided with CTCF binding sites within a range of ± 5 Kb were excluded from the calculations. Two promoters, or promoters and enhancers, at distances ranging from 5 Kb to 5 Mb were paired and aggregated at a resolution of 1000 bp with a flanking region of 50 Kb. Enrichment was calculated as the mean of the central 3×3 pixels and shown in the top-left corner.

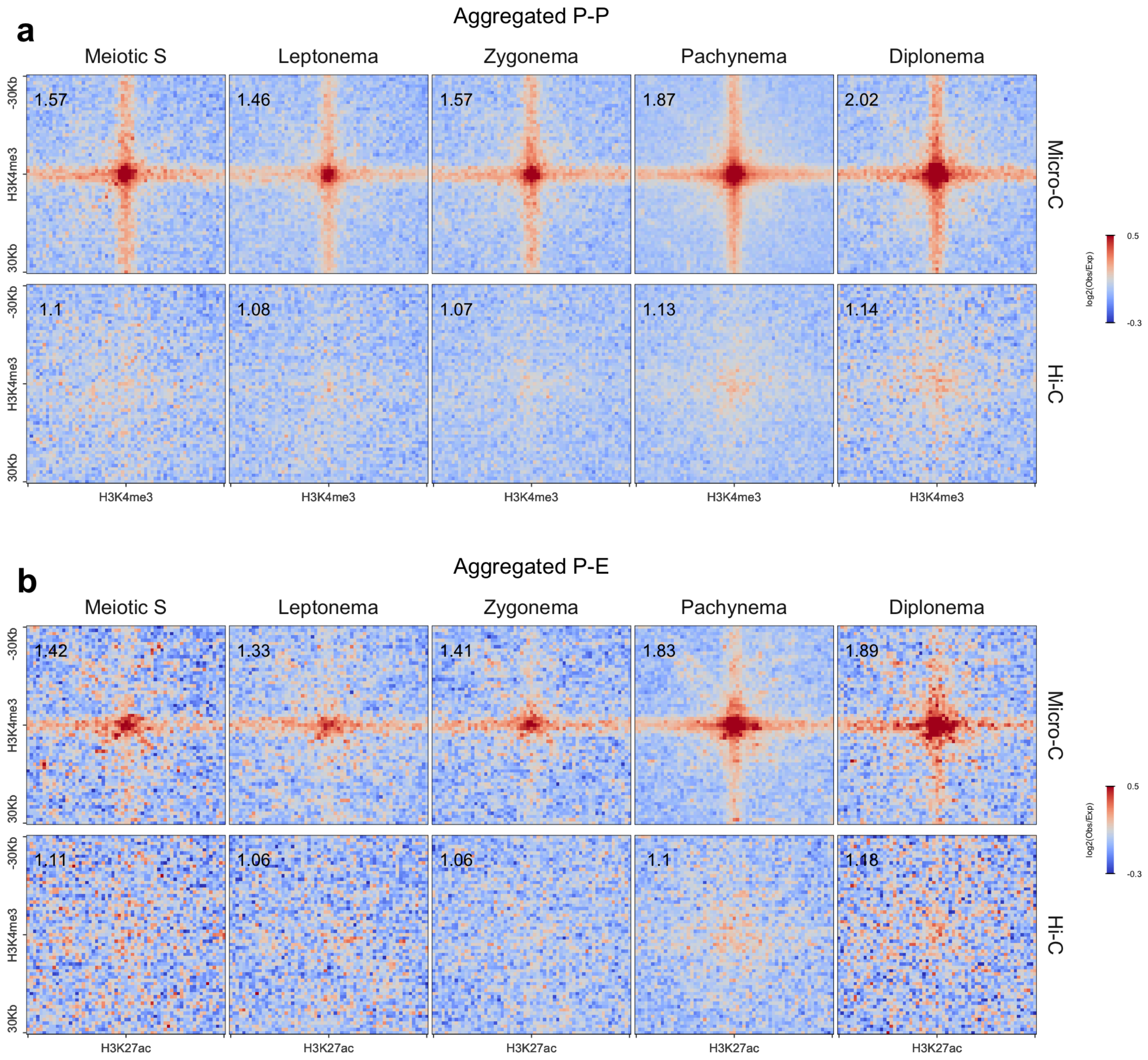

Supplementary Fig. 19: Aggregated interactions between regulatory elements.

(A) Aggregated promoter-promoter interactions were plotted for both Micro-C and Hi-C data. The promoters were derived from H3K4me3 Chip-seq data[11]. The H3K4me3 modification sites that overlapped with hotspots were excluded from calculations. Two promoters at distances ranging from 5 Kb to 5 Mb were paired and aggregated at a resolution of 1000 bp with a flanking region of 50 Kb. Enrichment was calculated as the mean of the central 3×3 pixels and shown in the top-left corner.

(B) Aggregated promoter-enhancer interactions were plotted for both Micro-C and Hi-C data. The enhancers were derived from H3K27ac Chip-seq data[11,12]. Common peaks from both studies were selected and only the sites that did not overlap with H3K4me3 peaks and hotspots

were used for the calculation. Promoters and enhancers at distances ranging from 5 Kb to 5 Mb were paired and aggregated at a resolution of 1000 bp with a flanking region of 50 Kb. Enrichment was calculated as the mean of the central 3×3 pixels and shown in the top-left corner.

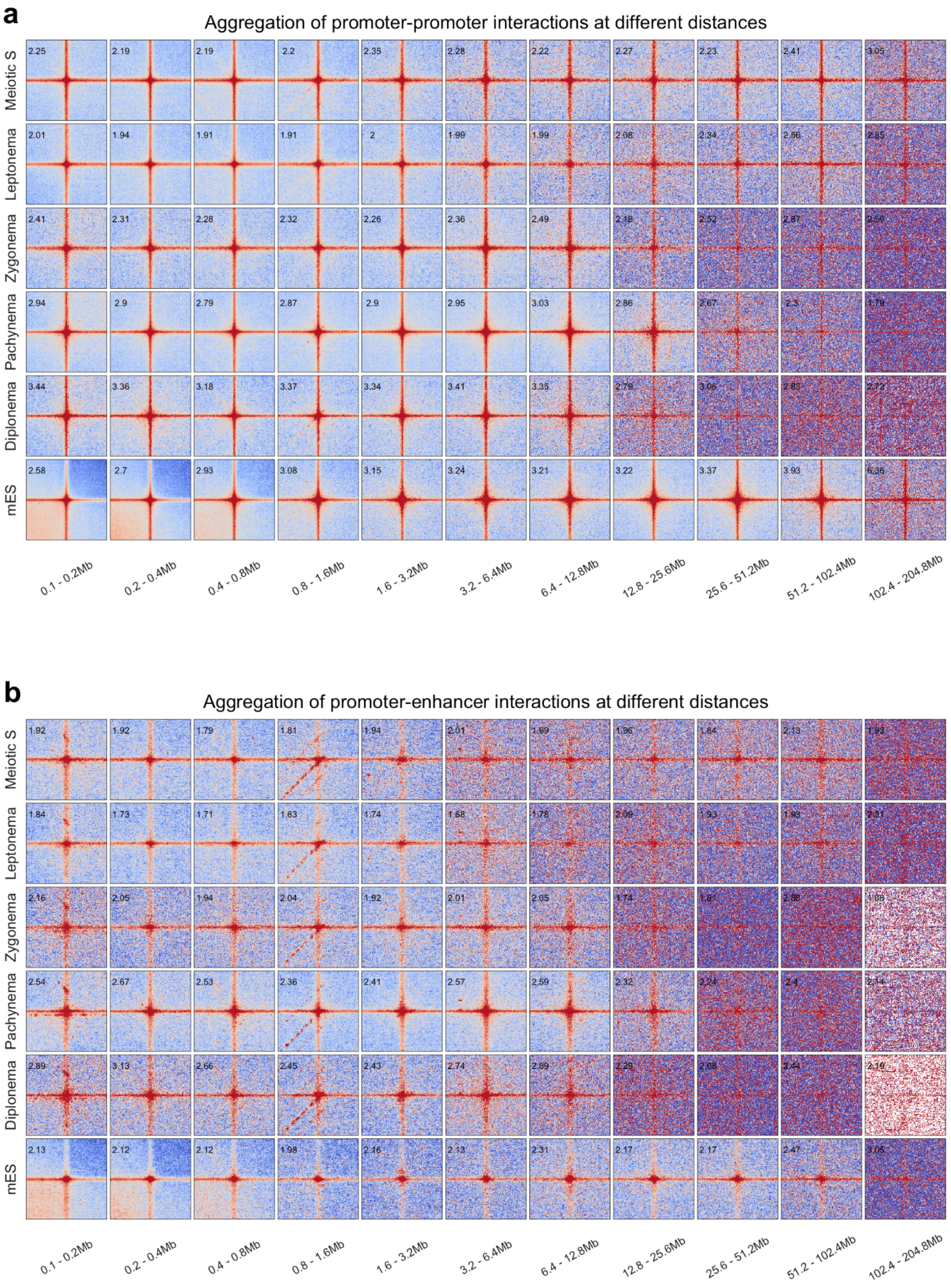
a
Aggregation of promoter-promoter interactions at different distances
Meiotic S
Leptonema
Zygonema
Pachynema
Diplonema
mES
0.1 - 0.2Mb
0.2 - 0.4Mb
0.4 - 0.8Mb
0.8 - 1.6Mb
1.6 - 3.2Mb
3.2 - 6.4Mb
6.4 - 12.8Mb
12.8 - 25.6Mb
25.6 - 51.2Mb
51.2 - 102.4Mb
102.4 - 204.8Mb
b
Aggregation of promoter-enhancer interactions at different distances
Meiotic S
Leptonema
Zygonema
Pachynema
Diplonema
mES
0.1 - 0.2Mb
0.2 - 0.4Mb
0.4 - 0.8Mb
0.8 - 1.6Mb
1.6 - 3.2Mb
3.2 - 6.4Mb
6.4 - 12.8Mb
12.8 - 25.6Mb
25.6 - 51.2Mb
51.2 - 102.4Mb
102.4 - 204.8Mb

Supplementary Fig. 20: Aggregation of P-P/P-E interactions at different distances.

(A) Aggregated promoter-promoter interactions at different distances. The same promoter dataset in Fig. 6 was used here. Paired promoters were categorized based on distances and aggregated at a resolution of 400 bp with a flanking region of 30 Kb. Enrichment was calculated as the mean of the central 3×3 pixels and shown in the top-left corner.

(B) Aggregated promoter-enhancer interactions at different distances. The same promoters and enhancer dataset in Fig. 6 were used here. Paired promoter-enhancers were categorized based on distances and aggregated at a resolution of 400 bp with a flanking region of 30 Kb. Enrichment was calculated as the mean of the central 3×3 pixels and shown in the top-left corner.

Aggregation of interactions between promoters on different chromosomes

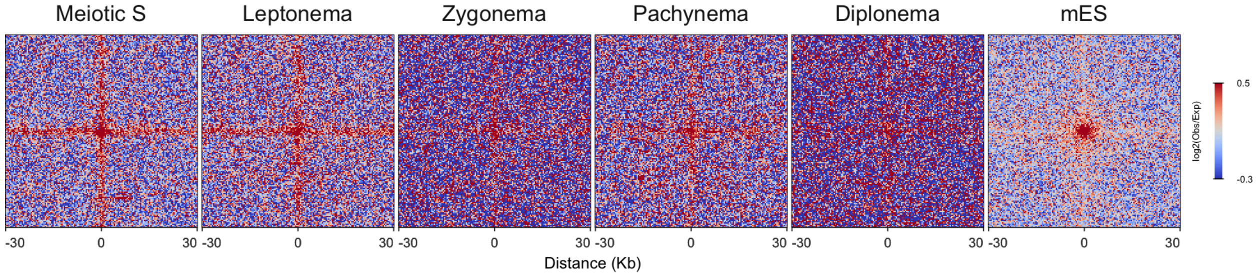

Supplementary Fig. 21. Aggregation of interactions at promoters from different chromosomes.

The same promoter dataset in Fig. 6 was used here. All promoters from different chromosomes were paired and aggregated at a resolution of 400 bp with a flanking region of 30 Kb.

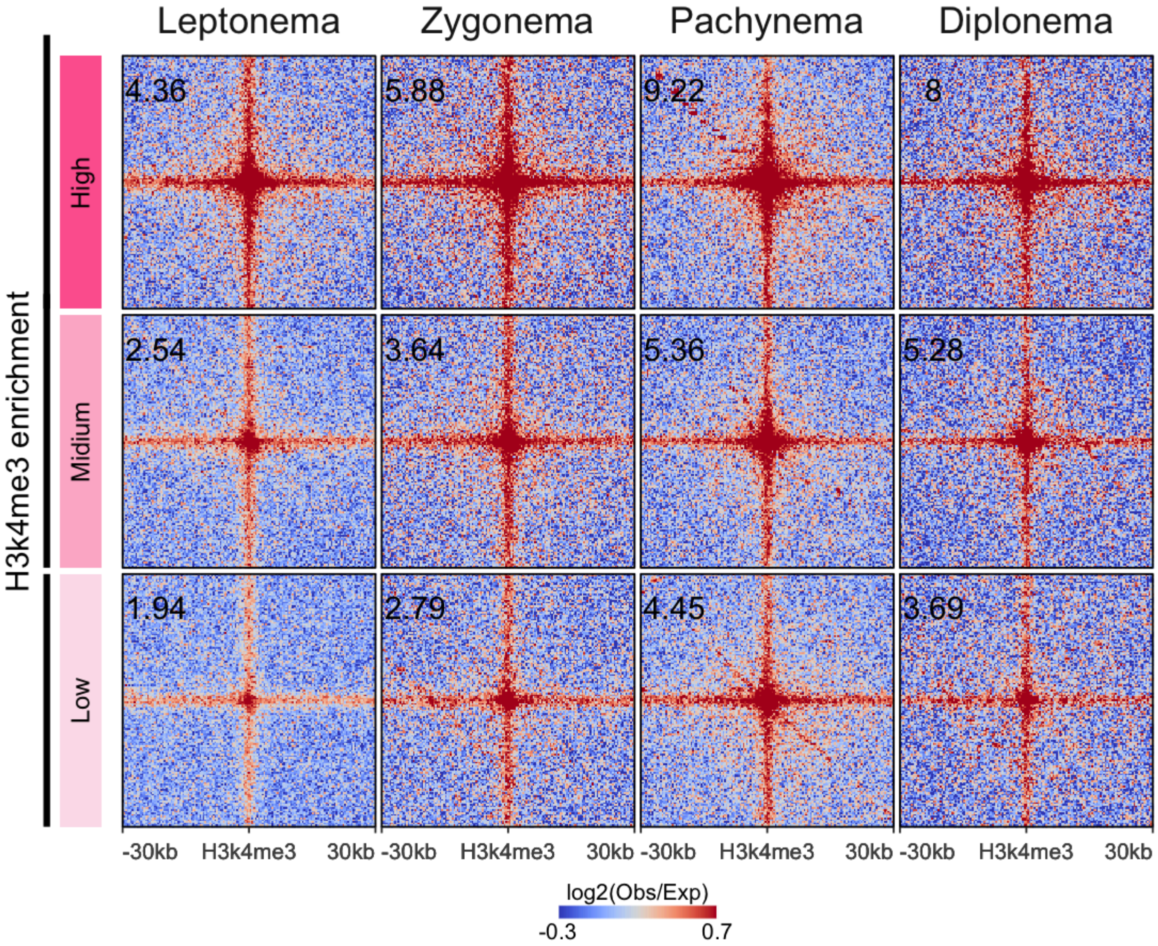

Supplementary Fig. 22. The correlation between H3k4me3 enrichment and P-P interactions.

The same promoter dataset in Fig. 6 was used here. Promoters from each stage were divided into three groups based on their enrichment derived from Chip-seq[11] different chromosomes were paired and aggregated at a resolution of 400 bp with a flanking region of 30 Kb. The aggregated interactions were calculated at a resolution of 400 bp with a flanking region of 30 Kb. Enrichment was calculated as the mean of the central 3×3 pixels and shown in the top-left corner.

Supplementary References

1. Yang, T. *et al.* HiCRep: assessing the reproducibility of Hi-C data using a stratum-adjusted correlation coefficient. *Genome Res.* **27**, 1939–1949 (2017).
2. Lin, D., Sanders, J. & Noble, W. S. HiCRep.py : Fast comparison of Hi-C contact matrices in Python. *Bioinformatics* (2021) doi:10.1093/bioinformatics/btab097.
3. Liu, Y. *et al.* Systematic inference and comparison of multi-scale chromatin sub-compartments connects spatial organization to cell phenotypes. *Nat. Commun.* **12**, 2439 (2021).
4. Patel, L. *et al.* Dynamic reorganization of the genome shapes the recombination landscape in meiotic prophase. *Nat. Struct. Mol. Biol.* **26**, 164–174 (2019).
5. Alavattam, K. G. *et al.* Attenuated chromatin compartmentalization in meiosis and its maturation in sperm development. *Nat. Struct. Mol. Biol.* **26**, 175–184 (2019).
6. Vara, C. *et al.* Three-Dimensional Genomic Structure and Cohesin Occupancy Correlate with Transcriptional Activity during Spermatogenesis. *Cell Rep.* **28**, 352–367.e9 (2019).
7. Luo, Z. *et al.* Reorganized 3D Genome Structures Support Transcriptional Regulation in Mouse Spermatogenesis. *iScience* **23**, 101034 (2020).
8. Zuo, W. *et al.* Stage-resolved Hi-C analyses reveal meiotic chromosome organizational features influencing homolog alignment. *Nat. Commun.* **12**, 5827 (2021).
9. Liu, N. Q. *et al.* Rapid depletion of CTCF and cohesin proteins reveals dynamic features of chromosome architecture. *bioRxiv* 2021.08.27.457977 (2021) doi:10.1101/2021.08.27.457977.
10. Hsieh, T.-H. S. *et al.* Resolving the 3D Landscape of Transcription-Linked Mammalian Chromatin Folding. *Mol. Cell* **78**, 539–553.e8 (2020).
11. Lam, K.-W. G., Brick, K., Cheng, G., Pratto, F. & Camerini-Otero, R. D. Cell-type-specific genomics reveals histone modification dynamics in mammalian meiosis. *Nat. Commun.* **10**,

3821 (2019).

12. Maezawa, S. *et al.* Super-enhancer switching drives a burst in gene expression at the mitosis-to-meiosis transition. *Nat. Struct. Mol. Biol.* **27**, 978–988 (2020).

Table 1: Hi-C & Micro-C library summary					
Assay	Stage	BioRep	Cis	Trans	Total_nodups
Hi-C	unDiff.SGA	BioRep1	110,247,554	22,706,971	132,954,525
Hi-C	unDiff.SGA	BioRep2	77,625,696	15,112,498	92,738,194
Hi-C	unDiff.SGA	BioRep3	389,171,364	73,435,628	462,606,992
Hi-C	unDiff.SGA	BioRep4	205,241,520	45,898,910	251,140,430
Hi-C	unDiff.SGA	Merged	782,286,134	157,154,007	939,440,141
Hi-C	Diff.SGA	BioRep1	166,079,137	37,399,412	203,478,549
Hi-C	Diff.SGA	BioRep2	202,120,898	53,089,426	255,210,324
Hi-C	Diff.SGA	Merged	368,200,035	90,488,838	458,688,873
Hi-C	Tansition I	BioRep1	246,104,913	54,455,858	300,560,771
Hi-C	Tansition I	BioRep2	350,652,421	70,213,954	420,866,375
Hi-C	Tansition I	BioRep3	209,984,270	46,378,987	256,363,257
Hi-C	Tansition I	Merged	806,741,604	171,048,799	977,790,403
Hi-C	Tansition II	BioRep1	159,296,580	37,070,176	196,366,756
Hi-C	Tansition II	BioRep2	404,672,332	91,948,883	496,621,215
Hi-C	Tansition II	BioRep3	195,996,013	51,859,652	247,855,665
Hi-C	Tansition II	Merged	759,964,925	180,878,711	940,843,636
Hi-C	Meiotic G1	BioRep1	306,382,803	64,004,583	370,387,386
Hi-C	Meiotic G1	BioRep2	323,142,633	64,646,951	387,789,584
Hi-C	Meiotic G1	BioRep3	205,397,034	49,339,705	254,736,739
Hi-C	Meiotic G1	Merged	834,922,470	177,991,239	1,012,913,709
Hi-C	Meiotic S	BioRep1	296,907,167	90,770,711	387,677,878
Hi-C	Meiotic S	BioRep2	86,113,377	18,385,498	104,498,875
Hi-C	Meiotic S	Merged	383,020,544	109,156,209	492,176,753
Hi-C	Leptonema	BioRep1	275,499,642	91,331,945	366,831,587
Hi-C	Leptonema	BioRep2	72,199,098	16,210,845	88,409,943
Hi-C	Leptonema	Merged	347,698,740	107,542,790	455,241,530
Hi-C	Zygonema	BioRep1	244,058,507	48,013,940	292,072,447
Hi-C	Zygonema	BioRep2	87,732,970	13,550,966	101,283,936
Hi-C	Zygonema	Merged	331,791,477	61,564,906	393,356,383
Hi-C	Pachynema	BioRep1	465,105,229	73,480,330	538,585,559
Hi-C	Pachynema	BioRep2	84,087,976	11,522,156	95,610,132
Hi-C	Pachynema	Merged	549,193,205	85,002,486	634,195,691
Hi-C	Diplonema	BioRep1	96,323,836	13,093,145	109,416,981
Hi-C	Diplonema	BioRep2	81,003,256	9,645,138	90,648,394
Hi-C	Diplonema	Merged	177,327,092	22,738,283	200,065,375
Micro-C	Meiotic S	BioRep1	281,193,788	20,166,313	301,360,101
Micro-C	Meiotic S	BioRep2	1,003,055,250	67,080,846	1,070,136,096
Micro-C	Meiotic S	Merged	1,284,249,038	87,247,159	1,371,496,197
Micro-C	Leptonema	BioRep1	264,407,682	20,126,367	284,534,049
Micro-C	Leptonema	BioRep2	990,771,688	73,548,031	1,064,319,719
Micro-C	Leptonema	Merged	1,255,179,370	93,674,398	1,348,853,768
Micro-C	Zygonema	BioRep1	348,489,867	18,122,953	366,612,820
Micro-C	Zygonema	BioRep2	365,753,549	19,908,737	385,662,286
Micro-C	Zygonema	Merged	714,243,416	38,031,690	752,275,106
Micro-C	Pachynema	BioRep1	268,445,009	13,889,201	282,334,210
Micro-C	Pachynema	BioRep2	1,348,441,974	71,829,628	1,420,271,602
Micro-C	Pachynema	BioRep3	1,321,184,572	48,894,231	1,370,078,803
Micro-C	Pachynema	Merged	2,938,071,555	134,613,060	3,072,684,615
Micro-C	Diplonema	BioRep1	867,414,667	32,469,802	899,884,469
Micro-C	Diplonema	Merged	867,414,667	32,469,802	899,884,469

Supplementary Table 1. Hi-C and Micro-C library summary.

Table 2: Summary of chromatin conformation studied by Hi-C during mammalian spermatogenesis							
	Patel.et.al..2019	Wang.et.al..2019	Alavattam.et.al..2019	Vara.et.al..2019	Luo.et.al..2020	Zuo.et.al..2021	He.et.al..2023
Species	Mouse (C57BL/6J X CAST/EiJ)	Rhesus monkey	Mouse (C57BL/6J)	Mouse (C57BL/6J)	Mouse (C57BL/6J)	Mouse (C57BL/6J)	Mouse (C57BL/6J X PWK/PhJ)
Stages studied in MPI	Zygonema, Pachynema	Pachynema	Pachynema	Leptonema/Zygonema Pachynema/Diplonema	Pachynema	Leptonema, Zygonema, Pachynema, Diplonema	Leptonema, Zygonema, Pachynema, Diplonema
Interphase-like Compartmentalization	Yes, attenuated	Yes, attenuated	Yes, attenuated	lost during Pachynema/Diplonema	Yes, attenuated	Yes, attenuated	lost during Pachynema reapper in Diplonema
TADs	No	No	Yes, attenuated, large	Yes, attenuated, large	No	No	Mostly lost during zygonema remain attenuated large TADs
Hi-C loops	A few loops detected	Not detected	Not detected	Not detected	Not detected	Not detected	Not detected

Supplementary Table 2. Summary of chromatin conformation studied by Hi-C during mammalian spermatogenesis.
