# Supplementary material for: High-resolution chromatin mapping reveals that CTCF anchors meiotic loops to the chromosome axis
Source: Nat Commun. 2026 Jul 28;17:7550. doi: 10.1038/s41467-026-73644-6 (PMC13416154; doi:10.1038/s41467-026-73644-6)
Supplement: Supplementary file 3 — Reporting Summary [file 41467_2026_73644_MOESM3_ESM.pdf]

nature portfolio

Corresponding author(s): *Double-anonymous peer review submissions: write DAPR and your manuscript number here instead of author names.*

Last updated by author(s): *YYYY-MM-DD*

Reporting Summary

Nature Portfolio wishes to improve the reproducibility of the work that we publish. This form provides structure for consistency and transparency in reporting. For further information on Nature Portfolio policies, see our Editorial Policies and the Editorial Policy Checklist.

Statistics

For all statistical analyses, confirm that the following items are present in the figure legend, table legend, main text, or Methods section.

n/a	Confirmed	
☐	☒	The exact sample size (*n*) for each experimental group/condition, given as a discrete number and unit of measurement
☒	☐	A statement on whether measurements were taken from distinct samples or whether the same sample was measured repeatedly
☒	☐	The statistical test(s) used AND whether they are one- or two-sided<br>*Only common tests should be described solely by name; describe more complex techniques in the Methods section.*
☒	☐	A description of all covariates tested
☒	☐	A description of any assumptions or corrections, such as tests of normality and adjustment for multiple comparisons
☐	☒	A full description of the statistical parameters including central tendency (e.g. means) or other basic estimates (e.g. regression coefficient) AND variation (e.g. standard deviation) or associated estimates of uncertainty (e.g. confidence intervals)
☒	☐	For null hypothesis testing, the test statistic (e.g. *F*, *t*, *r*) with confidence intervals, effect sizes, degrees of freedom and *P* value noted<br>*Give P values as exact values whenever suitable.*
☒	☐	For Bayesian analysis, information on the choice of priors and Markov chain Monte Carlo settings
☒	☐	For hierarchical and complex designs, identification of the appropriate level for tests and full reporting of outcomes
☒	☐	Estimates of effect sizes (e.g. Cohen's *d*, Pearson's *r*), indicating how they were calculated

Our web collection on statistics for biologists contains articles on many of the points above.

Software and code

Policy information about availability of computer code

Data collection	We used Illumina pipelines for image processing and base calling to collect our data.
Data analysis	BWA (version 0.7): Map Hi-C, Micro-C and Chip-seq data to mm10;<br>Pairtools (version v0.3.0) : Generate valid pairs for Hi-C and Micro-C data;<br>Cooler (version 0.8.11): Build balanced interaction matrices for Hi-C and Micro-C data;<br>MACS2 (version 2.2.7.1): Call peaks from Chip-seq data;<br>FIMO from MEME suite (version 5.4.1): Call CTCF motifs from Chip-seq targeting CTCF;<br>Cooltools (version 0.5.4): Calculate contact probability as a function of genomic distance, perform eigenvector decomposition, call TADs from Hi-C and Micro-C data and visualize Hi-C and Micro-C contact matrices;<br>Chromosight (version 1.6.3): Call loops from Micro-C data;<br>Hicrep (0.2.6): Assess reproducibility of Hi-C and Micro-C data;<br>coolpuppy (version 1.0.0): Perform aggregation of interactions;<br>R package were used to perform downstream analyses and plot figures.<br>Adobe Illustrator 2025: Draw schematics.

For manuscripts utilizing custom algorithms or software that are central to the research but not yet described in published literature, software must be made available to editors and reviewers. We strongly encourage code deposition in a community repository (e.g. GitHub). See the Nature Portfolio guidelines for submitting code & software for further information.

Data

Policy information about availability of data

All manuscripts must include a data availability statement. This statement should provide the following information, where applicable:

- Accession codes, unique identifiers, or web links for publicly available datasets
- A description of any restrictions on data availability
- For clinical datasets or third party data, please ensure that the statement adheres to our policy

All data has been deposited in the GEO: GSE288838, GEO: GSE288839 and GEO: GSE288837.

Research involving human participants, their data, or biological material

Policy information about studies with human participants or human data. See also policy information about sex, gender (identity/presentation), and sexual orientation and race, ethnicity and racism.

Reporting on sex and gender	*Use the terms sex (biological attribute) and gender (shaped by social and cultural circumstances) carefully in order to avoid confusing both terms. Indicate if findings apply to only one sex or gender; describe whether sex and gender were considered in study design; whether sex and/or gender was determined based on self-reporting or assigned and methods used. Provide in the source data disaggregated sex and gender data, where this information has been collected, and if consent has been obtained for sharing of individual-level data; provide overall numbers in this Reporting Summary. Please state if this information has not been collected. Report sex- and gender-based analyses where performed, justify reasons for lack of sex- and gender-based analysis.*
Reporting on race, ethnicity, or other socially relevant groupings	*Please specify the socially constructed or socially relevant categorization variable(s) used in your manuscript and explain why they were used. Please note that such variables should not be used as proxies for other socially constructed/relevant variables (for example, race or ethnicity should not be used as a proxy for socioeconomic status). Provide clear definitions of the relevant terms used, how they were provided (by the participants/respondents, the researchers, or third parties), and the method(s) used to classify people into the different categories (e.g. self-report, census or administrative data, social media data, etc.) Please provide details about how you controlled for confounding variables in your analyses.*
Population characteristics	*Describe the covariate-relevant population characteristics of the human research participants (e.g. age, genotypic information, past and current diagnosis and treatment categories). If you filled out the behavioural & social sciences study design questions and have nothing to add here, write "See above."*
Recruitment	*Describe how participants were recruited. Outline any potential self-selection bias or other biases that may be present and how these are likely to impact results.*
Ethics oversight	*Identify the organization(s) that approved the study protocol.*

Note that full information on the approval of the study protocol must also be provided in the manuscript.

Field-specific reporting

Please select the one below that is the best fit for your research. If you are not sure, read the appropriate sections before making your selection.

☒ Life sciences ☐ Behavioural & social sciences ☐ Ecological, evolutionary & environmental sciences

For a reference copy of the document with all sections, see nature.com/documents/nr-reporting-summary-flat.pdf

Life sciences study design

All studies must disclose on these points even when the disclosure is negative.

Sample size	For each Hi-C or Micro-C experiment, independent biological replicates were performed. In each replicate, nuclei from 2–5 mice were pooled prior to sorting, and 500,000–1,000,000 nuclei were collected per population. Sample sizes were chosen based on prior studies and practical considerations; no formal statistical methods were used to predetermine sample size.
Data exclusions	The details and the rationale of data exclusion for specific bioinformatic analyses have been described in Methods. Y chromosome is mostly blank on the contact map, which was excluded from analysis in this paper. Interactions features disappear during pachynema ue to MSCI (Meiotic Sex Chromosome Inactivation), which was also excluded from analysis in this study.
Replication	For Hi-C and Micro-C experiments, reproducibility between independent biological replicates was assessed using the HiCRep package (v0.2.6) (Supplementary Fig. 2). For CTCF ChIP-seq, reproducibility between two biological replicates was evaluated by calculating the percentage of overlapping peaks.
Randomization	Randomization was not performed for sorting neuclei, performing Hi-C, Micro-C or CTCF Chip-seq. A random bed file with random genomic regions was generated by bedtools for pari-wise aggregation analysis (Supplementary Fig. 17 and Supplementary Fig. 18).

Blinding	Blinding was not performed during data collection.

Reporting for specific materials, systems and methods

We require information from authors about some types of materials, experimental systems and methods used in many studies. Here, indicate whether each material, system or method listed is relevant to your study. If you are not sure if a list item applies to your research, read the appropriate section before selecting a response.

Materials & experimental systems

n/a	Involved in the study
☐	☒ Antibodies
☒	☐ Eukaryotic cell lines
☒	☐ Palaeontology and archaeology
☐	☒ Animals and other organisms
☒	☐ Clinical data
☒	☐ Dual use research of concern
☒	☐ Plants

Methods

n/a	Involved in the study
☐	☒ ChIP-seq
☐	☒ Flow cytometry
☒	☐ MRI-based neuroimaging

Antibodies

Antibodies used

1. PLZF Antibody (D-9) FITC; supplier name: Santa Cruz Biotechnology; catalog number: sc-28319 FITC; clone name: D-9; lot number: G2417. Dilution: 1μg/10 million cells in 300 μl solution.
2. DMRT1 Antibody (A-9) Alexa Fluor® 647; supplier name: Santa Cruz Biotechnology; catalog number: sc-377167 AF647; clone name: A-9; lot number: F2422. Dilution: 1μg/10 million cells in 300 μl solution.
3. SCP-3 Antibody (D-1) Alexa Fluor® 488; supplier names: Santa Cruz Biotechnology; catalog number: sc-74569 AF488; clone name: D-1; ot number: H2625. Dilution: 1μg/10 million cells in 300 μl solution.
4. Anti-Stra8; supplier name: abcam; catalog number: ab49602; lot number: 1074434-1. Dilution: 1μg/10 million cells in 300 μl solution.
5. Anti-H1t; a custom-made antibody from Neo-Bioscience. Dilution: 1μg/10 million cells in 300 μl solution.
6. SCP1 Antibody [Alexa Fluor® 647]; supplier name: Novus Biologicals; catalog number: NB300-299AF647; lot number: D170229. Dilution: 1μg/10 million cells in 300 μl solution.
7. Anti-Rec8; custom-made antibody. Dilution: 1:200.
8. Anti-Rad21L; a gift from Miguel Brieño-Enriquez; Dilution: 1μg/10 million cells in 300 μl solution.
9. Anti-Rad21; supplier name: Abcam; catalog number: ab217678; lot number: GR3362682-12. Dilution: 1μg/10 million cells in 300 μl solution for immunostaining. Dilution: 1:2000 for Western Blot.
10. Anti-CTCF: supplier name: Abcam; catalog number: ab128873, lot number: 1007248-15.Dilution: 1:1500 for Co-IP
11. Anti-CTCF: supplier name: Abcam; catalog number: ab70303, lot number: GR3281212-12: 5 μg antibody was used to perform Chip-seq for one mouse.
12. Normal rabbit IgG: supplier name:Upstate; catalog number: 12-370; ot number: JBC1356564. Dilution: 1:1500 for Co-IP
13. Anti-REC8: supplier name: Abcam; catalog number: ab192241, lot number: GR3254930-5. Dilution: 1:2000
14. Anti-TATA binding protein TBP antibody: supplier name: Abcam; catalog number: ab63766, lot number: GR3430989-3. Dilution: 1:2000.
15. Anti-FLAG: supplier name: Millipore-Sigma. catalog number: F1804, lot number: 100336729. Dilution: 1:2000.

Validation

1. PLZF Antibody (D-9) is a mouse monoclonal IgG1 antibody, cited in 189 publications. We used this antibody conjugated with FITC as a marker for nuclei sorting.
2. DMRT1 Antibody (A-9) is a mouse monoclonal IgG1 antibody, cited in 13 publications. We used this antibody conjugated with AF647 as a marker for nuclei sorting.
3. SCP-3 Antibody (D-1) is a mouse monoclonal IgG1 ntibody, cited in 154 publications. We used this antibody conjugated with AF488 as a marker for nuclei sorting.
4. Anti-Stra8 antibody (ab49602) is a rabbit polyclonal antibody detecting Stra8 in Western Blot, IHC-P. Suitable for Mouse. This antibody has been used in over 80 publications according to the manufacturer's website. We used this antibody as a marker for nuclei sorting.
5. The custom-made H1t antibody is a rabbitpolyclonal IgG. It was validated using immunofluorescence staining in spermatocyte spreads. This antibody has been used in a previously published study (Kwan-Wood Gabriel Lam et al., 2019).
6. SCP1 Antibody is polyclonal rabbit IgG. 61 publications using this antibody has been listed on the manufacturer's website. We used a conjugated antibody as a marker for nuclei sorting. We used this antibody conjugated with AF647 as a marker for nuclei sorting.
7. Anti-Rec8; This custom-made antibody was validated using immunofluorescence staining in spermatocyte spreads. This antibody was used to profile changes in signal density during pre-meiotic development by fluorescence-activated cell sorting (FACS).
8. Anti-Rad21L; A study using this antibody has been published previously (Miguel A. Brieño-Enríquez et al., 2016). This antibody was used to profile changes in signal density during pre-meiotic development by fluorescence-activated cell sorting (FACS).
9. Anti-Rad21; ab217678 is a rabbit monoclonal antibody. 17 publications using this antibody has been listed on the manufacturer's website. This antibody was used to profile changes in signal density during pre-meiotic development by fluorescence-activated cell sorting (FACS) and Western Blot.
10. Anti-CTCF (ab128873); This is a Chip-grade antibody and has been used in 42 publications. We use this antibody to perform immunofluorescence staining in spermatocyte spreads and co-immunoprecipitation.

	11. Anti-CTCF (ab70303); Anti-CTCF antibody (ab70303) is a rabbit polyclonal antibody detecting CTCF in Western Blot, IP, IHC-P. Suitable for Human, Mouse. This antibody has been referenced in 100 publications according to the manufacturer's website. We use this antibody to perform Chip-seq.<br>12. Normal rabbit IgG. Upstate (12-370) has been cited in 1,060 publications, with 23 published images.<br>13. Anti-REC8: (ab192241). This antibody has been cited in 14 publications.<br>14. Anti-TATA binding protein TBP antibody (ab63766) has been cited in 49 publications.<br>15. Anti-FLAG (F1804) has been cited in 11,674 publications.<br>.

Animals and other research organisms

Policy information about studies involving animals; ARRIVE guidelines recommended for reporting animal research, and Sex and Gender in Research

Laboratory animals	C57BL/6J (Stock no. 000664) and CAST/EiJ (Stock no. 000928) were purchased from The Jackson Laboratory. The F1 hybrids were in-house bred with male CAST/EiJ with female C57BL/6J. Mice with age around 8 to 26 weeks were used. Mice were maintained in a controlled environment with a 12-h light/dark cycle, temperature of 22±2°C, and 50–60% humidity.
Wild animals	We do not use wild animals in this study
Reporting on sex	This study focused on spermatogenesis, so that only males were used for this study.
Field-collected samples	This study did not involve samples collected from the field
Ethics oversight	Mice were sacrificed in accordance with the NIH Animal Care and Use regulations.

Note that full information on the approval of the study protocol must also be provided in the manuscript.

Plants

Seed stocks	*Report on the source of all seed stocks or other plant material used. If applicable, state the seed stock centre and catalogue number. If plant specimens were collected from the field, describe the collection location, date and sampling procedures.*
Novel plant genotypes	*Describe the methods by which all novel plant genotypes were produced. This includes those generated by transgenic approaches, gene editing, chemical/radiation-based mutagenesis and hybridization. For transgenic lines, describe the transformation method, the number of independent lines analyzed and the generation upon which experiments were performed. For gene-edited lines, describe the editor used, the endogenous sequence targeted for editing, the targeting guide RNA sequence (if applicable) and how the editor was applied.*
Authentication	*Describe any authentication procedures for each seed stock used or novel genotype generated. Describe any experiments used to assess the effect of a mutation and, where applicable, how potential secondary effects (e.g. second site T-DNA insertions, mosiacism, off-target gene editing) were examined.*

ChIP-seq

Data deposition

☒ Confirm that both raw and final processed data have been deposited in a public database such as GEO.

☒ Confirm that you have deposited or provided access to graph files (e.g. BED files) for the called peaks.

Data access links<br>*May remain private before publication.*	https://www.ncbi.nlm.nih.gov/geo/query/acc.cgi?acc=GSE288837
Files in database submission	GSM8776361 CTCF ChIP-seq from mouse testes rep 1<br>GSM8776362 CTCF ChIP-seq from mouse testes rep 2<br>GSM8776363 imput sample from mouse testes rep 1<br>GSM8776364 imput sample from mouse testes rep 2<br>GSE288837_Chip.CTCF.BXC.wholeTestis.mm10.merged_peaks.narrowPeak.gz
Genome browser session<br>(e.g. UCSC)	*Provide a link to an anonymized genome browser session for "Initial submission" and "Revised version" documents only, to enable peer review. Write "no longer applicable" for "Final submission" documents.*

Methodology

Replicates	Two biological replicates were perfomed for CTCF Chip-seq
Sequencing depth	CTCF ChIP-seq from mouse testes rep 1: pair-end 150 bp sequencing. total reads 144,760,956. Unique mapped reads: 126,522,013<br>CTCF ChIP-seq from mouse testes rep 2: pair-end 150 bp sequencing. total reads 189,431,113. Unique mapped reads: 158,899,515<br>Imput sample from mouse testes rep 1: pair-end 150 bp sequencing. total reads 74,660,254. Unique mapped reads: 66,356,201<br>Imput sample from mouse testes rep 2: pair-end 150 bp sequencing. total reads 84,532,268. Unique mapped reads: 73,667,734
Antibodies	The CTCF Chip-seq was performed with ab70303 from Abcam. lot number: GR3281212-12

Peak calling parameters	Bam files from two CTCF Chip-seq were merged; Bam files from two input samples were merged. Peaks were called using MACS2 (version 2.2.7.1) with qvalue cutoff = 0.05 with input as a control.
Data quality	51871 peaks were called with FDR 0.05. 42468 peaks contain at least on CTCF motif based on FIMO analysis
Software	MACS2 (version 2.2.7.1) was used to call peaks. FIMO from MEME suite (version 5.4.1) with JASPAR PWM MA0139 was used to identify CTCF motifs.

Flow Cytometry

Plots

Confirm that:

- [x] The axis labels state the marker and fluorochrome used (e.g. CD4-FITC).
- [x] The axis scales are clearly visible. Include numbers along axes only for bottom left plot of group (a 'group' is an analysis of identical markers).
- [x] All plots are contour plots with outliers or pseudocolor plots.
- [ ] A numerical value for number of cells or percentage (with statistics) is provided.

Methodology

Sample preparation	Mice were euthanized, and testes were retrieved. For Hi-C, the testes were fixed with 1% formaldehyde at room temperature for 10 minutes. For Micro-C, the testes were fixed with 1% formaldehyde at room temperature for 10 minutes followed by 3 mM disuccinimidyl glutarate (DSG) for an additional 45 minutes at room temperature. Fixation was quenched with glycine (final concentration 125 mM). The fixed testes were homogenized with at least 10 strokes in a Dounce homogenizer and filtered by passing through a 70 µm cell strainer. The cell suspension was washed once with 1X PBS (phosphate buffered saline) and then resuspended in nucleus extraction buffer (15 mM Tris-HCl pH 7.4, 0.34 M sucrose, 15 mM NaCl, 60 mM KCl, 0.2 mM EDTA (Ethylenediaminetetraacetic acid), 0.2 mM EGTA (ethylene glycol tetraacetic acid). Nuclei were extracted using a Dounce homogenizer with 30 strokes of tight pestle, repeated once. Nuclei were filtered through a 40 µm cell strainer and resuspended in a chilled PBTB buffer (1× PBS with 0.1% Triton X-100, 5% bovine serum albumin, and protease inhibitor). The nuclei were incubated with primary antibodies at room temperature for 1 hour or 4 °C overnight. They were then washed with PBTB and labeled with secondary antibodies at room temperature for 30 minutes. After washing twice with PBTB, nuclei were filtered with a 40 µm cell strainer and stained with DAPI (4′,6-diamidino-2-phenylindole) for sorting.
Instrument	BD FACSymphony™ S6
Software	FlowJo v10 was used for flow cytometric data analysis. R package flowCore and ggplot2 were used to plot the figures.
Cell population abundance	An estimated purity is between 85% to 95%.
Gating strategy	For collecting populations from undifferentiated spermatogonia to meiotic G1: Singlets were gated using both SSC and FSC gate. From 2C populated gated by DAPI signal, were identified as 2C nuclei expressing the PLZF; Differentiating spermatogonia were gated as DMRT1 strong and STRA8 negative; Transition I to meiotic G1 were gated as populations with continue decrease in DMRT1 and increase in STRA8.<br>For collecting meiotic S and leptonema: Singlets were gated using both SSC and FSC gate. 2-4C and 4C nuclei were gated based on DAPI signals. Meiotic S cells were gated as 2-4C nuclei with strong STRA8, low SCP3. Leptonema were gated as 4C nuclei with strong STRA8 and strong SCP3.<br>For sub-stages during meiosis I prophase. Singlets were gated using both SSC and FSC gate. 4C nuclei were gated based on DAPI signal. Zyotene to diplotene nuclei were gated based their increase in H1t and first increase then decrease in SCP1

- [x] Tick this box to confirm that a figure exemplifying the gating strategy is provided in the Supplementary Information.
