# Supplementary material for: High-resolution chromatin mapping reveals that CTCF anchors meiotic loops to the chromosome axis
Source: Nat Commun. 2026 Jul 28;17:7550. doi: 10.1038/s41467-026-73644-6 (PMC13416154; doi:10.1038/s41467-026-73644-6)
Supplement: Supplementary file 2 — Transparent Peer Review file [file 41467_2026_73644_MOESM2_ESM.pdf]

nature portfolio

Peer Review File

High-resolution chromatin mapping reveals that CTCF anchors meiotic loops to the chromosome axis

Corresponding Author: Dr R. Daniel Camerini-Otero

This file contains all reviewer reports in order by version, followed by all author rebuttals in order by version.

Version 0:

Reviewer comments:

Reviewer #1

(Remarks to the Author)
This is a very important and interesting paper. It applies the latest chromosome capture methodologies to highly synchronous populations of mouse spermatocytes to provide unprecedented spatio-temporal resolution of "chromosome folding" during meiosis. It presents a large number of interesting and new findings which I was very happy to read about.

I have made quite a few comments below. I would like to emphasize that this is not a reflection on the quality of the work, which is outstanding, or the importance of the conclusions, which are substantial. Rather, it is because the data and conclusions are complex and the presentation and integration of those findings is sometimes challenging. I would like to see things even cleaner in presentation than they are at present. Also, if this were my work, I would place more emphasis on certain things (with which the authors may not agree, or maybe they can take this into account). Basically, I'm hoping that these comments will help to make the revised version of the paper better. I hope the authors are not too unhappy with these inputs.

For this reviewer, the following key points are important:
1. AB domains disappear during meiosis. This is clear in the current findings; makes obvious sense given that meiotic chromosomes are organized in cooriented sister linear loop arrays. It corrects findings to the contrary in the literature which the authors demonstrate can be explained by impurity of samples in previous work. (The presence of the sister and the homolog needs to be remembered; point 4 below).
2. TADs disappear during meiosis. This has been shown previously and it is apparent in the current work and shown very cleanly.
3. A key issue in the meiosis field is loop sizes. The P(s) curves suggest that loop sizes increase with time up to/through pachytene. The authors have avoided showing later stages, perhaps because there is an apparent discrepancy between cytology and HiC conclusions. But this is not the correct approach; these results should be included in the text. The results presented in this paper provide pretty good evidence that the situation is that loop sizes go from 100kb to 200kb to 600kb. This comes not only from P(s) curves (interpretation of which depends on modeling, as the authors point out, but from the independent analysis of distances between loop base contacts as presented in this work, which is a very important addition to the situation). Cytology estimated 100-200kb loops. But the result here is validated by the fact that when the appropriate loop size is used, the density of loops now correctly matches the "evolutionarily conserved" density seen across many organisms. As pointed out by deMassy (Frontiers review), cytological estimates have to assume a particular packing ratio of the chromatin fiber, which is not known. If the assumed ratio was too low, the loop size will be too short, as is apparently the case. The findings in this paper allow a satisfying resolution to this issue, at least as a sensible replacement for current confusion. It would be useful to know how this compares with loop sizes along mitotic prophase and prometaphase chromosomes (see discussion of stages below). Should be the same as mitotic prophase and less than prometaphase.
4. Meiotic chromosomes are known from cytological studies to comprise loops emanating from a complex structural axis meshwork. Pevious work failed to detect signals that might represent loop base contacts. The present work detects such signals as "dots" which do not occur at the tips of TAD triangle signals. This is very important, in addition to allowing an independent assessment of loop sizes (above), for two reasons.
- The data allow the authors to suggest that loops do not form by loop extrusion along meiotic chromosomes. Their reasoning and intuition seem correct. Several points about this conclusion could be mentioned. The authors discuss their findings by saying that loops are "dynamic" in mitotic chromosomes (interphase) and "stable" in meiotic prophase. "Stability" is not measured directly but is inferred from the strength of the signal; this could be emphasized more.

The issue of meiotic vs mitotic prophase is also important in this context, as discussed below; this may not be meiosis-specific.
It is clear that there is an important issue: interphase mitotic loops are dynamic/transient whereas the prophase structure is morphologically stable. The same stability may well be true at mitotic prophase. However, it is also important that adjacent loops are sort of stochastic in nature in interphase but have a very specific spacing along meiotic prophase chromosomes. Packing of adjacent loops side-by-side is a different point from stability. In principle, a morphologically constant array could arise with subunits coming and going. In fact, such a situation is known for the meiotic SC (C.elegans work). And it is known (although I am not sure it is published) that meiotic worm chromosome axes do NOT show this same behavior. So it is clear that there is also a stability difference, but not directly from the current work. Regardless, the observed patterns do strongly argue against loop extrusion as a mechanism and instead match a recent model from mitotic chromosomes (which the authors discuss) in which loop arrays form by progressive extension. Their discussion of this point is spot on correct. The differences in CTCF patterns are interesting and important and novel. The authors may also wish to appreciate a model (1999) in which meiotic prophase loops occur by collisions, with loop size determined by chromatin fiber persistence length; this could still be possible. Whether it is progressive along chromosomes or not is another issue....Outcome is a series of adjacent modules, but whether they are progressive in time is a different point. Interestingly, unpublished data from Franz Klein could argue against extrusion for meiotic prophase in yeast (they could ask him about this).
To illustrate the above issues, in the Discussion, line 403: "Our data cannot formally distinguish between active loop extrusion or multiple loops forming statically and then fusing. We favor the hypothesis of loops forming statically rather than dynamically due to the presence of corner dots without TADs." NB conflation of loop formation mechanism vs loop "stability"...needs to be clarified in language.
- The detection of loop base contacts leads, along with other data, to further analysis of CTCF/Rec8. That analysis is very useful and important information. The authors are more excited about the molecules than this reviewer, who thinks that the reallly important point is the implication for how loops form.
Thus, as a secondary point: in presenting these findings, the authors tend conflate detection of loop base contacts with analyses of CTCF/Rec8. These two issues should be more separated. Detection of these contacts is important regardless of what molecules are involved. In this regard, also, the authors make the factually correct statement that loop base contacts were observed in yeast but not in mammals. This is true, and the fact that these contacts are detected in the present has yielded alot of new information about molecules and about the nature of loop formation, all of which is seminal and important. But it can be noted that the explanation for the difference in the findings in the two organisms has a deeper reason. In yeast, the number of loops is very close to the number of potential axis association sites. So in a total population, all combinations of loop possibilities (and thus dots) are visible. In a mamalian cell (with longer loops, e.g.) there are many more loop possibilities and thus dots will be fainter. If I understand correctly, tyhis is why the current analysis is, for the first time, technically able to detect these contacts. The authors focus on Rec8 or not Rec8, but this isn't really the issue maybe...... I think that is not the reason for the failure to detect contacts in mammals previously. The current study has the resolving power required.
5. On established chromosomes, CTCF is the important player, versus Rec8. Aside additional implications, this is an important general finding. The difference with yeast is important to note although, of course, yeast has to make do with fewer molecules and doesn't have CTCF, so the same principles likely apply nonetheless.
Also: is it possible that loops are formed by Rec8 seeing e.g. locally AT-rich regions with CTCF then seeing the same positions concomitantly? This might be an alternative explanation for the absence of convergent CTCF patterns? (although I like the authors' explanation). See DOI:
10.1093/nar/gkaf587.
Another issue that might be addressed: is it possible that the HiC axis dots are in some way an artifact? In a closely packed situation, a certain genomic region can contact any region nearby, which may mask specific patterns like TADs. Additionally, fixation and ligation may be highly biased towards a dense protein/DNA network along chromosome axes, which potentially causes “artificial” contacts. This does not seem likely, given the resulting loop size implication, and especially as meiotic chromosomes are not SO compact (unlike mitotic prometaphase chromosomes) - rather they are just well-organized. Perhaps this could be discussed a bit.
6. The authors emphasize the nature of the transition into meiosis. These findings are an important addition to understanding. They find that detectable changes occur very early and they argue that an "unstructured" intermediate (Transition I) is important. This is all well-documented. However, there are several points that could be discussed/clarified.
- Is it known, when cells enter Transition I, whether they do so from a particular stage of the preceding/ongoing mitotic cell cycle? The authors seem to think they are entering from interphase, but maybe they are entering from telophase of the prior cycle? or? And are there divisions going on during this transition or ??
- The authors argue for a two-stage progression. Dekker has published that there is a two-stage progression in the telophase/G1 transition of mitotic cells. It would be useful to compare the two cases. They could be functionally analogous in the sense that a first stage allow entropy-driven decatenation and the second allows reorganization without recatenation.
7. DSB/recombination sites colocalize with the loop base contacts. This is useful and important, and is strong validation of the identification of axis association sites, but is not new/surprising, given long history of "tethered loop axis complexes". Right? Also
a. The authors make the statement that this is progressively lost with time as repair proceeds (line???). This is not very precise. Crossover interactions remain associated until diplotene. Noncrossover interactions are released from the axis at early pachytene (Ogawa and general knowledge). Result is reduced localization of recombination complexes to axes. This point could be usefully made to explain their result, since it provides evidence for such release.
b. The conclusion (Abstract) that " loops at defined sequences might be an important feature to keep homologous chromosomes in register" seems to me problematic. It is unclear that exactly the same loop bases are used along every chromosome as required by this statement??? This should be addressed. Maybe the authors think "yes" because their loop size calculations, based on this assumption, give the right answer? Also, the authors need to consider the fact that even sister chromatids are not aligned in register (Gerlich for mitosis, worm data for meiosis). Different loop bases are chosen in

different sisters - the issue is a structural match of loop base modules, seen cytologically as banded lateral elements by EM - not a specific match at the DNA level. The authors have alot of very interesting findings (above) but seem here to be reaching for sexier conclusions when that is not necessary. Implications for other things is more than sufficient to support the importance of this study.
8. Interactions relating to gene expression are largely conserved. This is sensible given that gene expression must go on, and important to have shown. However, the authors have framed this as a "mitosis vs meiosis" issue on the presumption that meiosis is unique. But this is probably not true. As described below, meiotic prophase is analogous to mitotic prophase, not mitotic prometaphase (which is what the authors consider "mitosis"; further comments below). It would be better to discuss this in a broader way. Clearly it is important for meiotic prophase chromosomes to continue transcription. Given that loops are 600kb, with chromatin undergoing dynamic changes in diffuseness/compaction (below), it is perfectly reasonable that transcriptional links are still present. But there needs to be a discussion of the length scales of the examined contacts, relative to loop size (and maybe even relative to loop base positions?). Again, this is very important information - it just needs to be discussed a bit differently, according to me, at least. The authors emphasize: (Line 338) "Our data revealed a unique global chromatin organization that results from balancing between the dense linear loop array configuration and maintaining active transcription.". This seems to me to be a bit of a straw man. Why should an array of 600kb loops preclude transcription? No need to over-hype this point, especially since contrast they make is with mitotic prometaphase, which is an entirely different issue.

General comments. There are some general issues that could be better addressed.
9. It might be useful to start with a better description of what is known about meiotic chromosome organization, rather than an extensive discussion of mitotic chromosomes which, for this reviewer, is not so interesting and which does not necessarily have the relevance that the authors think (see point 2 below)
10. The term "compaction" is not accurate and should be avoided. This is a general problem in the mitotic and meiotic fields. One difference between "interphase" and "later stages" is that the chromosomes are organized and individualized ("structured" in these authors' terms). A second issue is whether the state of the chromatin fiber is altered. In mitotic prometaphase chromosomes, there is also chromatin compaction as well as organization (e.g. Gerlich). In meiosis, it has been shown, albeit without extensive documentation, that chromatin alternates between more and less diffuse states (so-called "expansion/compaction cycles" (Kleckner, 2004, 2006; Zickler and Kleckner 2023). This is important generally. It is also important because some of the stage-specific effects that the authors report here could reflect these changes: expansion at leptotene, compaction and zygotene, expansion (maybe with complexities) at pachytene, and compaction at diplotene. This might be particularly true for microC results.
11. The authors compare "meiosis" (meaning meiotic prophase" to "mitotic chromosomes" (meaning mitotic prometaphase), with differences attributed to special features of meiosis. However, this is not a correct view of the mitotic situation. Mitotic chromosomes go through a prophase stage which is, as far as can be told, the same as meiotic prophase. This is shown by Liang et al., 2015. It is exemplified by the fact that, in muntjac, the lengths of meiotic SCs are identical to the lengths of chromosomes trapped in prophase by TopoII inhibition. In the authors' defense: the HiC-related community fails to understand/accept this relationship because it is a transient stage and requires single cell imaging to see; it is missed or otherwise avoided in standard synchronized HiC experiments. This is important, because the HiC community also recognizes that they don't understand mitotic prophase (although perhaps recent studies of which I am unaware address this point) and because it may well be that the features seen here for meiotic phase are not meiosis-specific but rather are "prophase-specific". Both possibilities need to be discussed.
12. More generally, this reviewer would prefer to see the authors simply describe everything they see and THEN go back to (a) explaining previous meiotic results and why they were right/wrong; and (b) providing comparisons to mitotic prometaphase chromosomes with the above in mind. These results stand on their own and need to be presented and interpreted and understood on their own. The rest can follow.
13. The authors assume that all of the special features that they detect are due to organization along individual chromosomes. But there are several caveats. The most important is that this is meiosis and so there are homologs and it is not excluded that there are interactions between homologs which feed into some of the findings. This is not certain, but as the authors know, HiC can't tell the difference. MicroC might be too short range for this to matter. Homologs may interact specifically by pairing, and they might interact without directly pairing due to architectural features that tend to coalign them anyway (centromere/telomere pairing?) at early stages particularly. By pachytene, homolog chromatin probably lies on opposite sides of the SC mostly; at coalignment, this may mostly be true but some contacts are possible given that chromatin is not Some comment about this would be helpful. Additionally, of course, there are interactions between sister chromatids. (And then there are chromatin expansion/compaction variations; above).
14. Different chromosomes are shown for different points. Explain why and which things are/are not chromosome specific? Maybe this is in the SI?

Abstract. Overall, the authors have made many important and interesting findings. It is difficult to corral them around one or a few major points.....They can improve in this respect. As a general point, because there is so much important information, it is difficult to write an abstract that gives the major message. But more effort should be made in this regard, with less of a shopping list of outputs.
Also, the points emphasized seem to come from a "mitotic" perspective which, for this reviewer, is less interesting. The findings are really important for meiosis qua meiosis.
Generally speaking, as stated elsewhere in this review - given 600kb loops that vary in diffuseness, it is hardly surprising that very local interactions are maintained and transcription can continue. Probably active genes are in loops? This reviewer is less interested in such issues than in what these findings say for meiosis, which is really important and is the main subject. That being said, the ES cell/meiosis comparison is useful.
14. See point re "condense" above.
15. "local subcompartments...are maintained". This should be stated another way. Local subcompartments are DETECTED,

not "maintained", unless they are referring to very local interactions. This is unclear to people thinking of loop/axis arrays. More generally, the Calder analysis in Figure 1 seems disconnected from subsequent findings and is sort of a HiC-specific issue....Perhaps I am not understanding the significance (Discussion lines 329ff?). That section also says: local compartments are more visible in meiosis due to changes of cohesin activities. Well, really, the point is that local compartments are more visible because the linear loop array is more regular and the noise from random TADs and AB domains is gone. What molecules are involved is another issue.
16. "challenging the current view that loops are positioned randomly along axial elements". See above: failure to detect specific loop bases is not the same as "positioned randomly" which is clearly silly. This seems an overstatement. Put the result positively - for the first time can detect loop base linkages and this leads to reconciliation of loop size issues and interesting new suggestions for how loops form along meiotic prophase chromosomes.

Results. Perhaps I have not fully understood, but these are points to consider clarifying?
17. Figure 1. There could be more discussion of the details of the analyses.
a. One general point is that PCA and Calder reveal the presence of organizational features, but do not do a very good job of capturing the overall situation as defined by the intensities of the primary signals, which seem to tell an additional story, if I understand correctly. Irrespective of PCS and Calder, it is obvious that defined patternrs decrease at S-leptotene transition. Notably, it is known that meiotic chromosome organization develops after S and before leptotene, so during this transition, so this data fits with what is known. This should be pointed out. Some data are in recent yeast paper by Nozaki et al in Nature. Plus it is obvious that there is not just a general smear of contacts after that point, so something remains. What those remaining contacts are is studied further.
b. What is the point of the Calder analysis? Does it have a specific organizational meaning? Is it a reflection of features described later? This should be clarified. I didn't find it helpful, although of course it has to be included. Or maybe I am missing something important.
c. There are complexities within PCA signals that seem interesting and should be discussed if they are meaningful. Obviously AB compartments start to go way at S/Leptotene transition, which is sensible (above). NB that events along chromosomes occur asynchronously through the nucleus, so "leptotene" includes some regions that have done coalignment and some that have not as well as some that maybe haven't developed structure. More importantly, at zygotene, there is some SC, but alot of regions haven't even done coalignment. In any case, linear loop arrays are developed by leptotene. So why are there changes in AB scores after that point? Are they meaningful? Is there something special about middles vs ends of chromosomes? Do any of these effects reflect centromere/telomere/end clustering? Changes in chromatin compaction? At diplotene, are there B components only at the centomere end of the chromosome? At later stages, in microC, the general background gets much fuzzier. Why is that? This analysis may be especially sensitive to expansion/compaction of chromatin? Why are there Calder contacts only in B domains (green)? What is going on with PCA data from zygotene to diplotene? Are these changes meaningful?
d. Panel D is not understandable to the non-expert and Panel is not useful, particularly because Calder analysis is ambiguous. There could/should be a proper summary cartoon of what happens at various stages in the Discusion.

18. Figure 2.
a. Panel B - it is hard to see the patterns under the grey lines. Remove them from most panels or make light/dotted.
b. The insulation score and its significance is never discussed. For the non-expert it would be useful. Is it basically the prominence of the TAD? or its width? or ??

19. Figure 3.
a. The data for later stages should be in the text and properly discussed.
b. "Loop size" (as the authors point out) is a HiC computation feature. It should be called "simulated loop size". It can be mentioned that results are/are not confirmed by analysis of loop bases below.
c. Save Panel G for the Discussion.

20. Figure 4.
a. Panel a was confusing. Legend does not explain mES cell portion. it should be more obvious. And what are we to conclude about positions of black arrow dots in mES cells? Are they there or not there?
21. Figure 5.
"stablizing loops". What is measured is the steady state level of loops as given by intensities of signals. This should be made clear. Stability is not directly measured but inferred from higher steady state levels.
22. Figure 6. Move panel F to Discussion? Overall, Discussion needs to give a synthetic picture of what happens in meiosis at various levels, from their data.....

Discussion. Most of my comments are given above. One small point - line 353. "Interestingly, we observe higher insulation between genes at pachynema and diplonema. This phenomenon could potentially be the result of a significant accumulation of “sticky” RNA Pol II on genes at these stages and the increased transcriptional activity." Huh? How about changes in compaction status? In any case, this is not really a big point compared to other finding. Maybe omit?

I realized that there is a further issue regarding dots not at corners of TADs. The authors estimation of loop size assumes that same positions are used in all nuclei. The estimate matches their HiC P(s) curves and gives appropriate inter-loop spacing (20/micron axis). But using exactly the same positions in all cases is not, a priori, so likely (see comments re yeast). It would be worth discussing what must be said for this to be the case. Perhaps the authors did this, and it is part of their idea that loops are phased on homologs. But do they understand the basis for it? Maybe I missed this point, but thought I would bring it up in case further discussion would be useful.

Reviewer #2

(Remarks to the Author)
Cheng et al. performed high-resolution chromatin mapping across stages preceding and during mouse meiotic prophase I in males, using both Hi-C and Micro-C. While their study follows a wave of publications applying Hi-C to examine spermatogonia and meiotic prophase, it stands out for the exceptional quality of their data and fine temporal resolution. This level of detail allowed the authors to uncover several findings that diverge from, or challenge, previously reported views of chromatin organization during mammalian meiosis greatly clarifying and correcting prior models. Their key findings include:

Loss of large-scale A/B compartments during prophase I, contrasting with most prior studies (with the exception of Vara et al.) that reported the persistence of typical A/B compartments. The authors convincingly attribute these discrepancies to cell population impurities in earlier work.

Refined measurements of loop size across stages, revealing a reduction in diplonema—a novel observation.

Early onset of meiotic chromatin reorganization, occurring before the traditionally defined meiotic entry point and marked by the transition from mitotic to meiotic cohesins.

Well-positioned chromatin loops detected by Micro-C during prophase I, in contrast to lower-resolution Hi-C studies that suggested loops are randomly distributed along chromosome axes.

Interaction between CTCF loop anchors and meiotic hotspots, peaking at the zygotene stage.

Fundamental aspects of gene regulation and interactions between regulatory elements and local sub-compartments are well preserved during all prophase I, despite extensive chromosomal reorganization and pronounced fluctuations in transcriptional activity.

Thanks to the high quality of the data, the work of Cheng et al allows resolving several important questions in the field. Moreover, beyond its immediate impact in the field of meiosis, this study offers important insights for a broader audience interested in chromosome biology and gene regulation. I therefore strongly recommend its publication in Nature Communications with some minor but important points to be addressed.

Main points:

1. The authors do not provide information on the bouquet formation and whether this conformation is observed in their data. It would be nice to address it or mention whether they were able to see it.

2. In the same line, it would be interesting to provide a separate analysis on what's happening on the X/Y and specifically in the PAR region, what about the loop size in the PAR? Is it indeed different from autosomes as it has been suggested? Are loop sizes the same/similar across chromosomes in general?

3. As the experiments were performed on B6 × CAST hybrids, the question of homolog interactions (ie trans contacts) arises naturally. As this is entire additional analysis, I do not think that it is mandatory to include in this paper and can maybe be included in a future work, but it is somehow odd for the reader that it hasn't been addressed/mentioned.

4. In relation to their observation of TAD attenuation prior to meiosis in spermatogonia, it would be informative to compare these data with those from somatic cells undergoing mitotic division. Since spermatogonial differentiation involves mitotically active cells, the observed TAD attenuation could be similar to that reported in mitotic contexts.

5. The decrease of the loop size from pachytene to diplotene is interesting and not seen in previous HiC papers. The authors might want to comment more on what might be happening (axis disassembly? Cohesin turnover?)

6. The authors provide solid evidence for a direct interaction between CTCF sites and meiotic hotspots. However, this finding is not entirely novel but confirms earlier, indirect observations: Grey et al. (Genome Research, 2018) and Biot et al. (Molecular Cell, 2024) showed that PRDM9 (ie hotspots) and CTCF can co-immunoprecipitate and that crosslink ChIP experiments for either factor reveal "shadow" peaks corresponding to the site of other, which led to speculation about a potential interaction of hotspots and CTCF sites. Moreover, in two former HiC papers, Vara et al and Zuo et al. the authors found CTCF and cohesin sites to coincide (ChIP). To my mind these former results nicely complement the current study which finally provides direct, high-resolution evidence for this interaction, strengthening our understanding of CTCF's involvement at hotspots. It would be important for the authors to acknowledge/discuss these previous observations and situate their results in this context.

7. In the same line, the authors state that they are the first to identify specific sites associated with the meiotic axis. And in line 373 they state "To date, there is no evidence for DNA sequence specificity in loop positioning in mammals". However,

this is not entirely true as this question has already been addressed in several earlier studies using different approaches. For instance, one study reported an enrichment of SINE elements, suggesting that they may contribute to axis formation (He et al. Dev Cell. 2023), while another described SYCP3 enrichment at CTCF sites, promoters, and DSB sites (Biot et al. 2024). These prior observations should be acknowledged and integrated into the discussion. Importantly, this does not diminish the impact of the present study, which provides direct evidence that specific sites at loop bases themselves constitute, at least in part, the foundation of the meiotic axis, whereas former data was in part more indirect.

8. Line 338 the authors state that "chromatin organization that results from balancing between the dense linear loop array configuration and maintaining active transcription". Using the data from Gaysinskaya, V. et al.2018, they correlate RNA-seq data with their aggregation plots (Fig. 6E). However, this statement (not necessarily the analysis) should be refined. Transcript abundance measured by RNA-seq does not directly reflect active transcriptional activity, specifically in the context of meiosis. Indeed, a recent dataset of Bellutti et al. (2025) demonstrated that transcription is silenced and transcripts are stabilized at the initiation of meiotic prophase I, transcription is then massively reactivated in pachytene. This was demonstrated by integrating scRNA-seq with EU-seq, which quantifies nascent transcripts to assess ongoing transcription. It is thus very interesting to note that, albeit transcription itself experiences a strong variation from "on" to "off" and "on" again (during the stages Cheng et al examined the contact maps), P-P and P-E interactions hardly change. The authors might consider highlighting this observation in their discussion and add the reference of Bellutti et al. DOI: 10.1093/nar/gkaf146

Additional, minor points

When discussing genome organization at different scales, it would be helpful if the authors provided approximate size ranges for each structural level. Including a chromosome schematic with highlighted regions under analysis in figures, such as some of the contact maps, is not mandatory would maybe improve clarity.

Line 51: The reference of Müller et al. (DOI: 10.15252/msb.20188293) is missing and ref 11 would better fit line 40.

Fig 3A: Dashed gray lines showing contact probabilities from samples without NIPBL. I think it is not obvious to all readers what is expected from a NIPBL null sample, this needs a short explanative sentence such as for example: When NIPBL is depleted, the ability of cohesin to extrude loops is decreased, leading to a genome-wide loss of cohesin-mediated loops and increased compartmentalization.

Fig 3C: I am not convinced that the interval should be called "cohesin density" as really it shows loop density wouldn't it be more accurate?

Fig S10E: lacks annotation on X axis

Line 206/7: Odd sentence please correct. (First, the median distance between the two loop anchors was nearly 3 times larger than in pachynema that in mES cells (620 kb vs 235 kb)).

Fig 4D: careful in the legend its written 100kb but on the figure it's a 150kb flanking window

Fig. S11B: The figure should be simplified for more clarity. There might be confusion: for example in the middle panel it says + + but there is also + - depicted.

Figure 4 legend mix up: Legend mix up: legend F seems to fit Figure 4E, then Fig F has legend G. legend E is for figure G

Figure 4G: (legend 4E?): There are distinct dots on the top line showing interactions upstream of the CTCF + site with the CTCF - site (above the green arrows), this seems to be specific to Z-D stages. Are these dots real/artefact? If real yes what are these sites? The authors might want to comment on these dots.

Fig S13A: there is a typo in the title: "de novo" and not "de nove" loop)

Line 328: typo (loo)

Line 286 punctuation missing

Version 1:

Reviewer comments:

Reviewer #1

(Remarks to the Author)
The authors have carefully addressed all concerns and the paper is suitable for publication without modification.

For the authors interest, two points.
1. From my understanding, the canonical start of meiosis is G1, not S-phase. For example, in budding yeast, the conditions

needed to switch to meiosis cause cells to arrest after the last mitotic division and before meiotic DNA replication, and nuclei get bigger during this period. This is a small point and does not detract from their findings. Just to keep in mind.
2. Meiotic chromosomes have closely conjoined sisters. Banded lateral element structures suggest that sister structures are in register, e.g. as "dual loop modules". Of course, HiC cannot tell sisters apart. Again, just to keep in mind.

This is a lovely paper and a major contribution.

Reviewer #2

(Remarks to the Author)
I would like to thank the authors for the thorough and thoughtful revisions made to the manuscript. I found the revised version a pleasure to read, and the additional discussion together with the new figures further enhance the clarity and overall quality of an already excellent piece of work. The points raised by this reviewer have been satisfactorily addressed. I also appreciated the perspective offered by the other reviewer, which was particularly insightful, and the resulting revisions have led to a well-balanced and carefully discussed manuscript. Overall, I believe the manuscript is now ready for publication and will significantly advance our understanding of chromosome organization during meiotic prophase I.
I have only a few minor remaining comments:
• The legend of Fig. 4g would benefit from a brief explanation of the pink and green arrows, which would facilitate interpretation of the figure.
• In Fig. 6e, there is a minor typographical error (“Midium” instead of “medium”).
• Line 448 (Discussion), don’t the authors mean switching from “condensin to cohesin” and not from “cohesin to condensin” referring to mitosis exit?
There remains, some unease with the use of the term “cohesin density” in Figures 3b, c, and f. While my initial proposal to call it “loop density” was indeed entirely inappropriate, the P(s)-based derivative interpretation integrates several parameters, including cohesin abundance (density), residence time, and extrusion activity. Referring to this quantity as “relative cohesin density/activity” or “relative cohesin density/stability” rather than “cohesin density” on Y axis would therefore help avoid overinterpretation. In addition, Fig. 3f reports relative protein abundance derived from Western blot quantification rather than relative density; relabeling the Y-axis accordingly to “relative quantity” would improve clarity. Moreover, although RAD21 and REC8 are plotted on the same scale, they are detected using different antibodies and should therefore be interpreted only in terms of their respective trends across stages, this might be mentioned in the legend.
These comments are intended as minor clarifications and should not delay publication; the editor’s judgment may guide whether these changes are necessary.

Open Access This Peer Review File is licensed under a Creative Commons Attribution 4.0 International License, which permits use, sharing, adaptation, distribution and reproduction in any medium or format, as long as you give appropriate credit to the original author(s) and the source, provide a link to the Creative Commons license, and indicate if changes were made.
In cases where reviewers are anonymous, credit should be given to 'Anonymous Referee' and the source.
The images or other third party material in this Peer Review File are included in the article’s Creative Commons license, unless indicated otherwise in a credit line to the material. If material is not included in the article’s Creative Commons license and your intended use is not permitted by statutory regulation or exceeds the permitted use, you will need to obtain

permission directly from the copyright holder.
To view a copy of this license, visit https://creativecommons.org/licenses/by/4.0/

RESPONSE TO REVIEWERS' COMMENTS

General response:

We would like to sincerely thank the reviewers for their thorough reading of our manuscript and for their insightful, helpful and constructive comments. We're glad that they found our experiments well executed and our results exciting. Below, we synopsize all changes and answer the specific points raised by each reviewer.

Reviewer 1:

This is a very important and interesting paper. It applies the latest chromosome capture methodologies to highly synchronous populations of mouse spermatocytes to provide unprecedented spatio-temporal resolution of "chromosome folding" during meiosis. It presents a large number of interesting and new findings which I was very happy to read about.
I have made quite a few comments below. I would like to emphasize that this is not a reflection on the quality of the work, which is outstanding, or the importance of the conclusions, which are substantial. Rather, it is because the data and conclusions are complex and the presentation and integration of those findings is sometimes challenging. I would like to see things even cleaner in presentation than they are at present. Also, if this were my work, I would place more emphasis on certain things (with which the authors may not agree, or maybe they can take this into account). Basically, I'm hoping that these comments will help to make the revised version of the paper better. I hope the authors are not too unhappy with these inputs.

Response:
We again thank the reviewer for the careful reading of our manuscript and thoroughly appreciate the form and substance of the comments.

For this reviewer, the following key points are important:
1. AB domains disappear during meiosis. This is clear in the current findings; makes obvious sense given that meiotic chromosomes are organized in cooriented sister linear loop arrays. It corrects findings to the contrary in the literature which the authors demonstrate can be explained by impurity of samples in previous work. (The presence of the sister and the homolog needs to be remembered; point 4 below).

2. TADs disappear during meiosis. This has been shown previously and it is apparent in the current work and shown very cleanly.

3. A key issue in the meiosis field is loop sizes. The P(s) curves suggest that loop sizes increase with time up to/through pachytene. The authors have avoided showing later stages, perhaps because there is an apparent discrepancy between cytology and HiC conclusions. But this is not the correct approach; these results should be included in the text. The results presented in this paper provide pretty good evidence that the situation is that loop sizes go from 100kb to 200kb to 600kb. This comes not only from P(s) curves (interpretation of which depends on modeling, as the authors point out, but from the independent analysis of distances between loop base contacts as presented in this work, which is a very important addition to the situation). Cytology estimated

100-200kb loops. But the result here is validated by the fact that when the appropriate loop size is used, the density of loops now correctly matches the "evolutionarily conserved" density seen across many organisms. As pointed out by deMassy (Frontiers review), cytological estimates have to assume a particular packing ratio of the chromatin fiber, which is not known. If the assumed ratio was too low, the loop size will be too short, as is apparently the case. The findings in this paper allow a satisfying resolution to this issue, at least as a sensible replacement for current confusion. It would be useful to know how this compares with loop sizes along mitotic prophase and prometaphase chromosomes (see discussion of stages below). Should be the same as mitotic prophase and less than prometaphase.

<u>Response:</u>
Thanks for this comment, we agree that some clarity on loop sizes is an important addition to the field. In the submitted form of the manuscript, the loop sizes for later stages derived from Hi-C data were shown only in Supplementary Figure 5B and not in the main text because the trend generally follows that from previous published studies. We appreciate the review bringing this up and agree that the terminology “simulated loop size” (point 19) helps the reader.

Specific changes:
Lines 154-171: Emphasized the simulated loop size throughout all stages
Lines 448-457: Added discussion on why simulated diplonema loop size might be different to that reported previously.

4. Meiotic chromosomes are known from cytological studies to comprise loops emanating from a complex structural axis meshwork. Previous work failed to detect signals that might represent loop base contacts. The present work detects such signals as "dots" which do not occur at the tips of TAD triangle signals. This is very important, in addition to allowing an independent assessment of loop sizes (above), for two reasons.
- The data allow the authors to suggest that loops do not form by loop extrusion along meiotic chromosomes. Their reasoning and intuition seem correct. Several points about this conclusion could be mentioned. The authors discuss their findings by saying that loops are "dynamic" in mitotic chromosomes (interphase) and "stable" in meiotic prophase. "Stability" is not measured directly but is inferred from the strength of the signal; this could be emphasized more.

The issue of meiotic vs mitotic prophase is also important in this context, as discussed below; this may not be meiosis-specific.
It is clear that there is an important issue: interphase mitotic loops are dynamic/transient whereas the prophase structure is morphologically stable. The same stability may well be true at mitotic prophase. However, it is also important that adjacent loops are sort of stochastic in nature in interphase but have a very specific spacing along meiotic prophase chromosomes. Packing of adjacent loops side-by-side is a different point from stability. In principle, a morphologically constant array could arise with subunits coming and going. In fact, such a situation is known for the meiotic SC (C.elegans work). And it is known (although I am not sure it is published) that meiotic worm chromosome axes do NOT show this same behavior. So it is clear that there is also a stability difference, but not directly from the current work.

Regardless, the observed patterns do strongly argue against loop extrusion as a mechanism and instead match a recent model from mitotic chromosomes (which the authors discuss) in which loop arrays form by progressive extension. Their discussion of this point is spot on correct. The differences in CTCF patterns are interesting and important and novel. The authors may also wish to appreciate a model (1999) in which meiotic prophase loops occur by collisions, with loop size determined by chromatin fiber persistence length; this could still be possible. Whether it is progressive along chromosomes or not is another issue....Outcome is a series of adjacent modules, but whether they are progressive in time is a different point. Interestingly, unpublished data from Franz Klein could argue against extrusion for meiotic prophase in yeast (they could ask him about this).
To illustrate the above issues, in the Discussion, line 403: "Our data cannot formally distinguish between active loop extrusion or multiple loops forming statically and then fusing. We favor the hypothesis of loops forming statically rather than dynamically due to the presence of corner dots without TADs." NB conflation of loop formation mechanism vs loop "stability"...needs to be clarified in language.

Response:
We generally agree with the reviewer and have worded this far more carefully in the revised manuscript. We have tried to be more explicit on what we meant by stability (of the loop array) versus the dynamics of loop formation throughout the manuscript.

Specific changes:
Lines 427-447: Expanded discussion clarifying loop formation mechanisms.

- The detection of loop base contacts leads, along with other data, to further analysis of CTCF/Rec8. That analysis is very useful and important information. The authors are more excited about the molecules than this reviewer, who thinks that the really important point is the implication for how loops form.
Thus, as a secondary point: in presenting these findings, the authors tend conflate detection of loop base contacts with analyses of CTCF/Rec8. These two issues should be more separated. Detection of these contacts is important regardless of what molecules are involved. In this regard, also, the authors make the factually correct statement that loop base contacts were observed in yeast but not in mammals. This is true, and the fact that these contacts are detected in the present has yielded a lot of new information about molecules and about the nature of loop formation, all of which is seminal and important. But it can be noted that the explanation for the difference in the findings in the two organisms has a deeper reason. In yeast, the number of loops is very close to the number of potential axis association sites. So in a total population, all combinations of loop possibilities (and thus dots) are visible. In a mamalian cell (with longer loops, e.g.) there are many more loop possibilities and thus dots will be fainter. If I understand correctly, tyhis is why the current analysis is, for the first time, technically able to detect these contacts. The authors focus on Rec8 or not Rec8, but this isn't really the issue maybe...... I think that is not the reason for the

failure to detect contacts in mammals previously. The current study has the resolving power required.

Response:

We are grateful for the reviewer's thoughtful comments. We agree with the reviewer on the differences in loop detection between yeast and mammalian genomes. Following the reviewer's suggestion, we have restructured the section to present the detection of distinct "dots" first, before discussing the underlying molecule.

Specific changes:

Line 188: Section **CTCF shapes meiotic chromatin organization** rearrangement

5. On established chromosomes, CTCF is the important player, versus Rec8. Aside additional implications, this is an important general finding. The difference with yeast is important to note although, of course, yeast has to make do with fewer molecules and doesn't have CTCF, so the same principles likely apply nonetheless.

Also: is it possible that loops are formed by Rec8 seeing e.g. locally AT-rich regions with CTCF then seeing the same positions concomitantly? This might be an alternative explanation for the absence of convergent CTCF patterns? (although I like the authors' explanation). See DOI: 10.1093/nar/gkaf587.

Another issue that might be addressed: is it possible that the HiC axis dots are in some way an artifact? In a closely packed situation, a certain genomic region can contact any region nearby, which may mask specific patterns like TADs. Additionally, fixation and ligation may be highly biased towards a dense protein/DNA network along chromosome axes, which potentially causes "artificial" contacts. This does not seem likely, given the resulting loop size implication, and especially as meiotic chromosomes are not SO compact (unlike mitotic prometaphase chromosomes) - rather they are just well-organized. Perhaps this could be discussed a bit.

Response:

Thank the reviewer for the comments. It is formally possible that Rec8 is bound to AT-rich regions that happen to have CTCF. The conclusion of reduction in orientation bias is from an analysis performed on dot anchors overlapping with CTCF peaks with one typical motif, without considering the flanking sequence features. We have now examined the sequences around these sites compared to CTCF motifs that are not involved in the detected loops and failed to see any nucleotide composition deviation from the genome average. This, and the extensive body of literature showing that CTCF - in any orientation - can position cohesins, made us favor the the hypothesis that a closely positioned array of loops is the primary reason of the high interaction frequency between non-convergent CTCF binding sites.

Thanks for bringing up the technical issues that could result in artifactual dots. We think it is an unlikely scenario:

1. Only 21% detected dots have both anchors lacking CTCF binding. The anchors would be more randomly distributed if the dots were detected as a result of a highly packed genome or technical bias in fixation/ligation.

2. The loop size calculated based on detected dots increases accordingly from meiotic-S stage to pachynema. Such a trend would not be expected from artifact loops.
3. In each stage, the loop sizes follow normal distribution around the median size. If the dots are from an artifact, loop sizes would follow a random distribution.
4. More importantly, as discussed in the manuscript, the median loop size can be translated to ~ 20 loops per micron.

6. The authors emphasize the nature of the transition into meiosis. These findings are an important addition to understanding. They find that detectable changes occur very early and they argue that an "unstructured" intermediate (Transition I) is important. This is all well-documented. However, there are several points that could be discussed/clarified.
- Is it known, when cells enter Transition I, whether they do so from a particular stage of the preceding/ongoing mitotic cell cycle? The authors seem to think they are entering from interphase, but maybe they are entering from telophase of the prior cycle? or? And are there divisions going on during this transition or ??

Response:
We did not rigorously determine the cell cycle at which cells entered Transition I. However, if we gate on replicating cells (2C–4C) or tetraploid cells (4C) based on the DAPI signal during sorting, the entire population is lost. Although DAPI intensity alone may not perfectly distinguish ploidies, dividing cells in the transition I population are expected to present only a very small fraction. We believe that cells enter the Transition I stage after completing their final mitotic cycle.

- The authors argue for a two-stage progression. Dekker has published that there is a two-stage progression in the telophase/G1 transition of mitotic cells. It would be useful to compare the two cases. They could be functionally analogous in the sense that a first stage allow entropy-driven decatenation and the second allows reorganization without recatenation.

Response:
We thank the reviewer again for the helpful comments. We recognized the similarity between Job Dekker's condensin-to-cohesin transition during mitotic exit and our two-stage mitotic-to-meiotic cohesin transition. Indeed, as mentioned in the paper, the detection of changes in cohesin was inspired by their study.

7. DSB/recombination sites colocalize with the loop base contacts. This is useful and important, and is strong validation of the identification of axis association sites, but is not new/surprising, given long history of "tethered loop axis complexes". Right? Also

Response:
We agree with the reviewer. Reviewer 2 also pointed out that earlier studies had already proposed an interaction between hotspots and CTCF-bound loci, supported by co-immunoprecipitation of CTCF and PRDM9 and ChIP-seq data for either protein. This result is not surprising, but it provides solid evidence supporting these contacts.

Specific changes:
Line 285-288: Added context and references

a. The authors make the statement that this is progressively lost with time as repair proceeds (line???). This is not very precise. Crossover interactions remain associated until diplotene. Noncrossover interactions are released from the axis at early pachytene (Ogawa and general knowledge). Result is reduced localization of recombination complexes to axes. This point could be usefully made to explain their result, since it provides evidence for such release.

Response:
We appreciate the reviewer's suggestion on this point. The interaction frequency indeed depends on the average strength of the hotspot association with the axis. The release of recombination complexes more directly explains the reduction in signal from zygonema to pachynema and then to diplonema.

In addition, we added a panel to the figure that addresses this question directly. We first identified the most commonly used DSB hotspots in the B6xCAST strain, then assigned a CO resolution probability based on COs identified by Yin *et al*. Of the strongest 1,000 DSB hotspots, we selected the 250 ones more frequently resulting in a CO and the ones that didn't have a CO in Yin's data. This serves as a population proxy for DSBs that are likely to become a CO or not. When assessing its association with the axis (HS-CTCF interactions) the results match the reviewer's prediction. By zygotene the association of both sets is comparable, but by pachytene and diplotene, only the ones more frequently resolved as CO remain at the axis. We appreciate the suggestion and have modified the figure and text to reflect this important distinction.

Specific changes:
Figure 5, modified panel c
Lines 288-298: results now include support for the notion that CO interactions remain associated with the axis until diplonema.

b. The conclusion (Abstract) that " loops at defined sequences might be an important feature to keep homologous chromosomes in register" seems to me problematic. It is unclear that exactly the same loop bases are used along every chromosome as required by this statement??? This should be addressed. Maybe the authors think "yes" because their loop size calculations, based on this assumption, give the right answer? Also, the authors need to consider the fact that even sister chromatids are not aligned in register (Gerlich for mitosis, worm data for meiosis). Different loop bases are chosen in different sisters - the issue is a structural match of loop base modules, seen cytologically as banded lateral elements by EM - not a specific match at the DNA level. The authors have a lot of very interesting findings (above) but seem here to be reaching for sexier conclusions when that is not necessary. Implications for other things is more than sufficient to support the importance of this study.

Response:

This is an excellent point raised by the reviewer. We do not have any evidence for homologs to be in register. In response to the reviewer's comment, we have used a published method (**doi:** https://doi.org/10.1101/2025.01.13.632736) to estimate the interaction probabilities of the strongest loop (dots) detected in pachynema, with a caveat that the parameters were derived from mES cells. The loop occurs with a probability of 1.7%. If the loops were to form simultaneously on both homologs, the probability would be 0.028%, which is highly unlikely. We have removed this sentence from the abstract.

8. Interactions relating to gene expression are largely conserved. This is sensible given that gene expression must go on, and important to have shown. However, the authors have framed this as a "mitosis vs meiosis" issue on the presumption that meiosis is unique. But this is probably not true. As described below, meiotic prophase is analogous to mitotic prophase, not mitotic prometaphase (which is what the authors consider "mitosis"; further comments below). It would be better to discuss this in a broader way. Clearly it is important for meiotic prophase chromosomes to continue transcription. Given that loops are 600kb, with chromatin undergoing dynamic changes in diffuseness/compaction (below), it is perfectly reasonable that transcriptional links are still present. But there needs to be a discussion of the length scales of the examined contacts, relative to loop size (and maybe even relative to loop base positions?). Again, this is very important information - it just needs to be discussed a bit differently, according to me, at least. The authors emphasize: (Line 338) "Our data revealed a unique global chromatin organization that results from balancing between the dense linear loop array configuration and maintaining active transcription.". This seems to me to be a bit of a straw man. Why should an array of 600kb loops preclude transcription? No need to over-hype this point, especially since contrast they make is with mitotic prometaphase, which is an entirely different issue.

Response:
The point is well taken, the extended meiotic prophase I is indeed distinct from mitotic prometaphase. We have clarified these comparisons throughout and pointed out the fleeting nature of mitotic prophase and discuss the data available.

In supplementary Figure 20, our data showed that the interaction between regulatory elements were detectable at a distance up to ~25 Mb but diminish at greater distances during meiosis I prophase. We did not see an association between this distance with loop size. It is more related to local compartmentalization.

Specific changes:
Lines 359-363: We highlighted the finding that transcription can be maintained even in the absence of TADs.

General comments. There are some general issues that could be better addressed.

9. It might be useful to start with a better description of what is known about meiotic chromosome organization, rather than an extensive discussion of mitotic chromosomes which, for this reviewer,

is not so interesting and which does not necessarily have the relevance that the authors think (see point 2 below)

Response:

We appreciate the reviewer's suggestion. We have clarified the comparison regarding mitotic chromatin throughout the manuscript and restructured the discussion part.

10. The term "compaction" is not accurate and should be avoided. This is a general problem in the mitotic and meiotic fields. One difference between "interphase" and "later stages" is that the chromosomes are organized and individualized ("structured" in these authors' terms). A second issue is whether the state of the chromatin fiber is altered. In mitotic prometaphase chromosomes, there is also chromatin compaction as well as organization (e.g. Gerlich). In meiosis, it has been shown, albeit without extensive documentation, that chromatin alternates between more and less diffuse states (so-called "expansion/compaction cycles" (Kleckner, 2004, 2006; Zickler and Kleckner 2023). This is important generally. It is also important because some of the stage-specific effects that the authors report here could reflect these changes: expansion at leptotene, compaction and zygotene, expansion (maybe with complexities) at pachytene, and compaction at diplotene. This might be particularly true for microC results.

Response:

We are grateful to the reviewer for pointing out the potential ambiguity when using 'compaction' in the context of meiosis. Accordingly, we have removed this term throughout the manuscript.

11. The authors compare "meiosis" (meaning meiotic prophase" to "mitotic chromosomes" (meaning mitotic prometaphase), with differences attributed to special features of meiosis. However, this is not a correct view of the mitotic situation. Mitotic chromosomes go through a prophase stage which is, as far as can be told, the same as meiotic prophase. This is shown by Liang et al., 2015. It is exemplified by the fact that, in muntjac, the lengths of meiotic SCs are identical to the lengths of chromosomes trapped in prophase by TopoII inhibition. In the authors' defense: the HiC-related community fails to understand/accept this relationship because it is a transient stage and requires single cell imaging to see; it is missed or otherwise avoided in standard synchronized HiC experiments. This is important, because the HiC community also recognizes that they don't understand mitotic prophase (although perhaps recent studies of which I am unaware address this point) and because it may well be that the features seen here for meiotic phase are not meiosis-specific but rather are "prophase-specific". Both possibilities need to be discussed.

Response:

As mentioned above, we have clarified the comparison of mitotic chromatin throughout the manuscript. The reviewer raises an excellent point: Mitotic mid-prophase chromosomes share strong morphological similarity with meiotic prophase chromosomes. The same chromatin length strongly indicates a shared loop size. Interestingly, a recent publication shows considerable variation in mitotic loop sizes across species, suggesting that prophase loop sizes may vary

accordingly. However, to the best of our knowledge, Hi-C data for mitotic prophase in mouse cells are not yet available. This is discussed in our revised discussion section.

Specific changes:
Lines 475-481: Added discussion to highlight mitotic vs meiotic prophase similarities.

12. More generally, this reviewer would prefer to see the authors simply describe everything they see and THEN go back to (a) explaining previous meiotic results and why they were right/wrong; and (b) providing comparisons to mitotic prometaphase chromosomes with the above in mind. These results stand on their own and need to be presented and interpreted and understood on their own. The rest can follow.

Response:
We appreciate this suggestion. We assume that this suggestion is specifically for the section of "**CTCF shapes meiotic chromatin organization**". In this section, we presented our conclusion before showing the data. We structured it this way with the expectation that it would help readers follow the data more easily. Following the reviewer's suggestion, we have now moved the conclusion to the end of this section.

13. The authors assume that all of the special features that they detect are due to organization along individual chromosomes. But there are several caveats. The most important is that this is meiosis and so there are homologs and it is not excluded that there are interactions between homologs which feed into some of the findings. This is not certain, but as the authors know, HiC can't tell the difference. MicroC might be too short range for this to matter. Homologs may interact specifically by pairing, and they might interact without directly pairing due to architectural features that tend to coalign them anyway (centromere/telomere pairing?) at early stages particularly. By pachytene, homolog chromatin probably lies on opposite sides of the SC mostly; at coalignment, this may mostly be true but some contacts are possible given that chromatin is not Some comment about this would be helpful. Additionally, of course, there are interactions between sister chromatids. (And then there are chromatin expansion/compaction variations; above).

Response:
The reviewer raised an excellent point regarding Hi-C studies of meiosis where homologs are in close proximity and can interfere with our "individual chromosomes" analyses. We performed all experiments presented here in B6xCAST F1 hybrids, and to reduce such interference, detectable inter-homolog interactions were filtered out before performing any analyses (The CAST and B6 genomes differ by roughly 20 million genetic variants). We further quantified the proportion of inter-homolog interactions within all allele-assigned contacts. The ratio peaks at pachynema, at about 0.6%. Therefore, we assume the interference from inter-homolog contacts is minimal. Conversely, the low detectable rate also precluded us from drawing any confident conclusion for inter-homolog interactions.
The inter-sister contacts are expected to have a higher ratio than inter-homolog contact given they are closely aligned. As estimated from somatic G2 phase, the ratio can be up to 23.7% (DOI:

10.1038/s41586-020-2744-4). However, the inter-sister contacts do not largely interfere with the Hi-C profiles of G2 cells, which closely resemble those of interphase cells (DOI: 10.1126/science.aao6135). This is likely because that cis-contacts remain dominant. We agree with the reviewer that the contribution of inter-sister contacts to our observations should be evaluated in the future.

14. Different chromosomes are shown for different points. Explain why and which things are/are not chromosome specific? Maybe this is in the SI?

Response:
The observations presented in the figures are consistent across autosomes. The Y chromosome shows little signal in the contact map due to its highly repetitive sequence. The X chromosome retains autosome-like interaction features up to pachynema, which subsequently diminish as a result of meiotic sex chromosome inactivation (MSCI). The following outlines the rationale for choosing representative examples for each figure.

In Figure 1, the changes in global structure and local compartment are not specific to any autosome. Chromosome 1 was chosen randomly.

In Figure 2, the changes in TADs can also apply to any autosome. The representative was chosen from the regions exhibiting relatively strong TADs.

In Figure 4, the dots can be observed in other autosomes (a few dots are visible on Chr X). The representative image was chosen from the regions exhibiting relatively strong dots. In Figure S9, dots from three other regions were shown as extra examples.

In Figure 6, the interaction patterns at a resolution of 400 bp can also be observed in other autosomes. This region was chosen because the same region was shown in the published mES cell paper. This region included the right number of genes, which is easier to annotate and present.

Abstract. Overall, the authors have made many important and interesting findings. It is difficult to corral them around one or a few major points.....They can improve in this respect. As a general point, because there is so much important information, it is difficult to write an abstract that gives the major message. But more effort should be made in this regard, with less of a shopping list of outputs.

Also, the points emphasized seem to come from a "mitotic" perspective which, for this reviewer, is less interesting. The findings are really important for meiosis qua meiosis.
Generally speaking, as stated elsewhere in this review - given 600kb loops that vary in diffuseness, it is hardly surprising that very local interactions are maintained and transcription can continue. Probably active genes are in loops? This reviewer is less interested in such issues than

in what these findings say for meiosis, which is really important and is the main subject. That being said, the ES cell/meiosis comparison is useful.

<u>Response:</u>
We have reworked the abstract to incorporate the reviewer's suggestions.

14. See point re "condense" above.

15. "local subcompartments...are maintained". This should be stated another way. Local subcompartments are DETECTED, not "maintained", unless they are referring to very local interactions. This is unclear to people thinking of loop/axis arrays. More generally, the Calder analysis in Figure 1 seems disconnected from subsequent findings and is sort of a HiC-specific issue....Perhaps I am not understanding the significance (Discussion lines 329ff?). That section also says: local compartments are more visible in meiosis due to changes of cohesin activities. Well, really, the point is that local compartments are more visible because the linear loop array is more regular and the noise from random TADs and AB domains is gone. What molecules are involved is another issue.

<u>Response:</u>
The reason for Calder analysis is further explained in the response to point 17. By using "maintained", we intend to emphasize that the compartment intervals detected in prophase I mirror those observed at earlier stages, indicating that they are preserved rather than newly formed.
We agree with the reviewer's comments regarding the underlying reason for the increased visibility of local compartments during meiosis.

Specific changes:
Lines 354-358: Clarification on the local compartments

16. "challenging the current view that loops are positioned randomly along axial elements". See above: failure to detect specific loop bases is not the same as "positioned randomly" which is clearly silly. This seems an overstatement. Put the result positively - for the first time can detect loop base linkages and this leads to reconciliation of loop size issues and interesting new suggestions for how loops form along meiotic prophase chromosomes.

<u>Response:</u>
We take the reviewer's point, and the abstract has been revised.

Results. Perhaps I have not fully understood, but these are points to consider clarifying?

17. Figure 1. There could be more discussion of the details of the analyses.
a. One general point is that PCA and Calder reveal the presence of organizational features, but do not do a very good job of capturing the overall situation as defined by the intensities of the primary signals, which seem to tell an additional story, if I understand correctly. Irrespective of

PCS and Calder, it is obvious that defined patternrs decrease at S-leptotene transition. Notably, it is known that meiotic chromosome organization develops after S and before leptotene, so during this transition, so this data fits with what is known. This should be pointed out. Some data are in recent yeast paper by Nozaki et al in Nature. Plus it is obvious that there is not just a general smear of contacts after that point, so something remains. What those remaining contacts are is studied further.

b. What is the point of the Calder analysis? Does it have a specific organizational meaning? Is it a reflection of features described later? This should be clarified. I didn't find it helpful, although of course it has to be included. Or maybe I am missing something important

Response:
In PCA, eigenvectors are calculated based on the correlation between all pairwise bins. In later stages of prophase I of meiosis, the contacts beyond around 30 Mb become extremely rare, which make the eigenvectors less useful. Although the local "checkerboard"-like patterns on the interaction maps of stages after leptonema were clearly visible, PCA failed to detect these structures. Unlike PCA, Calder uses short-range intra-chromosomal interactions to classify the compartment intervals and works very well on detecting the domains that are visible but cannot be detected by PCA. Calder analysis allowed us to examine the similarity of identified compartment intervals throughout all the examined stages, validating the observed local "checkerboard"-like patterns are indeed compartments. As discussed in the paper, various terms have been used to describe this observation, and before our work, this structure was thought to be unique to pachynema

c. There are complexities within PCA signals that seem interesting and should be discussed if they are meaningful. Obviously AB compartments start to go away at S/Leptotene transition, which is sensible (above). NB that events along chromosomes occur asynchronously through the nucleus, so "leptotene" includes some regions that have done coalignment and some that have not as well as some that maybe haven't developed structure. More importantly, at zygotene, there is some SC, but alot of regions haven't even done coalignment. In any case, linear loop arrays are developed by leptotene. So why are there changes in AB scores after that point? Are they meaningful? Is there something special about middles vs ends of chromosomes? Do any of these effects reflect centromere/telomere/end clustering? Changes in chromatin compaction? At diplotene, are there B components only at the centomere end of the chromosome? At later stages, in microC, the general background gets much fuzzier. Why is that? This analysis may be especially sensitive to expansion/compaction of chromatin? Why are there Calder contacts only in B domains (green)? What is going on with PCA data from zygotene to diplotene? Are these changes meaningful?

Response:
As stated before, we don't believe the PCA analysis is providing a meaningful description of the data. It is useful to show that compartments disappear upon entering meiosis, but the values are close to zero and become noisy. The eigenvalues are assigned, but it is not reproducible. It tends to identify 2 or 3 big compartments per chromosome. These "compartments" don't seem to

correlate with any underlying structure and are not always the same when using subsets of the same matrices. Calder contacts are detected both in A and B domains. In panel c) we show a zoomed-in matrix, that in this example, at diplonema, spans the green compartment only, that is why it looks like Calder contacts are only in the B domain.

d. Panel D is not understandable to the non-expert and Panel is not useful, particularly because Calder analysis is ambiguous. There could/should be a proper summary cartoon of what happens at various stages in the Discusion.

Response:
In Panel D, we compared the similarity of the identified compartment interval among germ cells with two B-cells as controls, showing that the compartment intervals detected in prophase I of meiosis are more similar among germ cells than to those of unrelated cell types.

Specific changes:
A cartoon has been added as Figure 7

18. Figure 2.
a. Panel B - it is hard to see the patterns under the grey lines. Remove them from most panels or make light/dotted.

Response:
The grey lines have been removed.

b. The insulation score and its significance is never discussed. For the non-expert it would be useful. Is it basically the prominence of the TAD? or its width? or ??

Response:
The insulation score is a quantitative measure of insulation between genomic regions flanking each bin. Local minima in the insulation profile are identified as TAD boundaries. The difference between local maxima and minima can be used to quantify the prominence of insulation between TADs.

Specific changes:
Lines 143-145: Significance of insulation is now mentioned.

19. Figure 3.
a. The data for later stages should be in the text and properly discussed.
b. "Loop size" (as the authors point out) is a HiC computation feature. It should be called "simulated loop size". It can be mentioned that results are/are not confirmed by analysis of loop bases below.
c. Save Panel G for the Discussion.

Response:

We agree with the suggestion of “simulated loop size” and the loop sizes are in the text in the revised manuscript. Panel G has been removed, and a new figure summarizing results across all examined stages has been added.

20. Figure 4.
a. Panel a was confusing. Legend does not explain mES cell portion. it should be more obvious. And what are we to conclude about positions of black arrow dots in mES cells? Are they there or not there?

Response:
The “dots” observed in mES cell are annotated in the new figure.

Specific changes:
Figure 4 legend - explanation of the different dots in the panels

21. Figure 5.
"stablizing loops". What is measured is the steady state level of loops as given by intensities of signals. This should be made clear. Stability is not directly measured but inferred from higher steady state levels.

Response:
We have changed the wording to: “CTCF plays a role in anchoring cohesin complexes and meiotic loops”

22. Figure 6. Move panel F to Discussion? Overall, Discussion needs to give a synthetic picture of what happens in meiosis at various levels, from their data.....

Response:
Panel F has been removed, and a new figure summarizing results across all examined stages has been added.

Discussion. Most of my comments are given above. One small point - line 353. "Interestingly, we observe higher insulation between genes at pachynema and diplonema. This phenomenon could potentially be the result of a significant accumulation of “sticky” RNA Pol II on genes at these stages and the increased transcriptional activity." Huh? How about changes in compaction status? In any case, this is not really a big point compared to other finding. Maybe omit?

Response:
We agree with this assertion and the sentence has been removed.

I realized that there is a further issue regarding dots not at corners of TADs. The authors estimation of loop size assumes that same positions are used in all nuclei. The estimate matches their HiC P(s) curves and gives appropriate inter-loop spacing (20/micron axis). But using exactly the same positions in all cases is not, a priori, so likely (see comments re yeast). It would be worth

discussing what must be said for this to be the case. Perhaps the authors did this, and it is part of their idea that loops are phased on homologs. But do they understand the basis for it? Maybe I missed this point, but thought I would bring it up in case further discussion would be useful.

Response:
Thanks for the comment, when we detect a dot that means that oftentimes those two loci are in close proximity. It is indeed very unlikely that they are used in all nuclei. As mentioned before, we tried to quantify the likelihood of a given linkage to be present, and it is extremely low (~1.7%). We have included this in the discussion.

Reviewer #2 (Remarks to the Author):

Cheng et al. performed high-resolution chromatin mapping across stages preceding and during mouse meiotic prophase I in males, using both Hi-C and Micro-C. While their study follows a wave of publications applying Hi-C to examine spermatogonia and meiotic prophase, it stands out for the exceptional quality of their data and fine temporal resolution. This level of detail allowed the authors to uncover several findings that diverge from, or challenge, previously reported views of chromatin organization during mammalian meiosis greatly clarifying and correcting prior models. Their key findings include:

Loss of large-scale A/B compartments during prophase I, contrasting with most prior studies (with the exception of Vara et al.) that reported the persistence of typical A/B compartments. The authors convincingly attribute these discrepancies to cell population impurities in earlier work.

Refined measurements of loop size across stages, revealing a reduction in diplonema—a novel observation.

Early onset of meiotic chromatin reorganization, occurring before the traditionally defined meiotic entry point and marked by the transition from mitotic to meiotic cohesins.

Well-positioned chromatin loops detected by Micro-C during prophase I, in contrast to lower-resolution Hi-C studies that suggested loops are randomly distributed along chromosome axes.

Interaction between CTCF loop anchors and meiotic hotspots, peaking at the zygotene stage.

Fundamental aspects of gene regulation and interactions between regulatory elements and local sub-compartments are well preserved during all prophase I, despite extensive chromosomal reorganization and pronounced fluctuations in transcriptional activity.

Thanks to the high quality of the data, the work of Cheng et al allows resolving several important questions in the field. Moreover, beyond its immediate impact in the field of meiosis, this study offers important insights for a broader audience interested in chromosome biology and gene

regulation. I therefore strongly recommend its publication in Nature Communications with some minor but important points to be addressed.

Response:
Again, we are grateful for such insightful and careful reading of our manuscript.

Main points:

1. The authors do not provide information on the bouquet formation and whether this conformation is observed in their data. It would be nice to address it or mention whether they were able to see it.

Response:
We thank the reviewer for bringing this to our attention. Consistent with previous studies, we also observed an “X”-shaped pattern in inter-chromosomal contact maps, which was interpreted as the ‘bouquet’ configuration. However, we are somewhat concerned about this interpretation. A key reason is that “bouquet” is known as a transient clustering of chromosome ends at zygonema that disappears at early pachytene, whereas the “X”-shaped contact pattern remains detectable even at diplonema. We believe that the “X”-shaped inter-chromosomal contact pattern reflects the contact between two individualized chromosomes with their telomeres attached to the nuclear envelope. A more appropriate approach is to compare the trans-contacts between chromosome ends across stages. We quantified the trans-interactions between chromosomal ends (defined as regions 10 Mb or 20 Mb from either end) and normalized the values by cis-interactions from the same regions to mitigate stage-specific biases. The most frequent interactions at chromosome ends were detected in leptonema, with a dramatic reduction observed in zygonema, pachynema, and diplonema. Considering our leptonema is sorted as stra8 positive cells from the 4C population and a large proportion of our sorted zygotene cells are relatively late, Hi-C data with higher temporal resolution might be helpful to better understand the changes in interactions between chromosomal ends.

Specific changes:
Lines 113-122: New section in the manuscript to address this question.
New Supplementary Figure 3

2. In the same line, it would be interesting to provide a separate analysis on what’s happening on the X/Y and specifically in the PAR region, what about the loop size in the PAR? Is it indeed different from autosomes as it has been suggested? Are loop sizes the same/similar across chromosomes in general?

Response:
We appreciate the reviewer’s question regarding chromosome X and Y, as this was not discussed in the manuscript. First, the Y chromosome is mostly blank on the contact map, reflecting the limited resolution of standard alignment approaches in handling its highly repetitive sequences.

Second, the X chromosome retains interaction features comparable to autosomes up to pachynema. As reported previously, these features largely disappear thereafter due to MSCI (Meiotic Sex Chromosome Inactivation). We did not present these data because this phenomenon is already well documented. Third, alignment to the PAR region remains challenging, even with the current T2T reference assembly. While the latest T2T reference assembly may enhance accuracy somewhat, it cannot overcome this limitation. Nonetheless, we agree with the reviewer that examining the PAR loop size at different stages would be of great interest. This challenge needs to be addressed in future studies. Fourth, loop sizes vary across chromosomes, with greater variation in meiotic prophase I than in preceding stages.

3. As the experiments were performed on B6 × CAST hybrids, the question of homolog interactions (ie trans contacts) arises naturally. As this is entire additional analysis, I do not think that it is mandatory to include in this paper and can maybe be included in a future work, but it is somehow odd for the reader that it hasn't been addressed/mentioned.

<u>Response:</u>
We are grateful to the reviewer for noting this point, and we are pleased to clarify this matter. The reviewer is correct. When we started this project, we intentionally used hybrid mice because of the potential to detect inter-homolog interactions. However, we found that the proportion of inter-homolog interactions is very low, ranging from 0.3% to 0.6%. With our current sequencing depth, the number of valid inter-homolog contacts is too limited to draw any meaningful or confident conclusions.

Specific changes:
Lines 469-474: We have added a paragraph in the discussion to address this omission.

4. In relation to their observation of TAD attenuation prior to meiosis in spermatogonia, it would be informative to compare these data with those from somatic cells undergoing mitotic division. Since spermatogonial differentiation involves mitotically active cells, the observed TAD attenuation could be similar to that reported in mitotic contexts.

<u>Response:</u>
We agree with the reviewer that the spermatogonial differentiation involves active mitotic cell division, and that a heterogeneous population containing both interphase cells and numerous mitotic cells could account for the observed attenuation of TAD structures. However, in our current study, all the stages prior to meiotic-S were isolated from DAPI-defined 2C populations, a strategy that effectively removes the majority of mitotic cells. So, this observation is unlikely to result from contamination with mitotic cells.

While our manuscript was being reviewed, Mitinori Saitou's group (Masahiro Nagano et al., NSCB, 2025) showed that mitotic STAG3-cohesin complex attenuate insulation in spermatogonia because of its short residency time on chromatin slower loop extrusion. Whether STAG3-cohesin plays a role in our observation needs to be verified in the future. Meanwhile, the changes in the regulator of the cohesin complex should also be examined in the future.

5. The decrease of the loop size from pachytene to diplotene is interesting and not seen in previous HiC papers. The authors might want to comment more on what might be happening (axis disassembly? Cohesin turnover?)

Response:
We agree with the reviewer that the reduction in loop size in diplonema is an interesting observation. We also observed a slightly higher orientation bias (Fig. 4f) and stronger pairwise CTCF interactions (Supplementary Figure 11 and 12) in diplonema than that from other examined meiotic substages. We speculate that cohesin mediated loop extrusion is slightly more active during diplonema.

Notably, the derivative curve derived from the merged diplotene data does not exhibit a typical “bump”; nevertheless, a first local maximum is detectable at approximately 700 kb. Similar flattened curves are also observed in plots generated from the biological replicates for diplonema (this plot has been included in supplementary figure 5). Interestingly, in one of our biological replicates, a second “bump” was observed at a distance of approximately 1.2 Mb following the first “bump” (~875 kb). For this reason, we labeled the diplotene loop size as a range in the current Supplementary Figure 5. The reason is not clear. One possibility is that there are two sub-populations with different loop sizes within diplonema.

Specific changes:
Lines 448-457: Added discussion on why simulated diplonema loop size might be different to that reported previously.

6. The authors provide solid evidence for a direct interaction between CTCF sites and meiotic hotspots. However, this finding is not entirely novel but confirms earlier, indirect observations: Grey et al. (Genome Research, 2018) and Biot et al. (Molecular Cell, 2024) showed that PRDM9 (ie hotspots) and CTCF can co-immunoprecipitate and that crosslink ChIP experiments for either factor reveal “shadow” peaks corresponding to the site of other, which led to speculation about a potential interaction of hotspots and CTCF sites. Moreover, in two former HiC papers, Vara et al and Zuo et al. the authors found CTCF and cohesin sites to coincide (ChIP). To my mind these former results nicely complement the current study which finally provides direct, high-resolution evidence for this interaction, strengthening our understanding of CTCF’s involvement at hotspots. It would be important for the authors to acknowledge/discuss these previous observations and situate their results in this context.

Response:
We appreciate the reviewer for drawing our attention to these related findings and apology for missing them in the current version.

Specific changes:
Line 285-288: Added context and references

7. In the same line, the authors state that they are the first to identify specific sites associated with the meiotic axis. And in line 373 they state "To date, there is no evidence for DNA sequence specificity in loop positioning in mammals". However, this is not entirely true as this question has already been addressed in several earlier studies using different approaches. For instance, one study reported an enrichment of SINE elements, suggesting that they may contribute to axis formation (He et al. Dev Cell. 2023), while another described SYCP3 enrichment at CTCF sites, promoters, and DSB sites (Biot et al. 2024). These prior observations should be acknowledged and integrated into the discussion. Importantly, this does not diminish the impact of the present study, which provides direct evidence that specific sites at loop bases themselves constitute, at least in part, the foundation of the meiotic axis, whereas former data was in part more indirect.

Response:
Thanks again for pointing out this omission. We have extended the discussion, integrating these data that indeed provides orthogonal evidence for preferred sites at the base of the loops.

Specific changes:
Lines 399-403: Added context and references

8. Line 338 the authors state that "chromatin organization that results from balancing between the dense linear loop array configuration and maintaining active transcription". Using the data from Gaysinskaya, V. et al.2018, they correlate RNA-seq data with their aggregation plots (Fig. 6E). However, this statement (not necessarily the analysis) should be refined. Transcript abundance measured by RNA-seq does not directly reflect active transcriptional activity, specifically in the context of meiosis. Indeed, a recent dataset of Bellutti et al. (2025) demonstrated that transcription is silenced and transcripts are stabilized at the initiation of meiotic prophase I, transcription is then massively reactivated in pachytene. This was demonstrated by integrating scRNA-seq with EU-seq, which quantifies nascent transcripts to assess ongoing transcription. It is thus very interesting to note that, albeit transcription itself experiences a strong variation from "on" to "off" and "on" again (during the stages Cheng et al examined the contact maps), P-P and P-E interactions hardly change. The authors might consider highlighting this observation in their discussion and add the reference of Bellutti et al. DOI: 10.1093/nar/gkaf146

Response:
Thank the reviewer for drawing our attention to the work of Bellutti et al. The reviewer raises a very important point. We agree that the argument regarding "maintaining active transcription" is not precise. This sentence has been revised as "Our data revealed a real-world example in which active transcription is sustained despite the absence of TADs and genome-wide compartmentalization."

An association between promoter-promoter (P-P) interactions and transcription activity has been reported previously (Tsung-Han S. Hsieh et al.,DOI: 10.1016/j.molcel.2020.03.002). In our data, we also observed the association between P-P interaction and mRNA levels, despite the fact that

transcription is largely suppressed during early meiosis I prophase. Two reasons may account for this observation (not mutually exclusive).

First, H3K4me3, an active promoter mark, is maintained throughout meiosis I prophase, and its enrichment at transcription start sites (TSS) is positively correlated with their interactions (The figure is included as a supplementary figure 22). A previous work from our lab also showed a positive correlation between H3k4me3 enrichment at TSS and mRNA levels (DOI: 10.1038/s41467-019-11820-7). This raises a possibility that the observed association between P-P interactions and mRNA abundance may be indirect and mediated by H3K4me3.

Second, the mechanism underlying the establishes/maintains interactions between regulatory elements is not fully understood at this moment. Deletion of CTCF, cohesin, WAPL, YY1 and RNA polymerase II has minimal effect on these interactions. And a most published recent paper (Viraat Y. Goel et al., DOI: 10.1038/s41594-025-01687-2) and a preprint paper (Allana Schooley et al., DOI: 10.1101/2024.09.16.613305) both detected extensive interactions between cis-regulatory elements before cells exit mitosis, at which time point broad gene expression has not been resumed. This evidence indicate that interactions between regulatory elements are stably maintained after their establishment. These findings raise another possibility that the association between P-P interactions and mRNA abundance is established in cells preceding meiosis I prophase and persist when active transcription is suppressed.

Specific changes:
Lines 359-378: Discussion has been revised to include these observations.
New Supplementary Figure 22

Additional, minor points

When discussing genome organization at different scales, it would be helpful if the authors provided approximate size ranges for each structural level. Including a chromosome schematic with highlighted regions under analysis in figures, such as some of the contact maps, is not mandatory would maybe improve clarity.

Line 51: The reference of Müller et al. (DOI: 10.15252/msb.20188293) is missing and ref 11 would better fit line 40.

Response:
We thank the reviewer for pointing out the reference issues. The reference to Müller et al. has been added to the manuscript, and reference 11 (now reference 9) support the statement on line 41.

Fig 3A: Dashed gray lines showing contact probabilities from samples without NIPBL. I think it is not obvious to all readers what is expected from a NIPBL null sample, this needs a short explanative sentence such as for example: When NIPBL is depleted, the ability of cohesin to

extrude loops is decreased, leading to a genome-wide loss of cohesin-mediated loops and increased compartmentalization.

Response:

We are grateful for the reviewer's suggestion to better present our data. The *P(s)* curve derived from NIPBL-deleted cells was used as a reference baseline so that differences in the "bump" across the examined stages could be more readily distinguished. We agree that this suggestion will improve clarity for the reader. The sentence has been added to the text "When looping activity is impaired due to NIPBL deficiency, the P(s) curve decays steeply at short genomic distances (Figure 3a, gray dashed line). The appearance of a "bump" above this baseline indicates the presence of cohesin-mediated looping."

Fig 3C: I am not convinced that the interval should be called "cohesin density" as really it shows loop density wouldn't it be more accurate?

Response:

This analysis was performed based on the simulation result from Johanna Gassler et al., (DOI: 10.15252/embj.201798083). In this polymer simulation of loop extrusion, one of the parameters to be tested is "linear density of loop-extruding cohesin". Following the above work, we used "cohesin density" in our manuscript.

Fig S10E: lacks annotation on X axis

Response:

We thank the reviewer for pointing this out. The annotation on X axis of Fig S10E has been added.

Line 206/7: Odd sentence please correct. (First, the median distance between the two loop anchors was nearly 3 times larger than in pachynema that in mES cells (620 kb vs 235 kb)).

Response:

The sentence is corrected as "First, the median distance between the two loop anchors in pachynema was nearly 3 times larger than that in mES cells (620 kb vs 235 kb)"

Fig 4D: careful in the legend its written 100kb but on the figure it's a 150kb flanking window

Response:

We thank the reviewer for pointing out this typo. The flanking region in this plot is 100Kb and it is corrected in this figure.

Fig. S11B: The figure should be simplified for more clarity. There might be confusion: for example in the middle panel it says + + but there is also + - depicted.

Response:

Fig. S11B was used to explain the scenarios when loops are defined by CTCF with the orientation of “++”, “+-” and “-+”. We appreciate the reviewer for pointing out how confusing it is.

Specific changes:
The figure legend has been modified to guide the reader.

Figure 4 legend mix up: Legend mix up: legend F seems to fit Figure 4E, then Fig F has legend G. legend E is for figure G

<u>Response:</u>
Thank the reviewer for the careful reading. The legends have been fixed.

Figure 4G: (legend 4E?): There are distinct dots on the top line showing interactions upstream of the CTCF + site with the CTCF - site (above the green arrows), this seems to be specific to Z-D stages. Are these dots real/artefact? If real yes what are these sites? The authors might want to comment on these dots.

<u>Response:</u>
We believe these signals are artefacts. The analysis for Figure 4G was performed by first selecting CTCF sites that rank in the top quartile based on both ChIP-seq signal and motif score, followed by averaging interactions within a genomic distance range of 100–300 kb. Because of the limited number of data points, interactions from problematic regions may be overrepresented. We observe more such problematic regions in Micro-C contact maps than in Hi-C maps, likely due to the short DNA fragments generated by MNase digestion. In high-resolution views of Figure 4G, these dots (above green arrows as pointed out by the reviewer) are also visible in meiotic S phase and leptonema, although they are less prominent. The analysis from zygonema to diplonema may be more affected by such noise, as interactions within this distance range are more abundant at these stages.

Fig S13A: there is a typo in the title: “de novo” and not “de nove” loop)

<u>Response:</u>
We thank the reviewer for pointing out this typo. It has been corrected in the figure.

Line 328: typo (loo)

<u>Response:</u>
The typo has been fixed

Line 286 punctuation missing

<u>Response:</u>
This has been fixed

RESPONSE TO REVIEWERS' COMMENTS

We reiterate our thanks to both reviewers for the helpful and constructive comments. Below are our point-by-point responses:

1. The figure legend of Fig. 4g has been revised to include a brief explanation of pink and green arrows.
2. Typo in Fig. 6e has been corrected.
3. "condensin to cohesin" in Discussion section has been corrected to "cohesin to condensin".
4. We used the suggested term "relative cohesin density/activity" to replace "cohesion density" in Fig 3b, c.
5. We used the suggested term "relative quantity" to replace "relative density" in Fig. 3f and revised the figure legend to include a note "The relative quantity of REC8 and RAD21 proteins is not directly comparable due to the use of different antibodies. Only changes across stages for the same protein should be interpreted."
